# Efficacy and safety of immunosuppressive agents for adults with lupus nephritis: a systematic review and network meta-analysis

**DOI:** 10.3389/fimmu.2023.1232244

**Published:** 2023-10-13

**Authors:** Nan Jiang, Shangyi Jin, Chen Yu, Jiuliang Zhao, Qian Wang, Xinping Tian, Mengtao Li, Xiaofeng Zeng

**Affiliations:** ^1^ Department of Rheumatology and Clinical Immunology, Peking Union Medical College Hospital, Chinese Academy of Medical Sciences, Peking Union Medical College, Beijing, China; ^2^ National Clinical Research Center for Dermatologic and Immunologic Diseases (NCRC-DID), Ministry of Science & Technology, Beijing, China; ^3^ State Key Laboratory of Complex Severe and Rare Diseases, Peking Union Medical College Hospital, Beijing, China; ^4^ Key Laboratory of Rheumatology and Clinical Immunology, Ministry of Education, Beijing, China

**Keywords:** lupus nephritis, immunosuppressive, efficacy, safety, network meta-analysis

## Abstract

**Introduction:**

Various immunosuppressive regimens have been developed for the treatment of lupus nephritis (LN). This study aimed to compare the efficacy and safety of immunosuppressive regimens in adults with LN.

**Methods:**

We systematically searched the PubMed, Embase, and Cochrane Central Register of Controlled Trials databases, including conference proceedings, trial registries, and reference lists, from inception until July 10, 2022. The effects of treatment were compared and ranked using the surface under the cumulative ranking curve (SUCRA). The primary endpoint was total remission. The secondary endpoints were complete remission, systemic lupus erythematosus disease activity index (SLEDAI), relapse, all-cause mortality, end-stage renal disease (ESRD), infection, herpes zoster, ovarian failure, myelosuppression, and cancer.

**Results:**

Sixty-two trials reported in 172 studies involving 6,936 patients were included in the network meta-analysis. The combination of tacrolimus (TAC), mycophenolate mofetil (MMF), and glucocorticoid (GC) provided the best result for the total remission rate (SUCRA, 86.63%) and SLEDAI (SUCRA, 91.00%), while the combination of voclosporin (VCS) , MMF and GC gave the best improvement in the complete remission rate (SUCRA, 90.71%). The combination of cyclophosphamide (CYC), MMF and GC was associated with the lowest risk of relapse (SUCRA, 85.57%) and cancer (SUCRA, 85.14%), while the combination of obinutuzumab (OTB), MMF and GC was associated with the lowest risk of all-cause mortality (SUCRA, 84.07%). Rituximab (RTX) plus MMF plus GC was associated with the lowest risk of ESRD (SUCRA, 83.11%), while the risk of infection was lowest in patients treated with azathioprine (AZA) plus CYC plus GC (SUCRA, 68.59%). TAC plus GC was associated with the lowest risk of herpes zoster (SUCRA, 87.67%) and ovarian failure (SUCRA, 73.60%). Cyclosporine (CsA) plus GC was associated with the lowest risk of myelosuppression (SUCRA, 79.50%), while AZA plus GC was associated with the highest risk of myelosuppression (SUCRA, 16.25%).

**Discussion:**

This study showed that a combination of TAC, MMF and GC was the best regimen for improving the total remission rate. The optimal regimen for specific outcomes should be highlighted for high-risk patients.

## Introduction

Lupus nephritis (LN) is a serious complication and the most common clinical manifestation of systemic lupus erythematosus (SLE) (1), and is one of the primary causes of mortality in patients with SLE (2–4). Approximately 60% of patients with SLE could develop LN (5, 6); 5–20% of patients with LN develop kidney failure within 10 years (7). One study (8) reported that the prevalence of LN was higher in Asians than in other ethnic groups. Patients with SLE usually produce a large number of antibodies, resulting in the formation of antigen-antibody complexes deposited in the kidney, ultimately leading to kidney injury (9, 10). Treatment for LN is purposively for disease activity control and prevention of end-stage renal disease.

The main treatment goal is to control disease activity and prevent relapse and progression. Mycophenolate mofetil (MMF) or cyclophosphamide (CYC) combined with glucocorticoid (GC) is considered first-line treatment, while MMF or azathioprine (AZA) combined with low-dose GC is recommended as maintenance therapy (11–14). The 1-year response rates for these treatments range from 30.4–66.2%, and a favorable renal response is related to prognosis (15–18). Although the disease can be controlled for a long time, treatment is difficult in many patients owing to adverse events, including myelosuppression, gastrointestinal symptoms, and ovarian failure. Nearly 10% of patients with LN develop end-stage renal disease after long-term treatment, and the prognosis is relatively poor (3). Therefore, additional treatment regimens are needed to improve the prognosis of LN.

A number of immunosuppressive agents have been identified for the management of LN, including CYC, MMF, cyclosporine (CSA), tacrolimus (TAC), AZA, leflunomide (LEF), mizoribine (MZR), voclosporin (VCS), rituximab (RTX), belimumab (BLM), abatacept (ABA), anifrolumab (ALB), obinutuzumab (OTB) and ocrelizumab (OLB). Although systematic reviews and meta-analyses have already been performed to compare the therapeutic effects of immunosuppressive agents (19, 20), the results from prior meta-analyses were limited by the same population in different trials, and new immunosuppressive agents were not included. Therefore, there is a need to compare and rank the efficacy and safety of immunosuppressive agents for adults with LN based on direct and indirect evidence. This systematic review and network meta-analysis aimed to update and expand previous meta-analyses, and to update clinical practice, by comparing various treatments for the management of adults with LN.

## Methods

### Search strategy and selection criteria

This study was conducted in accordance with the Preferred Reporting Items for Systematic Reviews and Meta-Analyses (PRISMA) Network Meta-Analysis extension statement (21). Randomized controlled trials (RCTs) comparing the placebo-controlled or head-to-head of 20 categories of treatment regimens for LN were included. Publication language and status were not restricted. We systematically searched the PubMed, Embase, and Cochrane Central Register of Controlled Trials databases, including conference proceedings, trial registries, and reference lists, for potentially eligible trials from inception until July 10, 2022. The search terms included “lupus nephritis” and “randomized controlled trials.” The details of the PubMed search strategy are presented in **Supplementary 1**. Trials completed but not yet published were searched on ClinicalTrials.gov (United States National Institutes for Health). The reference lists of relevant reviews were also reviewed to identify eligible trials.

Study selection was performed by two reviewers based on population, intervention, comparison, outcomes, and study design. The inclusion criteria were as follows: (1) Population—adults (age ≥18 years) with LN; (2) Intervention and comparison—placebo-controlled or head-to-head of 20 categories of treatment regimens (GC; AZA plus GC; CSA plus GC; CYC plus GC; LEF plus GC; MMF plus GC; MZR plus GC; OLB plus GC; TAC plus GC; ABA plus MMF plus GC; ALB plus MMF plus GC; AZA plus CYC plus GC; BLM plus MMF/CYC plus GC (BLM plus [MMF or CYC] plus GC); CYC plus MMF/AZA/LEF plus GC (CYC plus an oral immunosuppressive agent [MMF or AZA or LEF] plus GC); MMF plus CYC plus GC; OTB plus MMF plus GC; RTX plus MMF plus GC; TAC plus MMF plus GC; VCS plus MMF plus GC and ABA plus AZA plus CYC plus GC); (3) Outcomes—the primary endpoint was total remission (complete remission [defined as return to normal serum creatinine, urinary protein excretion <0.5 g/24 h, and inactive urinary sediment following induction therapy] and partial remission [defined as a fall to <3.0 g/d protein if baseline ≥3.0 g/d or ≥50% reduction if <3.0 g/d at baseline and stabilization of serum creatine ± 25%], while the secondary endpoints included complete remission, mean change in systemic lupus erythematosus disease activity index (SLEDAI), relapse (as defined in individual studies), all-cause mortality, end-stage renal disease (ESRD), infection, herpes zoster, ovarian failure, myelosuppression, and cancer; and (4) Study design—all included studies were required to have an RCT design.

### Data collection and quality assessment

The following data were extracted by two reviewers: first author’s name, publication year, induction/maintenance therapy, country, setting, sample size, mean age, proportion of males, intervention, control, follow-up duration, and reported outcomes. The two authors assessed the methodological quality of individual trials using the Cochrane Collaboration’s tool for assessing the risk of bias, which contains seven specified domains (random sequence generation, allocation concealment, blinding of participants and personnel, blinding of outcome assessment, incomplete outcome data, selective reporting, and other bias) (22). Inconsistencies in data extraction and quality assessment were resolved by a third independent reviewer.

### Statistical analysis

The therapeutic effects on LN in each trial were treated as dichotomous data extracted before data pooling. Direct and indirect comparisons were combined to compare various treatments using network meta-analysis (23). A loop-specific approach was used to assess the differences in specific comparisons in the loop (24). The design-by-treatment interaction inconsistency model was used to assess network consistency (23). The random-effects model was used to perform a network meta-analysis owing to the heterogeneity across the included patients (23). The effects of treatment were compared and ranked using the surface under the cumulative ranking curve (SUCRA) (25). A SUCRA of 1 was considered the best, and a SUCRA of 0 was considered the worst. Pair-wise comparison analysis was performed, and odds ratio (OR) with 95% credible intervals (CrIs) were calculated. Publication bias was assessed using comparison-adjusted funnel plots with Egger and Begg’s tests (26). The R package “netmeta” was utilized in all analyses.

## Results

### Literature search and study selection

**Figure 1:**
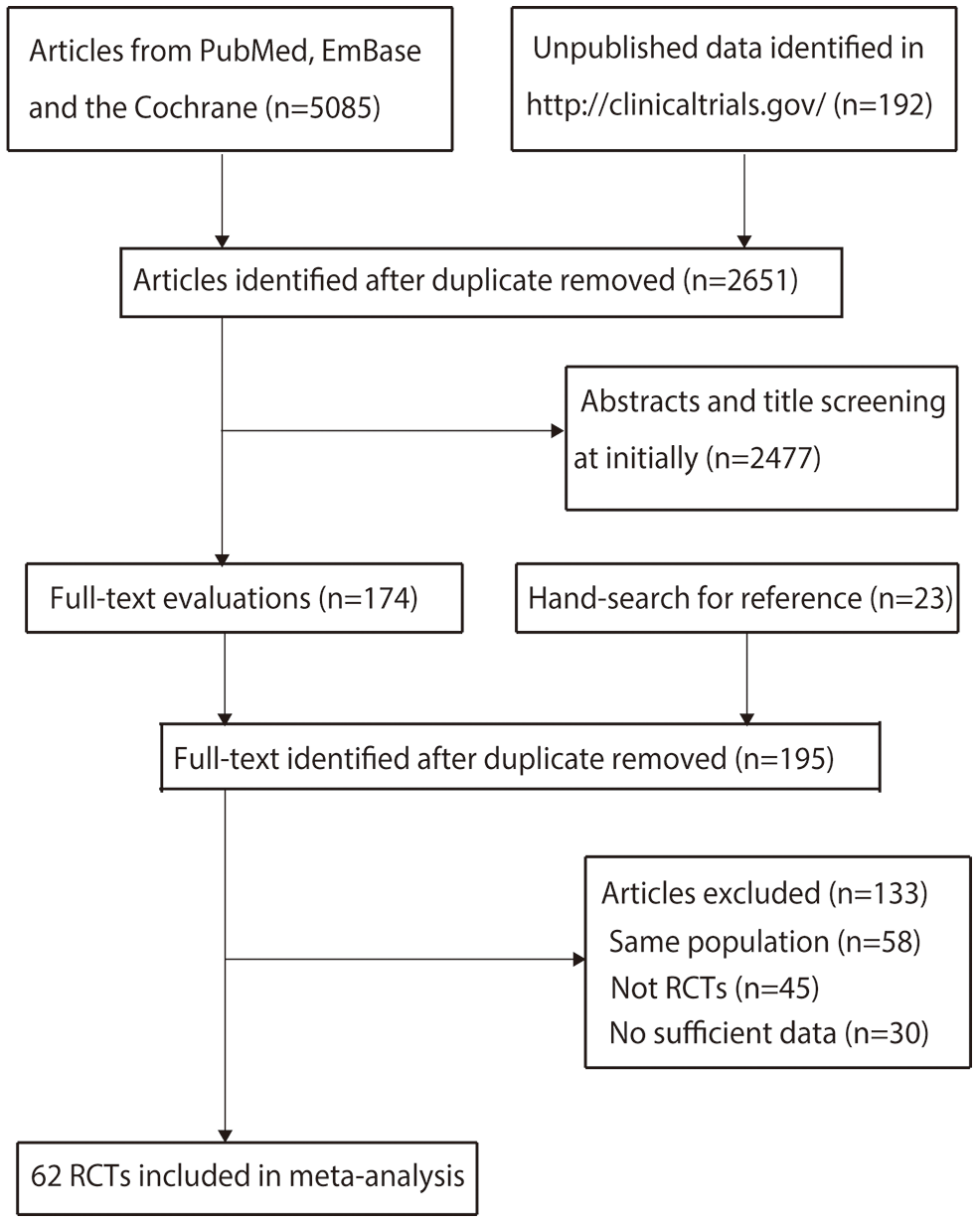
PRISMA flowchart of the literature search and study selection processes.

Details of the literature search and study selection are shown in **Figure 1**. The initial search yielded 5,085 articles in the online databases and 192 records in ClinicalTrials.gov. After removing duplicate records, 2,651 articles were retained. A total of 2,477 articles were subsequently excluded after title or abstract review. The remaining 174 articles were retrieved for full-text evaluation, and 23 studies were identified by reviewing the reference lists. After duplicate articles were removed, 195 articles were available; 133 studies were excluded because they reported on the same populations (*n* = 58), did not have an RCT design (*n* = 45), or had insufficient data (*n* = 30) (**Supplementary 2**). Finally, 62 RCTs reported in 172 articles were selected for the final network meta-analysis.

### Study characteristics

Data of all included studies and patients are shown in **Supplementary 3**, **Table S1**. A total of 6,936 patients were included. The sample size ranged from 15 to 446 patients. Thirty-seven studies were multicenter trials, while the remaining 25 studies were single-center trials. Forty-four studies investigated induction therapy, seven studies investigated maintenance therapy, and the remaining 11 studies investigated both induction and maintenance therapy. The follow-up duration ranged from 10.0 weeks to 92.4 months. The details of the methodological quality of individual trials are shown in **Supplementary 4**, **Table S1**. Overall, most of the trials were of low-to-moderate quality.

### Total remission rate

**Figure 2:**
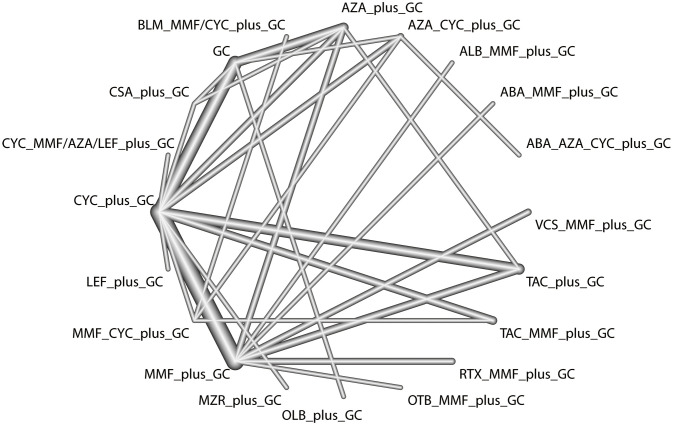
Network comparisons for the total remission rate.

**Figure 3:**
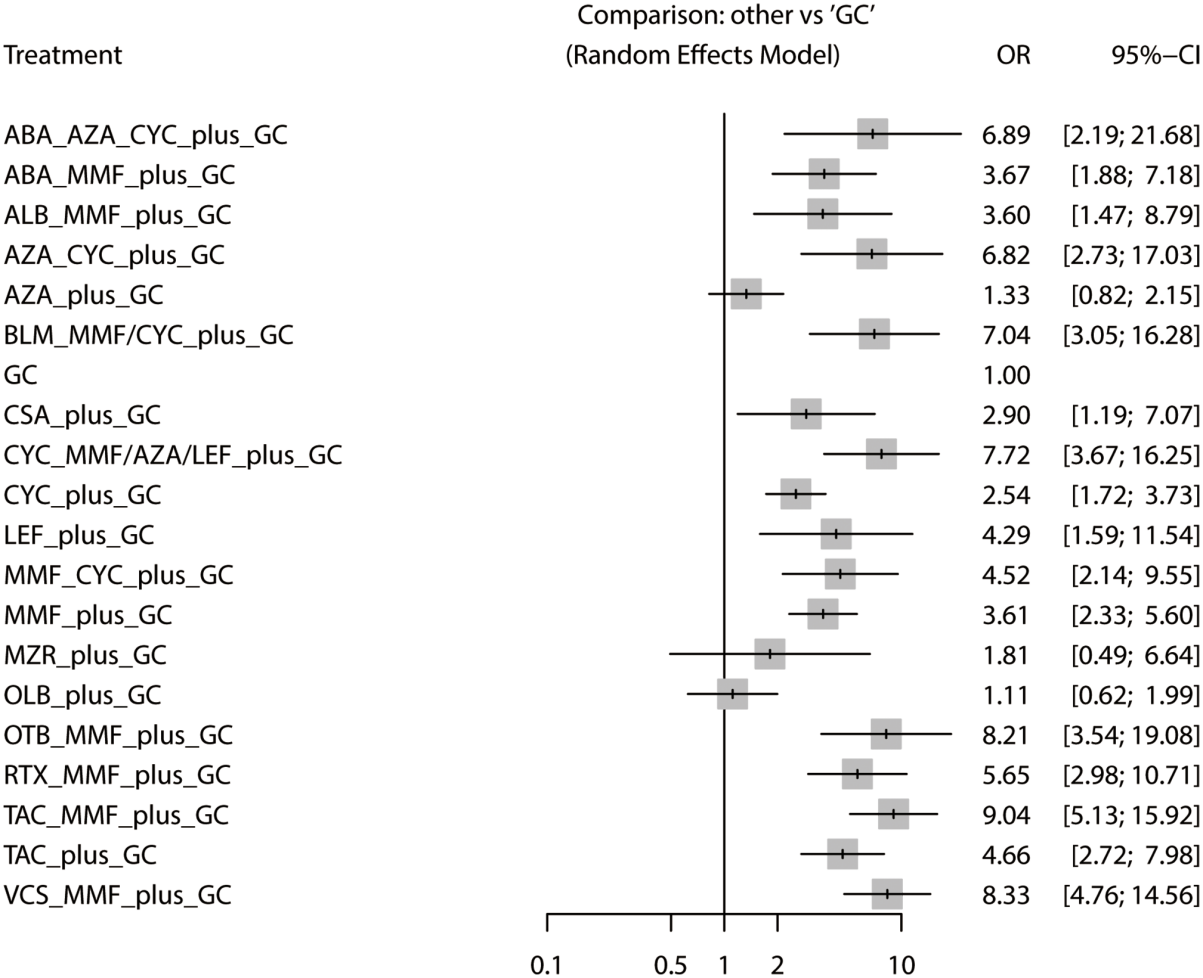
Comparison of treatment regimens and glucocorticoids on the total remission rate.

**Figure 4:**
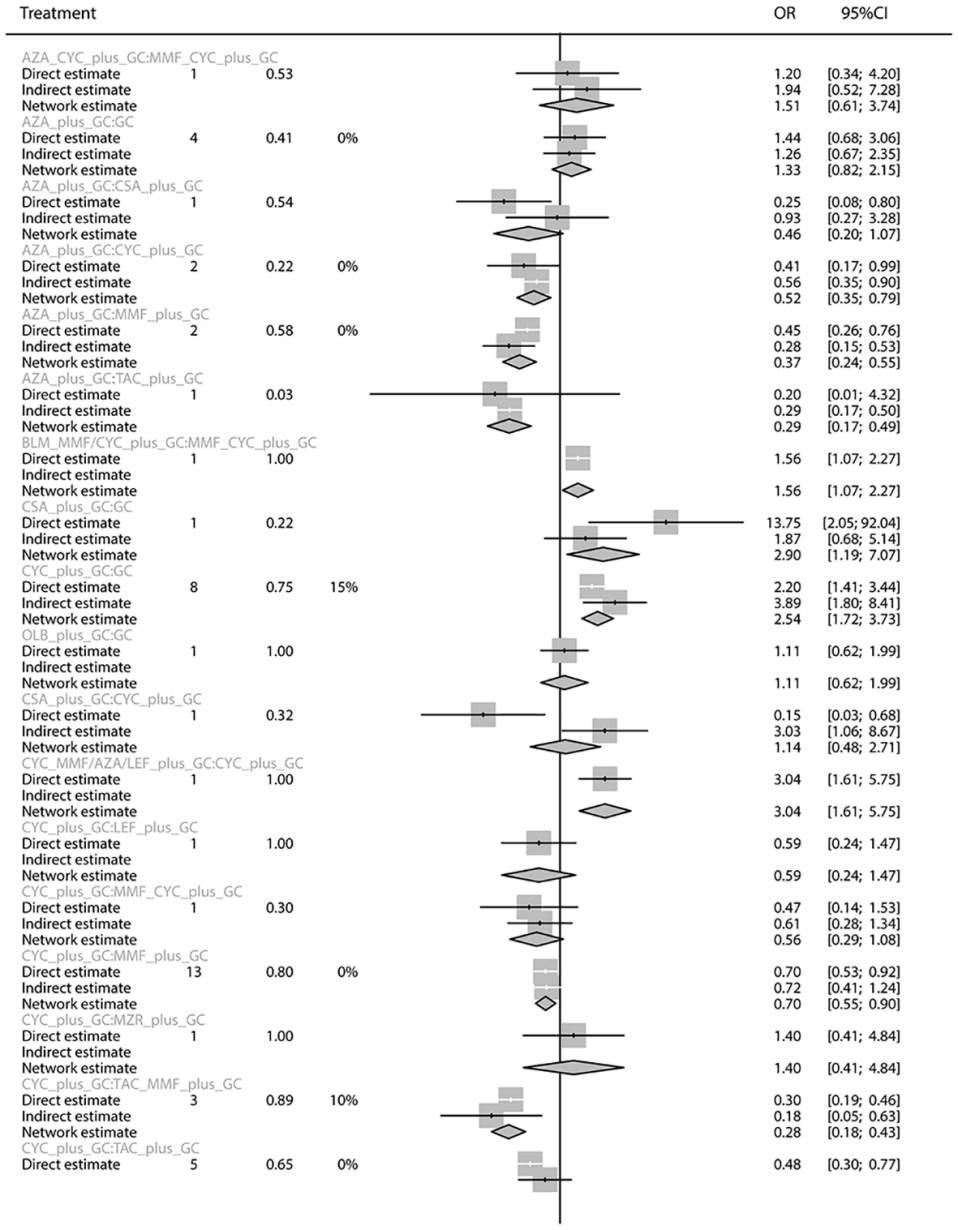
Pairwise comparison of treatment regimens for the total remission rate.

**Figure 5:**
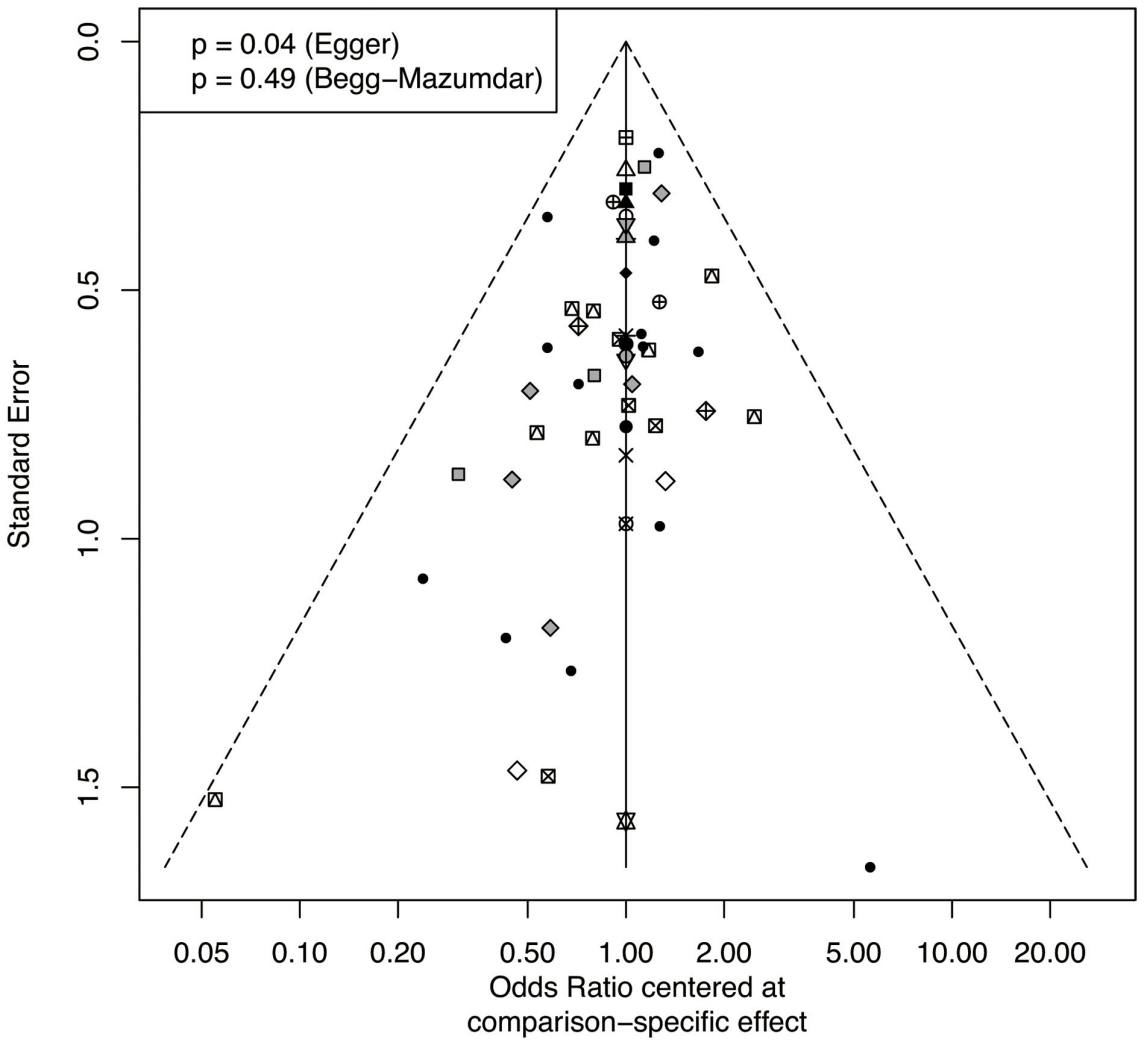
Funnel plot for the total remission rate.

The total remission rate was considered the primary outcome. The network of direct and indirect comparisons of the total remission rate in 60 trials is shown in **Figure 2**. The number of trials reported in each treatment category was weighted by the nodes, and the precision of the direct estimates for pairwise comparisons was weighted by the edges. The therapeutic effects of immunosuppressive agents on the total remission rate were compared and ranked using SUCRA. The combination of TAC, MMF and GC was associated with the highest total remission rate (SUCRA, 86.63%; **Table 1**). And among all regimens, OLB plus GC showed the lowest total remission rate (SUCRA, 6.47%; **Table 1**). The best regimen of single-agent immunosuppressive plus GC in terms of the total remission rate was TAC plus GC (SUCRA, 52.84%; **Table 1**). The results of pairwise comparisons of treatments with regard to the total remission rate are shown in **Figures 3** and **4**. Although the Begg test indicated no significant publication bias (*P_Begg_* = 0.49), the Egger test indicated potentially significant publication bias (*P_Egger_* = 0.04) for the total remission rate (**Figure 5**).

**Table 1:** The surface under the cumulative ranking probabilities for investigated outcomes.

Regimens	Total remission	Complete remission	SLEDAI	Relapse	All-cause mortality	ESRD	Infection	Herpes zoster	Ovarian failure	myelosuppression	Cancer
GC	3.79%	9.64%	—	—	37.93%	12.44%	48.88%	84.33%	74.60%	47.50%	54.00%
AZA_plus_GC	10.53%	17.71%	—	17.15%	47.50%	20.22%	27.12%	41.60%	67.40%	16.25%	15.29%
CSA_plus_GC	33.42%	63.14%	—	50.03%	—	—	54.94%	71.13%	—	79.50%	—
CYC_plus_GC	23.58%	34.86%	67.80%	52.41%	52.71%	35.56%	33.82%	56.27%	24.40%	73.50%	37.14%
LEF_plus_GC	50.42%	22.00%	65.40%	—	32.71%	—	41.88%	79.40%	—	—	—
MMF_plus_GC	39.37%	57.64%	37.20%	67.46%	57.00%	48.56%	62.76%	51.40%	40.00%	—	39.14%
MZR_plus_GC	20.26%	35.50%	11.80%	—	—	—	41.53%	—	—	—	—
OLB_plus_GC	6.47%	3.21%	—	—	54.00%	—	30.24%	—	—	—	—
TAC_plus_GC	52.84%	58.07%	26.80%	49.52%	63.79%	52.11%	65.53%	87.67%	73.60%	—	53.71%
ABA_MMF_plus_GC	43.00%	—	—	—	76.14%	52.11%	41.82%	8.13%	—	—	—
ALB_MMF_plus_GC	44.11%	86.57%	—	—	—	—	—	27.20%	—	—	—
AZA_CYC_plus_GC	71.53%	—	—	78.94%	49.79%	65.44%	68.59%	29.13%	20.00%	—	67.29%
MMF_CYC_plus_GC	51.58%	60.93%	—	85.57%	19.86%	58.89%	40.35%	39.07%	—	—	85.14%
OTB_MMF_plus_GC	80.89%	83.79%	—	—	84.07%	—	—	38.07%	—	—	—
RTX_MMF_plus_GC	65.58%	50.07%	—	—	31.79%	83.11%	61.06%	65.13%	—	—	—
TAC_MMF_plus_GC	86.63%	76.14%	91.00%	—	—	—	45.76%	68.47%	—	33.25%	—
VCS_MMF_plus_GC	85.05%	90.71%	—	—	51.07%	—	55.35%	—	—	—	—
ABA_AZA_CYC_plus_GC	74.84%	—	—	—	69.57%	—	67.00%	—	—	—	—
BLM_MMF/CYC_plus_GC	74.68%	—	—	—	22.07%	71.56%	58.65%	29.20%	—	—	48.29%
CYC_MMF/AZA/LEF_plus_GC	81.42%	—	—	—	—	—	54.71%	23.80%	—	—	—

### Complete remission rate

The network of direct and indirect comparisons of the complete remission rate in 34 trials is shown in **Supplementary 5**, **Figure S1**. The complete remission rate was highest for patients treated with VCS plus MMF plus GC (SUCRA, 90.71%; **Table 1**). The results of pairwise comparisons of treatments with regard to the complete remission rate are shown in **Supplementary 5**, **Figures S2**, **S3**. Most regimens of double immunosuppressive agents plus GC (except RTX plus MMF plus GC) were associated with a higher complete remission rate compared to MMF plus GC and CYC plus GC. There was no significant publication bias (*P_Egger_* = 0.69; *P_Begg_* = 0.22) for the complete remission rate (**Supplementary 5**, **Figure S4**).

### SLEDAI

The network of direct and indirect comparisons of mean change in SLEDAI in seven trials is shown in **Supplementary 6**, **Figure S1**. The combination of TAC, MMF, and GC was associated with the best SLEDAI (SUCRA, 91.00%; **Table 1**). The results of pairwise comparisons of treatments with regard to SLEDAI are shown in **Supplementary 6**, **Figures S2**, **S3**. MZR plus GC was associated with a significantly poorer SLEDAI compared with CYC plus GC (MD: 4.96; 95%CrI: 0.81-9.11). Publication bias was not assessed owing to the small number of included trials (**Supplementary 6**, **Figure S4**).

### Relapse

The network of direct and indirect comparisons of the risk of relapse in nine trials is shown in **Supplementary 7**, **Figure S1**. The optimal treatment regimens for preventing relapse were MMF-based regimens, including MMF plus CYC plus GC (SUCRA, 85.57%) and MMF plus GC (SUCRA, 67.46%) (**Table 1**). The results of pairwise comparisons of treatments with regard to the risk of relapse are shown in **Supplementary 7**, **Figures S2**, **S3**. AZA plus GC was associated with an increased risk of relapse compared with MMF plus GC. No significant publication bias (*P_Egger_* = 0.94; *P_Begg_* = 0.47) for the risk of relapse was observed (**Supplementary 7**, **Figure S4**).

### All-cause mortality

The network of direct and indirect comparisons of the risk of all-cause mortality in 37 trials is shown in **Supplementary 8**, **Figure S1**. The risk of all-cause mortality was lowest in patients treated with OTB plus MMF plus GC (SUCRA, 84.07%; **Table 1**). The best regimen of single-agent immunosuppressive plus GC for reducing the risk of all-cause mortality was TAC plus GC (SUCRA, 63.79%). The results of pairwise comparisons of treatments with regard to all-cause mortality are shown in **Supplementary 8**, **Figures S2**, **S3**. No significant differences in the risk of all-cause mortality were observed among the regimens. No significant publication bias (*P_Egger_* = 0.83; *P_Begg_* = 0.56) for the risk of all-cause mortality was observed (**Supplementary 8**, **Figure S4**).

### ESRD

The network of direct and indirect comparisons of the risk of ESRD in 24 trials is shown in **Supplementary 9**, **Figure S1**. We noted RTX plus MMF plus GC was associated with the lowest risk of ESRD (SUCRA, 83.11%; **Table 1**), and the best regimen of single-agent immunosuppressive plus GC for preventing risk of ESRD was TAC plus GC (SUCRA, 52.11%). The results of pairwise comparisons of treatments with regard to the risk of ESRD are shown in **Supplementary 9**, **Figures S2**, **S3**. No significant publication bias (*P_Egger_* = 0.70; *P_Begg_* = 0.25) for the risk of ESRD was observed (**Supplementary 9**, **Figure S4**).

### Infection

The network of direct and indirect comparisons of the risk of infection in 47 trials is shown in **Supplementary 10**, **Figure S1**. The risk of infection was lowest in patients treated with AZA plus CYC plus GC (SUCRA, 68.59%; **Table 1**), and the best regimen of single-agent immunosuppressive plus GC for preventing risk of infection was TAC plus GC (SUCRA, 65.53%). The results of pairwise comparisons of treatments with regard to the risk of infection are shown in **Supplementary 10**, **Figures S2**, **S3**. We noted CYC plus GC was associated with an increased risk of infection as compared with MMF plus GC. No significant publication bias (*P_Egger_* = 0.41; *P_Begg_* = 0.69) for the risk of ESRD was observed (**Supplementary 10**, **Figure S4**).

### Herpes zoster

Herpes zoster was analyzed independently, because it is a major manifestation of infection. The network of direct and indirect comparisons of the risk of herpes zoster in 32 trials is shown in **Supplementary 11**, **Figure S1**. TAC plus GC was associated with the lowest risk of herpes zoster (SUCRA, 87.67%; **Table 1**). The results of pairwise comparisons of treatments with regard to the risk of herpes zoster are shown in **Supplementary 11**, **Figures S2**, **S3**. AZA plus CYC plus GC, and CYC plus GC were associated with a higher risk of herpes zoster than GC alone. CYC plus GC and MMF plus GC were associated with an increased risk of herpes zoster compared with TAC plus GC. No significant publication bias (*P_Egger_* = 0.28; *P_Begg_* = 0.95) for the risk of herpes zoster was observed (**Supplementary 11**, **Figure S4**).

### Ovarian failure

The network of direct and indirect comparisons of the risk of ovarian failure in six trials is shown in **Supplementary 12**, **Figure S1**. GC monotherapy (SUCRA, 74.60%) and TAC plus GC (SUCRA, 73.60%) had relatively better effects on the risk of ovarian failure (**Table 1**). The results of pairwise comparisons of treatments with regard to the risk of ovarian failure are shown in **Supplementary 12**, **Figures S2**, **S3**. Most treatments did not increase the risk of ovarian failure. However, CYC plus GC was associated with a higher risk of ovarian failure than GC alone (OR, 3.70 [95% CrIs: 1.54–8.87]). Publication bias was not assessed owing to the small number of included trials (**Supplementary 12**, **Figure S4**).

### Myelosuppression

The network of direct and indirect comparisons of the risk of myelosuppression in six trials is shown in **Supplementary 13**, **Figure S1**. The risk of myelosuppression was highest for patients treated with AZA plus GC (SUCRA, 16.25%) and lowest for those treated with CSA plus GC (SUCRA, 79.50%; **Table 1**). The results of pairwise comparisons of treatments with regard to the risk of myelosuppression are shown in **Supplementary 13**, **Figures S2**, **S3**. No significant differences in the risk of myelosuppression were observed among the regimens. Publication bias was not assessed owing to the small number of included trials (**Supplementary 13**, **Figure S4**).

### Cancer

The network of direct and indirect comparisons of the risk of cancer in 10 trials is shown in **Supplementary 14**, **Figure S1**. The risk of cancer was lowest in patients treated with MMF plus CYC plus GC (SUCRA, 85.14%; **Table 1**). The best regimen of single-agent immunosuppressive plus GC for cancer prevention was TAC plus GC (SUCRA, 53.71%). The results of pairwise comparisons of treatments with regard to the risk of cancer are shown in **Supplementary 14**, **Figures S2**, **S3**. No significant differences in the risk of cancer were observed among the regimens. No significant publication bias (*P_Egger_* = 0.82; *P_Begg_* = 0.39) for the risk of cancer was observed (**Supplementary 14**, **Figure S4**).

## Discussion

The current updated systematic review and network meta-analysis comprised 62 RCTs and 6,936 patients with LN who were treated with 20 categories of treatment regimens. A review of prior systematic reviews (19, 20) identified a total of 65 RCTs. These studies suggested that CYC plus GC, MMF plus GC, and TAC plus GC were associated with greater renal response compared with GC alone. Moreover, MMF plus GC, CYC plus GC, TAC plus GC, and CYC plus AZA plus GC were associated with an increased risk of herpes zoster. However, we noted that several RCTs reported on the same population, which may overestimate the therapeutic effects of immunosuppressive agents in LN. Furthermore, the identified trials included both children and adults. The heterogeneity across trials may affect the therapeutic effects of immunosuppressive agents. In addition, several new regimens for the treatment of LN were not included; thus, the current study was performed to compare and rank the efficacy and safety of immunosuppressive agents for LN using a network meta-analysis.

Our study found that TAC plus MMF plus GC provided the best therapeutic effect in terms of the total remission rate, and SLEDAI. TAC plus GC was associated with the highest total remission rate, and complete remission among the regimens of single-agent immunosuppressive plus GC. The VCS plus MMF plus GC regimen showed the best effect in terms of the complete remission rate. TAC, a calcineurin inhibitor, inhibits human T-cell proliferation. The protective effects of calcineurin inhibitors on glomerular podocytes were independent of the immunosuppressive effects (27–29). VCS, a next-generation calcineurin inhibitor, has up to a 4-fold greater potency than CsA. The increased potency and decreased metabolite exposure of VCS resulted in greater pharmacokinetic and pharmacodynamic predictability (30). 2019 Update of the Joint European League Against Rheumatism and European Renal Association-European Dialysis and Transplant Association (EULAR/ERA-EDTA) recommendations for the management of lupus nephritis recommends that the combination of MMF with a CNI (calcineurin inhibitor, especially TAC) is an alternative, particularly in patients with nephrotic-range proteinuria (14). Also, KDIGO 2021 clinical practice guideline for the management of glomerular diseases suggests that initial therapy with a regimen of double immunosuppressive agents plus GC that includes a CNI (TAC or CsA) with reduced-dose MPAA (mycophenolic acid analogs) and glucocorticoids is reserved for patients who cannot tolerate standard-dose MPAA or are unfit for or will not use cyclophosphamide-based regimens (31).

This study found that MMF-based regimens, such as MMF plus CYC plus GC, OTB plus MMF plus GC and RTX plus MMF plus GC were optimal for preventing relapse, all-cause mortality and ESRD. The best regimen of single-agent immunosuppressive plus GC in terms of relapse all-cause mortality, and ESRD were observed for MMF plus GC and TAC plus GC. In most cases, no significant differences in these outcomes were observed among regimens. Several reasons may explain these results: (1) considering that relapse occurred in patients in remission, the risk of relapse is related to total remission; (2) the goals of induction and maintenance therapy differ and are related to prognosis; and (3) the incidence of all-cause mortality was lower than expected; statistical power was not sufficient to detect differences between regimens.

This study showed AZA plus CYC plus GC was associated with lowest risk of infection, whereas this result might due to change owing to smaller number of included trials reported infection risk for patients treated with AZA plus CYC plus GC. Moreover, the optimal treatment for herpes zoster was TAC plus GC. Herpes zoster is an infectious disease that is associated with LN owing to the autoimmune nature of the disease (32). The risk of ovarian failure was lower in patients treated with TAC plus GC, while the risk of myelosuppression was lowest in patients treated with CSA plus GC. Most pairwise comparisons were not significant, which may be explained by the small number of included trials. However, AZA plus CYC plus GC was associated with an increased risk of ovarian failure, while AZA plus GC was associated with an increased risk of myelosuppression. These adverse events should be cautiously monitored. The risk of cancer was lowest for patients treated with MMF plus CYC plus GC, while TAC plus GC was associated with the lowest risk of cancer among the regimens of single-agent immunosuppressive plus GC. We did not detect significant differences in cancer risk among regimens, which may be explained by the low incidence of cancer; statistical power was not sufficient to detect differences between regimens.

According to our results, CNI-based treatment regimens and MMF-based regimens showed advantages in clinical efficacy, which are consistent with our clinical experience and the guideline recommendations. Among the regimens of single-agent immunosuppressive plus GC, TAC plus GC performed best in avoiding death and ESRD. The use of CYC should be carefully evaluated in patients with fertility needs owing to the gonadotoxic nature. Myelosuppression is a serious adverse reaction due to AZA and the results of this study also suggested that AZA plus GC had the greatest risk of infection and myelosuppression, thus patients using AZA should be intensively monitored for leukocytopenia and infection. Furthermore, AZA plus GC was associated with a greater risk of cancer development, although this result requires further validation, it suggests that we should be cautious when prescribing AZA in patients with a history of cancer.

The strengths of this study include the following: (1) all included studies were RCTs, which may eliminate selection and confounding biases related to observational studies; (2) the analysis included 20 categories of treatment regimens, and the comprehensive results included both efficacy and safety outcomes; and (3) this study included a large sample size, and the results of this study were more robust than those of individual trials.

Several limitations of this systematic review and network meta-analysis should be acknowledged. First, stratified data according to race, sex, age, histological class and activity of LN, and follow-up duration were not available in a number of included studies, which restricted us to perform an exploratory analysis to identify the influence of these factors on the efficacy of immunosuppressive agents. Second, the dose of immunosuppressive agents differed across the included trials, and the therapeutic effects were different for LN. Third, total remission included complete and partial remission, which could have affected the optimal treatment of patients with LN. Fourth, background therapies using the hydroxychloroquine were not reported in mostly included trials, which restricted us conducting more detailed analyses. Fifth, the analysis included both induction and maintenance therapy, and treatment duration varied across the included trials. Sixth, the analysis was based on published articles and publication bias was inevitable. Finally, analysis using pooled data and detailed analyses were restricted.

## Conclusions

This study reported the optimal treatments for each investigated outcome in patients with LN. Overall, the combination of TAC or VCS with MMF and GC provided the best effects in terms of total and complete remission rates among all regimens, while TAC plus GC provided optimal therapeutic effects and was associated with a lower incidence of adverse events among the regimens of single-agent immunosuppressive plus GC. Further large-scale RCTs should be performed to directly compare the therapeutic effects of various immunosuppressive agents in patients with LN.

## Data availability statement

The original contributions presented in the study are included in the article/**Supplementary Material**. Further inquiries can be directed to the corresponding author.

## Author contributions

X-FZ conceived, designed and coordinated the study, and critically revised the manuscript. NJ, SJ and CY contributed to study design, data collection, analysis and interpretation, and drafted the manuscript. JZ, QW, XT and ML contributed to data acquisition, analysis and interpretation. All authors contributed to the article and approved the submitted version.
