# Supplementary material for: Efficacy and safety of immunosuppressive agents for adults with lupus nephritis: a systematic review and network meta-analysis
Source: Front Immunol. 2023 Oct 13;14:1232244. doi: 10.3389/fimmu.2023.1232244 (PMC10611487; doi:10.3389/fimmu.2023.1232244)
Supplement: Supplementary file 1 [file DataSheet_1.zip › Supplement 1.docx]

**Search strategy in PubMed:**

(Lupus[text word] OR "Lupus Vulgaris"[MeSH] OR "Lupus Erythematosus, Cutaneous"[MeSH] OR "Lupus Erythematosus, Systemic"[Mesh]) AND ("Kidney Diseases"[MeSH] OR nephropath*[text word] OR Transplants[MeSH] OR Transplantation[MesH] OR transplantation[subheading] OR transplant*[text word] OR "Kidney"[Mesh] OR Kidney*[text word] OR Renal*[text word] OR "End Stage Renal Disease"[text word] OR ESRD[text word] OR Glomerulonephr*[text word] OR "GN"[text word] OR "crescentic GN"[text word]) AND (randomized controlled trial [pt] OR controlled clinical trial [pt] OR randomized [tiab] OR placebo [tiab] OR clinical trials as topic [mesh: noexp] OR randomly [tiab] OR trial [ti]) NOT (animals [mh] NOT humans [mh])

(((((("Lupus Erythematosus, Systemic"[Mesh] OR Systemic Lupus Erythematosus OR Lupus Erythematosus Disseminatus OR Libman-Sacks Disease OR Disease, Libman-Sacks OR Libman Sacks Disease)) OR ("Lupus Nephritis"[Mesh] OR Lupus Glomerulonephritis OR Nephritis, Lupus OR Lupus Nephritides OR Nephritides, Lupus OR Glomerulonephritis, Lupus OR Glomerulonephritides, Lupus OR Lupus Glomerulonephritides)) OR ("Lupus Vasculitis, Central Nervous System"[Mesh] OR Central Nervous System Lupus Vasculitis OR Systemic Lupus Erythematosis, Central Nervous System OR Central Nervous System Lupus OR Central Nervous System Systemic Lupus Erythematosis OR Neuropsychiatric Systemic Lupus Erythematosus OR Lupus Meningoencephalitis OR Lupus Meningoencephalitides OR Meningoencephalitides, Lupus OR Meningoencephalitis, Lupus)) OR (systemic lupus erythematosus)) OR (lupus erythematosus)) OR (systemic lupus) AND (randomized controlled trial [pt] OR controlled clinical trial [pt] OR randomized [tiab] OR placebo [tiab] OR clinical trials as topic [mesh: noexp] OR randomly [tiab] OR trial [ti]) NOT (animals [mh] NOT humans [mh])
