# Supplementary material for: Efficacy and safety of immunosuppressive agents for adults with lupus nephritis: a systematic review and network meta-analysis
Source: Front Immunol. 2023 Oct 13;14:1232244. doi: 10.3389/fimmu.2023.1232244 (PMC10611487; doi:10.3389/fimmu.2023.1232244)
Supplement: Supplementary file 1 [file DataSheet_1.zip › Supplement 2.docx]

**Study reported same population**

1. Controlled studies of oral immunosuppressive drugs in lupus nephritis. A long-term follow-up. Ann Intern Med. 1983;99(1):1-8.
2. Long-term preservation of renal function in patients with lupus nephritis receiving treatment that includes cyclophosphamide versus those treated with prednisone only. Arthritis Rheum. 1991;34(8):945-50.
3. Progressive lupus glomerulonephritis. Treatment with prednisone and combined prednisone and cyclophosphamide. Mayo Clin Proc. 1976;51(8): 484-94
4. Influence of race/ethnicity on response to lupus nephritis treatment: the ALMS study. Rheumatology (Oxford) 2010;49:128–40.
5. Mycophenolate mofetil and intravenous cyclophosphamide are similar as induction therapy for class V lupus nephritis. Kidney Int 2010;77:152–60
6. Azathioprine and cyclophosphamide in treatment of patients with diffuse proliferative lupus nephritis-a randomized controlled study [abstract]. Nephrology Dialysis Transplantation 2001;16(6):A57.
7. A preliminary study of tacrolimus versus cyclophosphamide in patients with diffuse proliferative lupus nephritis [abstract]. Nephrology Dialysis Transplantation 2007;22(Suppl 6): vi276.
8. Induction therapies for proliferative lupus nephritis: mycophenolate mofetil, tacrolimus and intravenous cyclophosphamide [abstract]. Journal of the American Society of Nephrology 2009;20:391A.
9. Mycophenolate mofetil versus tacrolimus for active lupus nephritis: an extended observation of a randomized controlled trial [abstract]. Annals of the Rheumatic Diseases 2009;68(Suppl 3):246.
10. A randomized trial comparing cyclosporine versus azathioprine for maintenance therapy in diffuse lupus nephritis [abstract]. Journal of the American Society of Nephrology 2004;15(Oct):121A.
11. Repeat kidney biopsies fail to detect differences between azathioprine and mycophenolate mofetil maintenance therapy for lupus nephritis: data from the MAINTAIN Nephritis Trial. Nephrology Dialysis Transplantation. 2012;27(5):1924-1930.
12. Long-term follow-up of a randomised controlled trial of azathioprine/methylprednisolone versus cyclophosphamide in patients with proliferative lupus nephritis. Annals of the Rheumatic Diseases. 2012;71(6):966-973.
13. Efficacy of mycophenolate mofetil in adolescent patients with lupus nephritis: evidence from a two-phase, prospective randomized trial. Lupus. 2012;21(13):1433-1443.
14. Mycophenolate Mofetil or Intravenous Cyclophosphamide for Lupus Nephritis With Poor Kidney Function: A Subgroup Analysis of the Aspreva Lupus Management Study. American Journal of Kidney Diseases. 2013;61(5):710-715.
15. Treatment of lupus nephritis with prednisone and combined prednisone and azathioprine. Ann Intern Med. 1972; 77: 829-835
16. Long-term outcome of a randomised controlled trial comparing tacrolimus with mycophenolate mofetil as induction therapy for active lupus nephritis. Ann Rheum Dis. 2020 Aug;79(8):1070-1076.
17. Long-term follow-up of the MAINTAIN Nephritis Trial, comparing azathioprine and mycophenolate mofetil as maintenance therapy of lupus nephritis. Ann Rheum Dis. 2016 Mar;75(3):526-31.
18. Extended follow-up of the CYCLOFA-LUNE trial comparing two sequential induction and maintenance treatment regimens for proliferative lupus nephritis based either on cyclophosphamide or on cyclosporine A. Lupus. 2014;23(1):69-74.
19. Journal Club: Efficacy and Safety of Voclosporin Versus Placebo for Lupus Nephritis (AURORA 1): A Double-Blind, Randomized, Multicenter, Placebo-Controlled, Phase 3 Trial. ACR Open Rheumatol. 2021 Dec;3(12):827-831.
20. Update on the Efficacy and Safety Profile of Voclosporin: An Integrated Analysis of Clinical Trials in Lupus Nephritis. Arthritis Care Res (Hoboken). 2022 Aug 30. doi: 10.1002/acr.25007.
21. Evidence for the superiority of immunosuppressive drugs and prednisone over prednisone alone in lupus nephritis. Results of a pooled analysis. N Engl J Med. 1984 Dec 13;311(24):1528-33.
22. Sequential therapy for diffuse proliferative and membranous lupus nephritis: cyclophosphamide and prednisolone followed by azathioprine and prednisolone. Nephron 1995;71:321-327
23. Ciclosporin plus steroids versus steroids alone in the treatment of lupus nephritis. Contrib Nephrol. 1992;99:129-30.
24. Progression and remission of renal disease in the Lupus Nephritis Collaborative Study. Results of treatment with prednisone and short-term oral cyclophosphamide. Ann Intern Med. 1992 Jan 15;116(2):114-23.
25. Termination of a clinical trial with no treatment group difference: the Lupus Nephritis Collaborative Study. Control Clin Trials. 1992 Feb;13(1):62-79.
26. Role of pathology indices in the management of severe lupus glomerulonephritis. Lupus Nephritis Collaborative Study Group. Kidney Int. 1992 Sep;42(3):743-8.
27. Therapy of lupus nephritis. A two-year prospective study. Ann Med Interne (Paris). 1994;145(5):307-11.
28. A randomized study comparing three cyclosporine-based regimens in cadaveric renal transplantation. Italian Multicentre Study Group for Renal Transplantation (SIMTRe). J Am Soc Nephrol. 1997 Apr;8(4):638-46.
29. Intravenous immunoglobulin compared with cyclophosphamide for proliferative lupus nephritis. Lancet. 1999 Aug 14;354(9178):569-70.
30. Combination therapy with pulse cyclophosphamide plus pulse methylprednisolone improves long-term renal outcome without adding toxicity in patients with lupus nephritis. Ann Intern Med. 2001 Aug 21;135(4):248-57.
31. Early response to immunosuppressive therapy predicts good renal outcome in lupus nephritis: lessons from long-term followup of patients in the Euro-Lupus Nephritis Trial. Arthritis Rheum. 2004 Dec;50(12):3934-40.
32. Immunosuppressive therapy in lupus nephritis: the Euro-Lupus Nephritis Trial, a randomized trial of low-dose versus high-dose intravenous cyclophosphamide. Arthritis Rheum. 2002 Aug;46(8):2121-31.
33. The 10-year follow-up data of the Euro-Lupus Nephritis Trial comparing low-dose and high-dose intravenous cyclophosphamide. Ann Rheum Dis. 2010 Jan;69(1):61-4.
34. Long-term study of mycophenolate mofetil as continuous induction and maintenance treatment for diffuse proliferative lupus nephritis. J Am Soc Nephrol. 2005 Apr;16(4):1076-84.
35. Conversion of ciclosporin A to tacrolimus in kidney transplant recipients with chronic allograft nephropathy. Nephrol Dial Transplant. 2006 Nov;21(11):3243-51.
36. Mycophenolate mofetil as induction and maintenance therapy for lupus nephritis: rationale and protocol for the randomized, controlled Aspreva Lupus Management Study (ALMS). Lupus. 2007;16(12):972-80.
37. Identification of biomarkers that predict response to treatment of lupus nephritis with mycophenolate mofetil or pulse cyclophosphamide. Arthritis Care Res (Hoboken). 2011 Mar;63(3):351-7.
38. Mycophenolate mofetil in the treatment of systemic lupus erythematosus. Curr Opin Rheumatol. 2011 Sep;23(5):454-8.
39. Impact of the ALMS and MAINTAIN trials on the management of lupus nephritis. Nephrol Dial Transplant. 2013 Jun;28(6):1371-6.
40. Identification of clinical and serological factors during induction treatment of lupus nephritis that are associated with renal outcome. Lupus Sci Med. 2015 May 20;2(1):e000089.
41. Clinical Outcomes Observed among Biopsy Proven Lupus Nephritis Patients Treated with Mycophenolate Mofetil as First-line Therapy. Cureus. 2017 Dec 4;9(12):e1907.
42. Predictors of treatment response in a lupus nephritis population: lessons from the Aspreva Lupus Management Study (ALMS) trial. Lupus Sci Med. 2022 May;9(1):e000584.
43. Systemic lupus erythematosus after renal transplantation: patient and graft survival and disease activity. The Dutch Working Party on Systemic Lupus Erythematosus. Ann Intern Med. 1991 Feb 1;114(3):183-8.
44. Systemic lupus erythematosus: analysis of disease activity in 55 patients with end-stage renal failure treated with hemodialysis or continuous ambulatory peritoneal dialysis. Dutch Working Party on SLE. Am J Med. 1990 Aug;89(2):169-74.
45. Treatment with cyclophosphamide delays the progression of chronic lesions more effectively than does treatment with azathioprine plus methylprednisolone in patients with proliferative lupus nephritis. Arthritis Rheum. 2007 Mar;56(3):924-37.
46. Deposition of nucleosomal antigens (histones and DNA) in the epidermal basement membrane in human lupus nephritis. Arthritis Rheum. 2003 May;48(5):1355-62.
47. A proteinuria cut-off level of 0.7 g/day after 12 months of treatment best predicts long-term renal outcome in lupus nephritis: data from the MAINTAIN Nephritis Trial. Lupus Sci Med. 2015 Nov 12;2(1):e000123.
48. Nonrenal disease activity following mycophenolate mofetil or intravenous cyclophosphamide as induction treatment for lupus nephritis: findings in a multicenter, prospective, randomized, open-label, parallel-group clinical trial. Arthritis Rheum. 2010 Jan;62(1):211-21.
49. Comparison of standard of care treatment with a low steroid and mycophenolate mofetil regimen for lupus nephritis in the ALMS and AURA studies. Lupus. 2019 Apr;28(5):591-596.
50. Performance of Modified ALMS and BLISS Criteria with Standard of Care Treatment in Two US Healthcare Systems. Arthritis Care Res (Hoboken). 2022 Sep 19. doi: 10.1002/acr.25025.
51. An evaluation of voclosporin for the treatment of lupus nephritis. Expert Opin Pharmacother. 2018 Oct;19(14):1613-1621.
52. The first year results of mizoribine/tacrolimus-based multitarget treatment for consecutive patients with lupus nephritis. Clin Exp Nephrol. 2018 Dec;22(6):1371-1378.
53. Equivalence trials in SLE research: issues to consider. Lupus 1999;8:620-6
54. Cytotoxic-drug treatment of lupus nephritis. N Engl J Med. 1984 Aug 23;311(8):528-9.
55. Cytotoxic drug treatment of lupus nephritis. Am J Kidney Dis. 1982 Jul;2(1 Suppl 1):178-81.
56. Treatment of membranous nephropathy in systemic lupus erythematosus. Nephrol Dial Transplant. 1992;7 Suppl 1:97-104.
57. Immunosuppressive drug therapy in lupus nephritis. Am J Kidney Dis. 1993 Mar;21(3):239-50.
58. Alternative modes of cyclophosphamide and azathioprine therapy in lupus nephritis. Ann Intern Med. 1982 Jun;96(6 Pt 1):728-36.

**Not RCT design**

1. Azathioprine and prednisone in the treatment of adults with lupus nephritis. Clinical, histological, and immunological changes with therapy. Medicine (Baltimore). 1970 Sep;49(5):411-32.
2. Longterm survival of lupus nephritis patients treated with azathioprine and prednisone. J Rheumatol. 1978 Fall;5(3):275-87.
3. Comparison of chlorambucil, azathioprine or cyclophosphamide combined with corticosteroids in the treatment of lupus nephritis. Br J Dermatol. 1979 Feb;100(2):113-25.
4. Cyclosporin A for the treatment of systemic lupus erythematosus. Int J Immunopharmacol. 1981;3(2):163-9.
5. Intravenous pulse methylprednisolone followed by alternate day corticosteroid therapy in lupus erythematosus: a prospective evaluation. J Rheumatol. 1985 Oct;12(5):944-8.
6. Clinical outcome of three discrete histologic patterns of injury in severe lupus glomerulonephritis. Am J Kidney Dis. 1989 Apr;13(4):273-83.
7. Intermittent intravenous cyclophosphamide therapy for lupus nephritis. J Pediatr. 1989 Jun;114(6):1055-60.
8. Intermittent intravenous pulse cyclophosphamide treatment in systemic lupus erythematosus. Indian J Med Res. 1992 Apr;96:101-8.
9. Intravenous pulse cyclophosphamide treatment of severe lupus nephritis: a prospective five-year study. Clin Nephrol. 1994 Aug;42(2):71-8.
10. Cyclosporine treatment of lupus membranous nephropathy. Clin Nephrol. 1994 Sep;42(3):147-54.
11. Treatment of proliferative lupus nephritis with methylprednisolone pulse therapy and oral azathioprine. Neth J Med. 1995 Jan;46(1):4-14.
12. Treatment of the diffuse and focal proliferative forms of lupus nephropathy with intravenous cyclophosphamide. Intravenous cyclophosphamide in systemic lupus erythematosus. Rev Clin Esp. 1995 Aug;195(8):524-9.
13. Clinical effects of intermittent, intravenous cyclophosphamide in severe systemic lupus erythematosus. Nephron. 1996;74(2):313-7.
14. Outpatient monthly oral bolus cyclophosphamide therapy in systemic lupus erythematosus. J Rheumatol. 1996 Feb;23(2):273-8.
15. Cyclosporin A in the treatment of systemic lupus erythematosus: results of an open clinical study. Br J Rheumatol. 1996 Jul;35(7):669-75.
16. Cyclosporin pharmacokinetics following administration of capsules and Neoral in paediatric patients with lupus nephritis. Br J Clin Pharmacol. 1997 Aug;44(2):125-7.
17. Treatment of diffuse proliferative lupus nephritis: an Indian experience. Natl Med J India. 1997 Nov-Dec;10(6):273-5.
18. Long-term treatment of lupus nephritis with cyclosporin A. QJM. 1998 Aug;91(8):573-80.
19. Mycophenolate mofetil therapy in lupus nephritis: clinical observations. J Am Soc Nephrol. 1999 Apr;10(4):833-9.
20. Treatment of membranous lupus nephritis with nephrotic syndrome by sequential immunosuppression. Lupus. 1999;8(7):545-51.
21. Mycophenolate mofetil for the treatment of systemic lupus erythematosus: an open pilot trial. Lupus. 1999;8(9):731-6.
22. Intermittent intravenous cyclophosphamide arrests progression of the renal chronicity index in childhood systemic lupus erythematosus. J Pediatr. 2000 Feb;136(2):243-7.
23. Cyclosporine for lupus membranous nephritis: experience with ten patients and review of the literature. Lupus. 2000;9(4):241-51.
24. Assessment on intermittent intravenous cyclophosphamide pulse therapy in diffuse proliferative lupus nephritis. Ryumachi. 2000 Jun;40(3):605-11.
25. Treatment of immune nephropathies with high doses of immunoglobulins. Folia Med (Plovdiv). 2000;42(1):10-3.
26. Outcome of 85 lupus nephritis patients treated with intravenous cyclophosphamide: a single centre 10 year experience. Med J Malaysia. 2000 Mar;55(1):14-20.
27. Treatment of diffuse proliferative lupus glomerulonephritis: a comparison of two cyclophosphamide-containing regimens. Am J Kidney Dis. 2001 Aug;38(2):256-64.
28. Mycophenolate mofetil treatment of severe renal disease in pediatric onset systemic lupus erythematosus. J Rheumatol. 2001 Sep;28(9):2103-8.
29. Methylprednisolone pulse therapy in Japanese children with severe lupus nephritis. Pediatr Nephrol. 2001 Oct;16(10):817-9.
30. The safety and efficacy of MMF in lupus nephritis: a pilot study. Lupus. 2001;10(9):606-11.
31. Treatment of membranous lupus nephritis with prednisone, azathioprine and cyclosporin A. Lupus. 2001;10(11):827-9.
32. Mycophenolate mofetil for systemic lupus erythematosus refractory to other immunosuppressive agents. Rheumatology (Oxford). 2002 Aug;41(8):876-82.
33. Cyclophosphamide therapy for lupus nephritis: poor renal survival in Arab children. Pediatr Nephrol. 2003 Apr;18(4):357-61.
34. Mizoribine oral pulse therapy for patients with disease flare of lupus nephritis. Clin Nephrol. 2003 Dec;60(6):390-4.
35. Lupus nephritis: treatment with mycophenolate mofetil. Rheumatology (Oxford). 2004 Mar;43(3):377-80.
36. Long-term outcome of patients with diffuse proliferative lupus nephritis treated with prednisolone and oral cyclophosphamide followed by azathioprine. Lupus. 2005;14(4):265-72.
37. Tacrolimus for induction therapy of diffuse proliferative lupus nephritis: an open-labeled pilot study. Kidney Int. 2005 Aug;68(2):813-7.
38. Intensified, intermittent, low-dose intravenous cyclophosphamide together with oral alternate-day steroid therapy in lupus nephritis (long-term outcome). Clin Rheumatol. 2006 Nov;25(6):782-8.
39. Mycophenolate mofetil is effective in reducing lupus glomerulonephritis proteinuria. Rheumatol Int. 2006 Oct;26(12):1078-83.
40. Long-term comparison of rituximab treatment for refractory systemic lupus erythematosus and vasculitis: Remission, relapse, and re-treatment. Arthritis Rheum. 2006 Sep;54(9):2970-82.
41. Long-term effects of combination treatment with fludarabine and low-dose pulse cyclophosphamide in patients with lupus nephritis. Rheumatology (Oxford). 2007 Jun;46(6):952-6.
42. Treatment of lupus nephritis with cyclosporine - an outcome analysis. Kidney Blood Press Res. 2007;30(2):124-8.
43. Prospective study of low-dose cyclosporine A in patients with refractory lupus nephritis. Mod Rheumatol. 2007;17(2):92-7.
44. Outcomes in patients with active lupus nephritis requiring immunosuppressives who never received cyclophosphamide. J Rheumatol. 2007 Jul;34(7):1491-6.
45. Induction treatment of proliferative lupus nephritis with leflunomide combined with prednisone: a prospective multi-centre observational study. Lupus. 2008;17(7):638-44

**No sufficient data**

1. Comparison between corticosteroid and mycophenolate mofetil and corticosteroid and cyclophosphamide in the treatment of lupus nephritis [abstract no: FO031]. Nephrology Dialysis Transplantation 2007;22(Suppl 6):vi14.
2. Pendergraft WF, Tumlin JA, Rovin B, Dooley MA, Jayne DR, Wofsy D, et al. AURA-LV: successful treatment of active lupus nephritis with voclosporin [abstract no: HI-OR05]. Journal of the American Society of Nephrology 2016;27(Abstract Suppl):2B.
3. Belmont HM, Kitsis E, Skovron ML, McCullagh E, Abramson S. Misoprostol and prednisone treatment of lupus nephritis. American Journal of Therapeutics 1995;2(12):928-32
4. Deng F, Hong D, Wang L. A clinical research of the effect of leflunomide as an induction treatment for proliferative lupus nephritis [abstract no: FR-PO593]. Journal of the American Society of Nephrology 2016;27(Abstract Suppl):499A.
5. El-Sehemy MS, Al-Saaran AM, Baddour NM, Adam AG, Moez PE. Comparative clinical prospective therapeutic study between cyclophosphamide, cyclosporine and azathioprine in the treatment of lupus nephritis. Egyptian Journal of Immunology/Egyptian Association of Immunologists 2006;13(1): 39-52.
6. Flores-Suarez LF, Villa AR. Open randomized trial comparing mycophenolate mofetil (MMF) vs intravenous cyclophosphamide (IV-CYC) as induction therapy for severe lupus nephritis (LN) [abstract no: F-PO257]. Journal of the American Society of Nephrology 2004;15(Oct):122A
7. Ginzler E, Diamond H, Guttadauria M, Kaplan D. Prednisone and azathioprine compared to prednisone plus low-dose azathioprine and cyclophosphamide in the treatment of diffuse lupus nephritis. Arthritis & Rheumatism 1976;19(4):693-9
8. Liou C, Ni Z, Qian J, Lin A, Zhang W, Fang W, et al. Treatment of lupus nephritis type_and_with leflunomide: A two year prospective sequential therapies [abstract no: M-PO-1081]. 4th World Congress of Nephrology.19th International Congress of the International Society of Nephrology (ISN); 2007 Apr 21-25; Rio de Janeiro, Brazil. 2007:349.
9. Pal A, Roychowdhury A, Ghosh P. Comparison of tacrolimus azathioprine combination versus cyclophosphamide for induction treatment of proliferative lupus nephritis [abstract no: OC014]. Indian Journal of Rheumatology 2017;12(5 Suppl 1):S9
10. Rovin B, van Vollenhoven R, Aranow C, Wagner C, Gordon R, Zhuang Y, et al. A multicenter, randomized, double-blind, placebo-controlled study to evaluate the efficacy and safety of treatment with sirukumab (CNTO 136) in patients with active lupus nephritis. Arthritis & Rheumatology 2016;68(9):2174-83.
11. Yap DY, Lee P, Yam I, Tam CH, Wong S, Yung S, et al. Effect of cyclophosphamide and mycophenolate mofetil on lymphocyte subsets in patients with active lupus nephritis effect of cyclophosphamide and mycophenolate mofetil on lymphocyte subsets in patients with active lupus nephritis [abstract no: MP230]. Nephrology Dialysis Transplantation 2017;32(Suppl3):iii510.
12. Zhang JH, Yang H. Cyclophosphamide (CYX) pulse therapy in lupus nephritis (ln): short term is better [abstract]. ISN XIII International Congress of Nephrology; 1995 Jul 2-6; Madrid (Spain). 1995:290
13. A phase 2, multicenter, randomized, double-blind, placebo-controlled, proof-of-concept study to evaluate the efficacy and safety of sirukumab in patients with active lupus nephritis [abstract]. Arthritis Rheumatol. 2014; 66: S1239
14. A randomized controlled study of laquinimod in active lupus nephritis patients in combination with standard of care. Ann Rheum Dis. 2013; 72 ([abstract]): A164
15. Hong R, Haijin Y, Xianglin W, Cuilan H, Nan C, Hong R. A preliminary study of tacrolimus versus cyclophosphamide in patients with diffuse proliferative lupus nephritis. Nephrol Dial Transplant. 2007;22(Suppl 6):276.
16. Miyasaka N, Kawai S, Hashimoto H. Efficacy and safety of tacrolimus for lupus nephritis: a placebo-controlled double-blind multicenter study. Mod Rheumatol. 2009;19(6):606–615.
17. Zhang J, Zhao Z, Hu X. Effect of rituximab on serum levels of anti-C1q and antineutrophil cytoplasmic autoantibodies in refractory severe lupus nephritis. Cell Biochem Biophys. 2015;72(1):197–201.
18. A Study to Evaluate Ocrelizumab in Patients With Nephritis Due to Systemic Lupus Erythematosus (BELONG) (BELONG)
19. A Study of Mycophenolate Mofetil (CellCept) in Management of Patients With Lupus Nephritis.
20. Efficacy and Safety of Belimumab in Patients With Active Lupus Nephritis (BLISS-LN)
21. BIIB023 Proof-of-Concept Study in Participants With Lupus Nephritis (ATLAS)
22. Rituximab and Belimumab for Lupus Nephritis (CALIBRATE)
23. Safety and Efficacy on Deoxyspergualin (NKT-01) in Patients With Lupus Nephritis
24. Dose Finding, Efficacy and Safety of BI 655064 in Patients With Active Lupus Nephritis
25. Safety and Efficacy of Two Doses of Anifrolumab Compared to Placebo in Adult Subjects With Active Proliferative Lupus Nephritis (TULIP-LN1)
26. A Study to Evaluate the Safety and Efficacy of Obinutuzumab Compared With Placebo in Participants With Lupus Nephritis (LN)
27. A Study to Evaluate Efficacy of CellCept (Mycophenolate Mofetil) in Patients With Lupus Nephritis
28. Safety, Tolerability and Pharmacokinetics of Multiple Rising Doses of Ixazomib in Lupus Nephritis (LN)
29. A Study to Evaluate the Efficacy and Safety of Rituximab in Subjects With International Society of Nephrology/Renal Pathology Society (ISN/RPS) 2003 Class III or IV Lupus Nephritis (LUNAR)
30. AURA-LV: Aurinia Urinary Protein Reduction Active - Lupus With Voclosporin (AURA-LV) (AURA-LV)
