# Supplementary material for: Efficacy and safety of immunosuppressive agents for adults with lupus nephritis: a systematic review and network meta-analysis
Source: Front Immunol. 2023 Oct 13;14:1232244. doi: 10.3389/fimmu.2023.1232244 (PMC10611487; doi:10.3389/fimmu.2023.1232244)
Supplement: Supplementary file 1 [file DataSheet_1.zip › Supplement 3.docx]

Table S1. The baseline characteristics of eligibility trials and involved patients

| Study | Treatments | Country | Race | Setting | Sample size | Median/mean age (years) | Male (%) | Histological class | Intervention | Control | HCQ used | Follow-up duration | Reported outcomes |
| --- | --- | --- | --- | --- | --- | --- | --- | --- | --- | --- | --- | --- | --- |
| Steinberg 1971 ^[1]^ | I | USA | NA | Single-center | 15 | 24.0 | 0.0 | NA | CYC plus GC | GC | NA | 10.0 weeks | TRR, CR, ACM, infection |
| Cade 1973 ^[2]^ | I | USA | NA | Single-center | 54 | NA | 24.1 | NA | AZA plus GC | GC | NA | 6.0 months | TRR, ACM |
| Donadio 1974 ^[3,4]^ | I | USA | NA | Single-center | 16 | NA | 12.5 | NA | AZA plus GC | GC | NA | 6.0 months | CR, ESRD, infection |
| Hahn 1975 ^[5]^ | I | USA | Black: 13; white: 11 | Single-center | 24 | 32.5 | 16.7 | NA | AZA plus GC | GC | NA | 24.0 months | TRR, CR, ACM, ESRD, myelosuppression |
| Donadio 1978 ^[6-8]^ | I | USA | NA | Single-center | 50 | 31.5 | 18.0 | NA | CYC plus GC | GC | NA | 6.0 months | TRR, ACM, ESRD, infection, myelosuppression |
| Austin 1986 ^[9-16]^ | I | USA | NA | Multi-center | 107 | 27.0 | 14.0 | NA | AZA plus GC; CYC plus GC; AZA, CYC plus GC | GC | NA | 3.0-48.0 months | TRR, ACM, ESRD, infection, HZ, OF, cancer |
| Boumpas 1992 ^[17-19]^ | I | USA | Black: 28; other: 37 | Multi-center | 65 | 29.0 | 7.7 | III: 5; IV: 56; V: 3 | CYC plus GC | GC | NA | 6.0 months | TRR, ESRD, infection, HZ, OF, myelosuppression, cancer |
| Sesso 1994 ^[20,21]^ | I | Brazil | Black: 15; white: 14 | Single-center | 29 | 27.1 | 13.8 | II: 3; III: 1; IV: 23 | CYC plus GC | GC | NA | 15.0 months | TRR, ACM, ESRD, infection |
| Gourley 1996 ^[22,23]^ | I | USA | White: 57; Black: 18; Asian: 2; Hispanic: 5 | Single-center | 82 | 30.0 | 17.1 | III: 17; IV: 62 | CYC plus GC | GC | NA | > 60.0 months | TRR, ACM, ESRD, infection, HZ, OF, myelosuppression, cancer |
| Miyasaka 2009 ^[24]^ | I | Japan | NA | Multi-center | 63 | 36.4 | 15.9 | II: 2; III: 10; IV: 19; V: 17 | TAC plus GC | GC | NA | 28.0 weeks | Infection, SLEDAI |
| Austin 2009 ^[25-28]^ | I | USA | White: 12; other: 30 | Multi-center | 42 | 38.6 | 16.7 | NA | CYC plus GC; CSA plus GC | GC | NA | 11.0 months | TRR, CR, infection, HZ, myelosuppression, cancer |
| Mysler 2013 ^[29-31]^ | I | Multinational | White: 180; Asian: 101; Other: 100 | Multi-center | 381 | 31.3 | 12.9 | III±V: 78;  IV±V: 303 | OLB plus GC | GC | NA | 48.0 weeks | TRR, CR, ACM, infection |
| Hu 2002 ^[32]^ | I | China | Asian | Single-center | 46 | 28.8 | 17.4 | NA | MMF plus GC | CYC plus GC | NA | 6.0 months | TRR, ESRD, infection, HZ |
| Ginzler 2005 ^[33-40]^ | I | USA | White: 24; black: 79; hispanic: 28; Asian: 8; other: 1 | Single-center | 140 | 31.8 | 10.0 | III: 22; IV: 76; V: 27; mixed: 15 | MMF plus GC | CYC plus GC | NA | 24.0 weeks | TRR, CR, ACM, ESRD, infection, HZ, OF |
| Ong 2005 ^[41,42]^ | I | Malaysia | Asian | Multi-center | 44 | 30.8 | 15.9 | III:4; IV: 40 | MMF plus GC | CYC plus GC | NA | 6.0 months | TRR, CR, SLEDAI, ACM, ESRD, infection, HZ |
| Wang 2007 ^[43]^ | I | China | Asian | Single-center | 20 | 31.4 | NA | NA | MMF plus GC | CYC plus GC | NA | 6.0 months | TRR, CR, HZ |
| Chen 2007 ^[44]^ | I | China | Asian | Single-center | 25 | 30.7 | 8.0 | NA | TAC plus GC | CYC plus GC | NA | 6.0 months | TRR, CR |
| Dyadyk 2007 ^[45,46]^ | I | Ukraine | NA | Single-center | 59 | 36.0 | 15.3 | NA | AZA plus GC | CYC plus GC | NA | 20.7 months | TRR, CR, ACM |
| Mulic-Bacic 2008 ^[47]^ | I | Bosnia-Herzegovina | NA | Single-center | 45 | NA | NA | NA | MMF plus GC | CYC plus GC | NA | 6.0 months | TRR, CR, ACM |
| El-Shafey 2010 ^[48]^ | I | Egypt | NA | Single-center | 47 | 23.3 | 4.3 | III: 15; IV: 32 | MMF plus GC | CYC plus GC | NA | 6.0 months | TRR, CR, ACM, ESRD, infection, HZ |
| Chen 2011 ^[49,50]^ | I | China | Asian | Multi-center | 81 | 32.0 | 14.8 | III: 3; IV: 58; V: 9; V+III/IV: 11 | TAC plus GC | CYC plus GC | NA | 24.0 weeks | TRR, CR, ACM, infection, HZ, OF |
| Yap 2012 ^[51]^ | I | China | Asian | Multi-center | 16 | 38.3 | 37.5 | NA | TAC plus GC | MMF plus GC | NA | 24.0 months | TRR, CR, infection, HZ |
| Rathi 2016 ^[52-55]^ | I | India | Asian | Single-center | 100 | 29.5 | 8.0 | III/III+V: 17; IV/IV+V: 57; V: 26 | CYC plus GC | MMF plus GC | 100.0% | 24.0 weeks | TRR, ACM, infection, HZ, OF |
| Mok 2016 ^[56-66]^ | I | China | Asian | Multi-center | 150 | 35.5 | 8.0 | IV+V: 68; III+V: 54; V: 28 | TAC plus GC | MMF plus GC | 51.0% | 6.0 months | TRR, CR, SLEDAI, ACM, ESRD, infection, HZ, cancer |
| Mendonca 2017 ^[67]^ | I | India | Asian | Single-center | 40 | 25.8 | 20.0 | III: 2; IV: 26; V: 5; V+IV/V+III: 7 | MMF plus GC | CYC plus GC | NA | 24.0 weeks | TRR, CR, ACM, infection, HZ |
| Sedhain 2018 ^[68,69]^ | I | Nepal | Asian | Single-center | 42 | 26.0 | 11.9 | III/III+V: 9; IV/IV+V: 29; V: 4 | CYC plus GC | MMF plus GC | 100.0% | 24.0 weeks | TRR, CR, infection, HZ |
| Kamanamool 2018 ^[70-72]^ | I | Thailand | Asian | Multi-center | 83 | 32.9 | 4.8 | III/IV: 57; V or V+III/IV: 26 | TAC plus GC | MMF plus GC | NA | 6.0 months | TRR, SLEDAI, ACM |
| Zhang 2019 ^[73]^ | I | China | Asian | Multi-center | 100 | 38.7 | 5.0 | III: 18; IV: 49; III+V: 12; IV+V: 21 | LEF plus GC | CYC plus GC | NA | 24.0 weeks | TRR, CR, SLEDAI, ACM, infection, HZ |
| Zheng 2022 ^[74]^ | I | China | Asian | Multi-center | 299 | 34.2 | 12.4 | III: 16; IV: 122; V: 42; III+V: 34; IV+V: 85 | TAC plus GC | CYC plus GC | NA | 24.0 weeks | TRR, CR, ACM, infection, HZ |
| Li 2022 ^[75]^ | I | China | Asian | Multi-center | 80 | 47.9 | 20.0 | NA | TAC plus GC | CYC plus GC | NA | 12.0 months | TRR, CR |
| Li 2012 ^[76,77]^ | I | China | Asian | Single-center | 60 | 29.5 | 13.3 | III/IV: 40; III+V/IV+V: 12; V: 8 | MMF plus GC; TAC plus GC | CYC plus GC | NA | 24.0 weeks | TRR, CR, ACM, infection |
| Feng 2014 ^[78]^ | I | China | Asian | Multi-center | 90 | 31.5 | 11.1 | III: 12; IV: 20; V: 11; III/IV+V: 10 | MZR plus GC; MMF plus GC | CYC plus GC | NA | 24.0 weeks | TRR, CR, SLEDAI, infection |
| Bao 2008 ^[79,80]^ | I | China | Asian | Single-center | 40 | 28.9 | 15.0 | V+IV | MMF, TAC plus GC | CYC plus GC | NA | 6.0-9.0 months | TRR, CR, infection, HZ |
| Li 2009 ^[81,82]^ | I | China | Asian | Single-center | 19 | 39.9 | 10.5 | III: 15; IV: 4 | RTX, CYC plus GC | RTX plus GC | NA | 48.0 weeks | TRR, CR, infection， HZ |
| Rovin 2012 ^[83-94]^ | I | Multinational | White: 45; black: 40; hispanic: 52; Asian/Pacific Islander: 7 | Multi-center | 144 | 30.6 | 9.7 | III±V: 49; IV±V: 95 | RTX, MMF plus GC | MMF plus GC | NA | 52.0 weeks | TRR, CR, ACM, ESRD, infection, HZ |
| Liu 2015 ^[95]^ | I | China | Asian | Multi-center | 362 | 31.9 | 9.1 | III: 19; IV: 150; V: 69; III+V: 26; IV+V: 98 | TAC, MMF plus GC | CYC plus GC | NA | 24.0 weeks | TRR, CR, infection, HZ |
| Sun 2015 ^[96]^ | I | China | Asian | Single-center | 82 | 32.6 | 8.5 | IV | MMF, CYC plus GC | CYC plus GC | NA | 6.0 months | TRR, CR |
| Rovin 2019 ^[97]^ | I | Multinational | White: 108; black: 14; Asian: 132; other: 11 | Multi-center | 265 | 31.7 | 13.2 | V: 39; III/IV: 178; III+V/IV+V: 48 | VCS, MMF plus GC | MMF plus GC | NA | 24.0 weeks | TRR, ACM, infection |
| Zhang 2020 ^[98]^ | I | China | Asian | Single-center | 234 | NA | 43.2 | III: 57; IV: 109; V: 68 | TAC, MMF plus GC | MMF, CYC plus GC | NA | 6.0 months | TRR, CR |
| Rovin 2021 ^[99]^ | I | Multinational | White: 129; black: 45; Asian: 109; Other: 74 | Multi-center | 357 | 31.5 | 12.5 | III: 49; IV: 168; V: 50; II+V: 1; III+V: 44; IV+V: 45 | VCS, MMF plus GC | MMF plus GC | NA | 52.0 weeks | TRR, CR, ACM， infection |
| Furie 2022 ^[100]^ | I | Multinational | White: 54; American Indian or Alaska Native: 28; Black: 11; Asian: 5; Other: 27 | Multi-center | 125 | 32.5 | 15.2 | IV: 75; IV+V: 37 | OTB, MMF plus GC | MMF plus GC | NA | 52.0 weeks | TRR, CR, ACM, HZ |
| Jayne 2022 ^[101]^ | I | Multinational | White: 66; Black: 7; Asian: 28; American Indian or Alaska Native: 4; Other: 40 | Multi-center | 145 | 33.7 | 17.2 | III: 23; III+IV: 16; V: 83; IV+V: 23 | ALB, MMF plus GC | MMF plus GC | NA | 52.0 weeks | TRR, CR, HZ |
| Askanase 2014 ^[102-108]^ | I | USA | White: 67; black: 52; Asian: 6; Other: 9 | Multi-center | 134 | 32.4 | 9.0 | III: 21; IV: 48; III+V: 24; IV+V: 40 | ABA, CYC, AZA plus GC | CYC, AZA plus GC | NA | 24.0 weeks | TRR, CR, ACM， infection |
| An 2019 ^[109]^ | I | China | Asian | Multi-center | 191 | 34.3 | 8.9 | II: 9; III: 6; IV: 27; V: 8; III/IV+V: 12 | CYC, MMF/ AZA/LEF plus GC | CYC plus GC | 100.0% in intervention group | 24.0 weeks | TRR, infection, HZ |
| Moroni 2006 ^[110-114]^ | M | Italy | NA | Multi-center | 69 | 31.5 | 10.1 | IV: 60; V: 9 | CSA plus GC | AZA plus GC | NA | 48.0 months | TRR, infection |
| Dooley 2011 ^[115-140]^ | M | UK | White: 99; black: 23; Asian: 76; Other: 29 | Single-center | 227 | 31.4 | 14.1 | III/III+V: 29; IV/IV+V: 163; V: 35 | MMF plus GC | AZA plus GC | NA | 36.0 months | TRR, relapse, ACM, infection, cancer |
| Chen 2012 ^[141]^ | M | China | Asian | Multi-center | 70 | 31.9 | 12.9 | III: 54; IV: 10; V: 3; other: 3 | TAC plus GC | AZA plus GC | NA | 6.0 months | TRR, relapse, infection |
| Kaballo 2016 ^[142]^ | M | Sudan | Sudanese | Multi-center | 81 | 28.2 | 7.4 | III: 32; IV: 34; IV+V: 15 | MMF plus GC | AZA plus GC | NA | 36.0 months | TRR, relapse, ACM, ESRD, infection |
| Fu 2020 ^[143]^ | M | China | Asian | Multi-center | 215 | 32.0 | 11.6 | III/III+V: 62; IV/IV+V: 129; V: 24 | LEF plus GC | AZA plus GC | 84.6% | 36.0 months | TRR, CR |
| Contreras 2004 ^[144-148]^ | M | USA | Black: 27; hispanic: 29; White: 3 | Single-center | 59 | 32.3 | 6.8 | III: 12; IV: 46; V: 1 | CYC plus GC; AZA plus GC | MMF plus GC | NA | 27.0 months | TRR, relapse, ACM, ESRD, infection |
| Houssiau 2010 ^[149]^ | M | Multinational | Caucasian: 83; Asian: 9; black: 13 | Multi-center | 105 | 33.0 | 8.6 | III: 33; IV: 61; V: 11 | AZA, CYC, plus GC | MMF, CYC, and GC | NA | 48.0 months | TRR, relapse, ACM, ESRD, infection, HZ, cancer |
| Carette 1983 ^[150]^ | I and M | USA | NA | Multi-center | 53 | NA | 19.6 | NA | AZA plus GC; CYC plus GC | GC | NA | 85.0 months | TRR, ACM, ESRD |
| Chan 2000 ^[151-154]^ | I and M | China | NA | Multi-center | 42 | 37.5 | 7.1 | IV | MMF plus GC | CYC plus GC | NA | 63.0 months | TRR, relapse, ACM, ESRD, infection, HZ |
| Grootscholten 2006 ^[155-160]^ | I and M | Netherlands | Caucasian: 66 | Multi-center | 87 | 31.3 | 13.8 | III/V: 18; IV/V: 182 | CYC plus GC | AZA plus GC | NA | 68.4 months | TRR, relapse, ACM, ESRD, infection, HZ, cancer |
| Appel 2009 ^[161,162]^ | I and M | Multinational | White: 147; Asian: 123; Other: 100 | Multi-center | 370 | 31.9 | 15.4 | III/III+V: 58; IV/IV+V: 252; V: 60 | MMF plus GC | CYC plus GC | NA | 6.0 and 36.0 months | TRR, ACM, infection |
| Zavada 2010 ^[163,164]^ | I and M | Multinational | NA | Multi-center | 40 | 29.0 | 27.5 | III: 16; IV: 24 | CYC plus GC | CSA plus GC | NA | 92.4 months | TRR, relapse, infection, HZ |
| Mok 2001 ^[165]^ | I and M | China | Asian | Multi-center | 43 | 31.8 | 4.7 | IV | CYC, AZA plus GC | CYC plus GC | NA | 24.0 months | TRR, relapse, ESRD, infection, HZ |
| Yee 2004 ^[166]^ | I and M | Multinational | White: 9; Afro-Caribbean: 1; Asian: 1; unknown: 18 | Multi-center | 29 | 36.8 | 13.8 | III: 11; IV: 18 | CYC, AZA plus GC | CYC plus GC | NA | 24.0 months | TRR, ACM, ESRD, infection, cancer |
| Furie 2014 ^[167-169]^ | I and M | Multinational | Asian: 164; White: 111; black: 14; other: 9 | Multi-center | 298 | 31.1 | 15.8 | III: 78; IV: 220 | ABA, MMF plus GC | MMF plus GC | NA | 12.0 months | TRR, ACM, ESRD, infection, HZ |
| Furie 2020 ^[170]^ | I and M | Multinational | Asian: 223; white: 148; black: 61; other: 14 | Multi-center | 446 | 33.4 | 12.0 | III/IV: 258; III+V/IV+V: 116; V: 72 | BLM, MMF/CYC, plus GC | MMF/CYC plus GC | NA | 24.0 months | TRR, ACM, ESRD, infection, HZ, cancer |
| Atisha-Fregoso 2021 ^[171]^ | I and M | USA | White: 16; black: 18; Asian: 5; other: 4 | Multi-center | 43 | 33.4 | 14.0 | III: 2; IV: 15; III+V: 8; IV+V: 18 | BLM, RTX, CYC plus GC | RTX, CYC plus GC | 72.0% | 96.0 weeks | TRR, ESRD, infection |
| Ye 2022 ^[172]^ | I and M | China | Asian | Single-center | 56 | 30.9 | 10.7 | III+V, IV+V | TAC, MMF, plus GC | CYC plus GC | NA | 72.0 weeks | TRR, CR, SLEDAI, HZ |

*ABA: abatacept; ACM: all-cause mortality; ALB: anifrolumab; AZA: azathioprine; BLM: belimumab; CR: complete remission; ESRD: end-stage renal disease; GC: glucocorticoids; CSA: cyclosporine; CYC: cyclophosphamide; HZ: herpes zoster; HCQ: hydroxychloroquine; I: induction; LEF: leflunomide; M: maintenance; MMF: mycophenolate mofetil; MZR: mizoribine; NA: not available; OF: ovarian failure; OLB: ocrelizumab; OTB: obinutuzumab; RTX: rituximab; TAC: tacrolimus; TRR: Total remission rate; VCS: voclosporin

**Reference**

1. Steinberg AD, Kaltreider HB, Staples PJ, Goetzl EJ, Talal N, Decker JL. Cyclophosphamide in lupus nephritis: a controlled trial. Ann Intern Med. 1971; 75:165-71.
2. Cade R, Spooner G, Schlein E, Pickering M, DeQuesada A, Holcomb A, et al. Comparison of azathioprine, prednisone, and heparin alone or combined in treating lupus nephritis. Nephron. 1973;10:37-56.
3. Donadio JV Jr, Holley KE, Wagoner RD, Ferguson RH, McDuffie FC. Further observations on the treatment of lupus nephritis with prednisone and combined prednisone and azathioprine. Arthritis Rheum. 1974;17:573-81
4. Donadio JV Jr, Holley KE, Wagoner RD, Ferguson RH, McDuffie FC. Treatment of lupus nephritis with prednisone and combined prednisone and azathioprine. Ann Intern Med. 1972 Dec;77(6):829-35.
5. Hahn BH, Kantor OS, Osterland CK. Azathioprine plus prednisone compared with prednisone alone in the treatment of systemic lupus erythematosus. Report of a prospective controlled trial in 24 patients. Ann Intern Med. 1975;83:597-605
6. Donadio JV Jr, Holley KE, Ferguson RH, Ilstrup DM. Treatment of diffuse proliferative lupus nephritis with prednisone and combined prednisone and cyclophosphamide. N Engl J Med. 1978;299:1151-5
7. Donadio JV, Holley KE, Ferguson RH, Ilstrup D. Long-term treatment of diffuse proliferative lupus nephritis (DPLN) with prednisone and combined prednisone and cyclophosphamide [abstract]. Kidney Int 1977;12(6):465.
8. Donadio JV Jr, Holley KE, Ferguson RH, Ilstrup DM. Progressive lupus glomerulonephritis. Treatment with prednisone and combined prednisone and cyclophosphamide. Mayo Clin Proc. 1976;51(8):484-94.
9. Austin HA 3rd, Klippel JH, Balow JE, le Riche NG, Steinberg AD, Plotz PH, et al. Therapy of lupus nephritis. Controlled trial of prednisone and cytotoxic drugs. N Engl J Med 1986;314:614-9.
10. Austin HA, Klippel J, le Riche N, Decker JL, Balow JE. Immunosuppressive therapy of lupus nephritis [abstract]. Kidney Int 1985;27(1):204.
11. Austin HA 3rd, Muenz LR, Joyce KM, Antonovych TA, Kullick ME, Klippel JH, et al. Prognostic factors in lupus nephritis. Contribution of renal histologic data. Am J Med. 1983; 75(3):382-91.
12. Carette S, Klippel JH, Decker JL, Austin HA, Plotz PH, Steinberg AD, Balow JE. Controlled studies of oral immunosuppressive drugs in lupus nephritis. A long-term follow-up. Ann Intern Med. 1983;99(1):1-8.
13. Decker JL, Klippel JH, Plotz PH, Steinberg AD. Cyclophosphamide or azathioprine in lupus glomerulonephritis. A controlled trial: results at 28 months. Ann Intern Med. 1975; 83(5):606-15.
14. Decker JL, Steinberg AD, Reinertsen JL, Plotz PH, Balow JE, Klippel JH. NIH conference. Systemic lupus erythematosus: evolving concepts. Ann Intern Med. 1979;91(4):587-604.
15. Dinant HJ, Decker JL, Klippel JH, Balow JE, Plotz PH, Steinberg AD. Alternative modes of cyclophosphamide and azathioprine therapy in lupus nephritis. Ann Intern Med. 1982 Jun;96(6 Pt 1):728-36.
16. Steinberg AD, Steinberg SC. Long-term preservation of renal function in patients with lupus nephritis receiving treatment that includes cyclophosphamide versus those treated with prednisone only. Arthritis Rheum. 1991;34(8):945-50.
17. Boumpas DT, Austin HA 3rd, Vaughn EM, Klippel JH, Steinberg AD, Yarboro CH, et al. Controlled trial of pulse methylprednisolone versus two regimens of pulse cyclophosphamide in severe lupus nephritis. Lancet. 1992;340:741-5.
18. Austin HA, Fessler BJ, Boumpas DT, Vaughan EM, Klippel JH, Balow JE. Prognostic indicators supporting use of short courses of pulse immunosuppression for severe lupus nephritis (LN) [abstract no: 130]. Journal of the American Society of Nephrology 1995;6(3):411.
19. Illei GG, Takada K, Parkin D, Austin HA, Crane M, Yarboro CH, et al. Renal flares are common in patients with severe proliferative lupus nephritis treated with pulse immunosuppressive therapy: long-term followup of a cohort of 145 patients participating in randomized controlled studies. Arthritis Rheum. 2002;46(4):995-1002.
20. Sesso R, Monteiro M, Sato E, Kirsztajn G, Silva L, Ajzen H. A controlled trial of pulse cyclophosphamide versus pulse methylprednisolone in severe lupus nephritis. Lupus. 1994;3:107-12.
21. Sesso R, Monteiro M, Silva L, Sato E, Ajzen H. Pulse cyclophosphamide (CY) versus pulse methylprednisolone (MP) in severe lupus nephritis [abstract no: 97P]. Journal of the American Society of Nephrology 1993;4(Program &Abstracts):286.
22. Gourley MF, Austin HA 3rd, Scott D, Yarboro CH, Vaughan EM, Muir J, et al. Methylprednisolone and cyclophosphamide, alone or in combination, in patients with lupus nephritis. A randomized, controlled trial. Ann Intern Med. 1996;125: 549-57.
23. Illei GG, Austin HA, Crane M, Collins L, Gourley MF, Yarboro CH, et al. Combination therapy with pulse cyclophosphamide plus pulse methylprednisolone improves long-term renal outcome without adding toxicity in patients with lupus nephritis. Ann Intern Med. 2001;135(4):248-57.
24. Miyasaka N, Kawai S, Hashimoto H. Efficacy and safety of tacrolimus for lupus nephritis: a placebo-controlled double-blind multicenter study. Mod Rheumatol. 2009;19(6):606-15.
25. Austin HA 3rd, Illei GG, Braun MJ, Balow JE. Randomized, controlled trial of prednisone, cyclophosphamide, and cyclosporine in lupus membranous nephropathy. J Am Soc Nephrol. 2009; 20:901-11.
26. Austin HA, Balow JE. Long-term observations in a prospective clinical trial of prednisone, cyclosporine and cyclophosphamide for lupus membranous nephropathy (LMN) [abstract no: SU-FO048]. Journal of the American Society of Nephrology 2004;15(Oct):54A.
27. Austin HA, Vaughan EM, Balow JE. Lupus membranous nephropathy: randomized controlled trial of prednisolone, cyclosporine and cyclophosphamide [abstract no: A0439]. Journal of the American Society of Nephrology 2000;11:81A.
28. Austin HA, Vaughan EM, Boumpas DT, Klippel JH, Balow JE. Lupus membranous nephropathy: controlled trial of prednisolone, pulse cyclophosphamide, and cyclosporine A [abstract no: A0411]. Journal of the American Society of Nephrology 1996;7(9):1328
29. Mysler EF, Spindler AJ, Guzman R, Bijl M, Jayne D, Furie RA, et al. Efficacy and safety of ocrelizumab in active proliferative lupus nephritis: results from a randomized, double-blind, phase III study. Arthritis Rheum. 2013;65:2368-79.
30. Mysler E, Spindler A, Guzman R, Renato B. Study design and baseline patient characteristics of BELONG, the randomized double-blind, placebo-controlled phase III trial of ocrelizumab, a humanized anti-CD20 antibody, in lupus nephritis [abstract no: PO2.E20]. Lupus 2010;19(1 Suppl):156.
31. Mysler EF, Spindler AJ, Guzman R, Bijl M, Jayne D, Furie RA, et al. Efficacy and safety of ocrelizumab, a humanized antiCD20 antibody, in patients with active proliferative lupus nephritis (LN): results from the randomized, double-blind phase III BELONG Study [abstract]. Arthritis & Rheumatism 2010;62(Suppl 10):1455.
32. Hu W, Liu Z, Chen H, Tang Z, Wang Q, Shen K, et al. Mycophenolate mofetil vs cyclophosphamide therapy for patients with diffuse proliferative lupus nephritis. Chin Med J. 2002;115:705-9.
33. Ginzler EM, Dooley MA, Aranow C, Kim MY, Buyon J, Merrill JT, et al. Mycophenolate mofetil or intravenous cyclophosphamide for lupus nephritis. N Engl J Med. 2005;353:2219-28.
34. Appel G, Ginzler E, Radhakrishnan J, Aranow C, Buyon J, Dooley M, et al. Multicenter controlled trial of mycophenolate mofetil (MMF) vs intravenous cyclophosphamide (IVC) as induction therapy for severe lupus nephritis (LN) [abstract no: SA-FC171]. Journal of the American Society of Nephrology 2003;14(Nov):38A.
35. Burchardi C, Schlöndorff D. Induction therapy for active lupus nephritis: mycophenolate mofetil versus cyclophosphamide. Nat Clin Pract Nephrol. 2006;2(6):314-5.
36. Elliott JR, Manzi S. Induction therapy for active lupus nephritis: mycophenolate mofetil is superior to cyclophosphamide. Nat Clin Pract Rheumatol. 2006;2(7):354-5.
37. Killen JP. Mycophenolate mofetil or intravenous cyclophosphamide in lupus nephritis. N Engl J Med. 2006;354(7):764-5.
38. Radhakrishnan J, Ginzler E, Appel G. Mycophenolate mofetil (MMF) vs. intravenous cyclophosphamide (IVC) for severe lupus nephritis (LN): subgroup analysis of patients with membranous nephropathy (SLE-V) [abstract no: TH-FC036]. Journal of the American Society of Nephrology 2005;16:8A.
39. Radhakrishnan J, Moutzouris D, Ginzler E, Appel G. Lupus membranous nephropathy IV cyclophosphamide (IVC) vs. mycophenolate mofetil (MMF) [abstract no: SA-PO2951]. Journal of the American Society of Nephrology 2008;19(Abstracts Issue):779A.
40. Robert C, Mo S, Kim M, Ginzler EM. Should therapy go beyond the control of immediate injury? Biomarkers of the vasculature and their association with longitudinal assessments in the induction phase of a randomized multicenter trial comparing mycophenolate mofetil and intravenous cyclophosphamide [abstract no: PO1.B.31]. Lupus 2010;19(1 Suppl):55.
41. Ong LM, Hooi LS, Lim TO, Goh BL, Ahmad G, Ghazalli R, et al. Randomized controlled trial of pulse intravenous cyclophosphamide versus mycophenolate mofetil in the induction therapy of proliferative lupus nephritis. Nephrology. 2005;10:504-10.
42. Ong LM, Hooi LS, Lim TO, Goh BL, Ahmad G, Ghazalli R, et al. Randomized controlled trial of pulse intravenous cyclophosphamide versus mycophenolate mofetil in the induction therapy of proliferative lupus nephritis [abstract no: FC30041]. Nephrology 2005;10(Suppl): A121.
43. Wang J, Hu W, Xie H, Zhang H, Chen H, Zeng C, et al. Induction therapies for class IV lupus nephritis with non-inflammatory necrotizing vasculopathy: mycophenolate mofetil or intravenous cyclophosphamide. Lupus. 2007;16: 707-12.
44. Chen N, Ren H, Yu HJ, Li X, Wang XL, Hao CL. A preliminary study of tacrolimus versus cyclophosphamide in patients with diffuse proliferative lupus nephritis. Nephrol Dial Transplant 2007;22:PO380.
45. Dyadyk A, Vasilenko I, Bagriy A, Dyadyk O, Yarovaya N, Roschin Y, et al. Azathioprine and cyclophosphamide in diffuse proliferative lupus nephritis treatment-a randomized controlled study [abstract]. Ann Rheum Dis. 2007;66: A467.
46. Dyadyk A, Vasilenko I, Bagriy A, Dyadyk O, Yarovaya N, Roschin Y, et al. Azathioprine and cyclophosphamide in treatment of patients with diffuse proliferative lupus nephritis - a randomized controlled study [abstract]. Nephrology Dialysis Transplantation 2001;16(6): A57.
47. Mulic-Bacic S, Antic D, Krizic M, Hajdarovic A, Mulic E. Mycophenolate mofetil or intravenous cyclophosphamide in treatment of lupus nephritis [abstract]. Ann Rheum Dis. 2008;67:349.
48. El-Shafey EM, Abdou SH, Shareef MM. Is mycophenolate mofetil superior to pulse intravenous cyclophosphamide for induction therapy of proliferative lupus nephritis in Egyptian patients? Clin Exp Nephrol. 2010;14:214-21.
49. Chen W, Tang X, Liu Q, Chen W, Fu P, Liu F, et al. Short-term outcomes of induction therapy with tacrolimus versus cyclophosphamide for active lupus nephritis: a multicenter randomized clinical trial. Am J Kidney Dis. 2011;57: 235-44.
50. Chen W, Liu Q, Chen W, Fu P, Liao Y, Zhang J, et al. A prospective multicenter randomized trial of treatment of active lupus nephritis with tacrolimus versus cyclophosphamide/azathioprine in Chinese adults [abstract no: SU248]. World Congress of Nephrology; 2009 May 22-26; Milan (Italy). 2009.
51. Yap DY, Yu X, Chen XM, Lu F, Chen N, Li XW, et al. Pilot 24 month study to compare mycophenolate mofetil and tacrolimus in the treatment of membranous lupus nephritis with nephrotic syndrome. Nephrology. 2012;17:352-7.
52. Rathi M, Goyal A, Jaryal A, Sharma A, Gupta PK, Ramachandran R, et al. Comparison of low-dose intravenous cyclophosphamide with oral mycophenolate mofetil in the treatment of lupus nephritis. Kidney Int. 2016;89: 235-42.
53. Goyal A, Rathi M, Jha V, Sharma A, Joshi K, Nada R, et al. Randomized controlled trial of low dose intravenous cyclophosphamide versus oral mycophenolate mofetil in treatment of lupus nephritis [abstract no: FR-OR053]. Journal of the American Society of Nephrology 2013;24(Abstracts):48A-9A.
54. Rathi M, Goyal A, Gupta PK, Jaryal A, Sharma A, Jha V, et al. Randomized controlled trial of low-dose intravenous cyclophosphamide versus oral mycophenolate mofetil in treatment of lupus nephritis [abstract]. Nephrology Dialysis Transplantation 2014;29(Suppl 3): iii28-iii2.
55. Rathi M, Jaryal A, Goyal A, Sharma A, Gupta P, Gupta K. Outcomes in lupus nephritis patients previously randomized to receive either low dose cyclophosphamide versus oral mycophenolate mofetil on azathioprine maintenance [abstract no: 2941]. Arthritis & Rheumatology 2015;67(Suppl 10):2941.
56. Mok CC, Ying KY, Yim CW, Siu YP, Tong KH, To CH, et al. Tacrolimus versus mycophenolate mofetil for induction therapy of lupus nephritis: a randomised controlled trial and long-term follow-up. Ann Rheum Dis. 2016;75:30-6.
57. Mok C, Ying K, Tong K, Siu Y, To C, Yim C, et al. Mycophenolate mofetil versus tacrolimus for active lupus nephritis: an extended observation of a randomized controlled trial [abstract no: 1073]. ACR/ARHP Annual Scientific Meeting; 2008 Oct 24-29; San Francisco (CA). 2008.
58. Mok CC. Factors determining response in patients with acute lupus nephritis treated with glucocorticoids and mycophenolate mofetil (MMF) [abstract]. Arthritis and Rheumatism 2009;60(Suppl 10):923.
59. Mok CC, Ho LY, To CH, Chan KL. Factors associated with renal remission, relapse and long-term renal function decline in lupusnephritis treated with combined prednisolone and mycophenolate mofetil (MMF) or tacrolimus(TAC) [abstract no: 988]. Arthritis & Rheumatology 2015;67(10 Suppl):988.
60. Mok CC, To CH, Ying KY, Yim C, Ng WL. Factors associated with long-term renal function deterioration in lupus nephritis treated initially with combined prednisolone and mycophenolate mofetil (MMF) or tacrolimus (TAC) [abstract no: 600]. Arthritis & Rheumatism 2013;65(Suppl 10):S258-9.
61. Mok CC, Ying KY, Ng CW, Ng WL. Risk of renal flares and decline in renal function in patients with active lupus nephritis treated with mycophenolate mofetil (MMF) [abstract no: SAT0202]. Annals of the Rheumatic Diseases 2010;69(3):555.
62. Mok CC, Ying KY, Yim CW, Ng WL. Risk of renal flares and decline in renal function in patients with active lupus nephritis treated with mycophenolate mofetil (MMF) [abstract]. Arthritis and Rheumatism 2010;60(10 Suppl):477.
63. Mok CC, Ying S, Yim CW, Ng WL. Factors determining response in patients with active lupus nephritis treated with glucocorticoids and mycophenolate mofetil (MMF) [abstract no: PO2.E.17]. Lupus 2010;19(1 Suppl):155.
64. Mok CC, Ying S, Yim CW, Ng WL. Tacrolimus (TAC) versus mycophenolate mofetil (MMF) for the treatment of membranous lupus nephritis: a randomized controlled trial [abstract no: OP0069]. Annals of the Rheumatic Diseases 2010;69(3):75.
65. Mok CC, Ying S, Yim CW, Ng WL. Tacrolimus (Tac) versus mycophenolate mofetil (MMF) for the treatment of membranous lupus nephritis: a randomized controlled trial [abstract no: CS6.8]. Lupus 2010;19(1 Suppl):16.
66. Mok CC, Ying SK, Tong KH, Siu YP, To CH, Yim CW, et al. Mycophenolate mofetil versus tacrolimus for active lupus nephritis: an extended observation of a randomized controlled trial [abstract no: THU0220]. Annals of the Rheumatic Diseases 2009;68(Suppl 3):246.
67. Mendonca S, Gupta D, Ali S, Gupta P. Mycophenolate mofetil or cyclophosphamide in indian patients with lupus nephritis: Which is better? A single-center experience. Saudi J Kidney Dis Transpl. 2017;28:1069-1077.
68. Sedhain A, Hada R, Agrawal RK, Bhattarai GR, Baral A. Low dose mycophenolate mofetil versus cyclophosphamide in the induction therapy of lupus nephritis in Nepalese population: a randomized control trial. BMC Nephrol. 2018;19:175.
69. Sedhain A, Hada R, Agrawal RK, Baral A, Bhattarai GR. Effect of cyclophosphamide versus mycophenolate mofetil in induction therapy of lupus nephritis in Nepalese population [abstract no: FR-PO598]. Journal of the American Society of Nephrology 2016;27(Abstract Suppl):500A.
70. Kamanamool N, Ingsathit A, Rattanasiri S, Ngamjanyaporn P, Kasitanont N, Chawanasuntorapoj R, et al. Comparison of disease activity between tacrolimus and mycophenolate mofetil in lupus nephritis: a randomized controlled trial. Lupus. 2018;27:647-656.
71. Kamanamool N, Ingsathit A, Rattanasiri S, Ngamjanyaporn P, Kasitanont N, Chawanasuntorapoj R, et al. Comparison of disease activity between tacrolimus and mycophenolate mofetil in lupus nephritis: a randomized controlled trial. Lupus 2017;27(4): 647-56.
72. Sumethkul V. Comparison between tacrolimus (TAC) and mycophenolate mofetil (MMF) for induction of remission in lupus nephritis. clinicaltrials.gov/ct2/show/NCT01580865
73. Zhang M, Qi C, Zha Y, Chen J, Luo P, Wang L, et al. Leflunomide versus cyclophosphamide in the induction treatment of proliferative lupus nephritis in Chinese patients: a randomized trial. Clin Rheumatol. 2019;38:859-867.
74. Zheng Z, Zhang H, Peng X, Zhang C, Xing C, Xu G, et al. Effect of Tacrolimus vs Intravenous Cyclophosphamide on Complete or Partial Response in Patients With Lupus Nephritis: A Randomized Clinical Trial. JAMA Netw Open. 2022;5: e224492.
75. Li L, Du Y, Ji J, Gao Y, Shi XQ. Analysis of the safety and efficacy of tacrolimus combined with glucocorticoid in the treatment of lupus nephritis. Pak J Med Sci. 2022;38:1285-1291.
76. Li X, Ren H, Zhang Q, Zhang W, Wu X, Xu Y, et al. Mycophenolate mofetil or tacrolimus compared with intravenous cyclophosphamide in the induction treatment for active lupus nephritis. Nephrol Dial Transplant. 2012;27:1467-72.
77. Li X, Ren H, Zhang W, Xu Y, Shen P, Zhang Q, et al. Induction therapies for proliferative lupus nephritis: mycophenolate mofetil, tacrolimus and intravenous cyclophosphamide [abstract]. Journal of the American Society of Nephrology 2009;20:391A.
78. Feng X, Gu F, Chen W, Liu Y, Wei H, Liu L, et al. Mizoribine versus mycophenolate mofetil or intravenous cyclophosphamide for induction treatment of active lupus nephritis. Chin Med J (Engl). 2014;127:3718-23.
79. Bao H, Liu ZH, Xie HL, Hu WX, Zhang HT, Li LS. Successful treatment of class V+IV lupus nephritis with multitarget therapy. J Am Soc Nephrol. 2008;19: 2001-10.
80. Bao H, Xie HL, Zhang HT, Zhang X, Hu WX, Liu ZH, et al. Successful treatment of class V+IV lupus nephritis with multi-target immunosuppressive therapy [abstract no: SA-FC060]. Journal of the American Society of Nephrology 2007;18(Abstracts):48a.
81. Li EK, Tam LS, Zhu TY, Li M, Kwok CL, Li TK, et al. Is combination rituximab with cyclophosphamide better than rituximab alone in the treatment of lupus nephritis? Rheumatology. 2009;48:892-8.
82. Li EK, Tam LS, Zhu TY, Kwok CL, Leung YY, Szeto CC. Rituximab monotherapy is an effective induction therapy in proliferative lupus nephritis: a pilot study [abstract no: THU0223]. Annals of the Rheumatic Diseases 2009;68(Suppl 3):247.
83. Rovin BH, Furie R, Latinis K, Looney RJ, Fervenza FC, Sanchez-Guerrero J, et al; LUNAR Investigator Group. Efficacy and safety of rituximab in patients with active proliferative lupus nephritis: the Lupus Nephritis Assessment with Rituximab study. Arthritis Rheum. 2012;64:1215-26.
84. Appel GB, Looney RJ, Eisenberg RA, Rovin BH, Ginzler EM, Adler SG, et al. Protocol for the Lupus Nephritis Assessment with rituximab (LUNAR) Study [abstract no: F-PO1121]. Journal of the American Society of Nephrology 2006;17(Abstracts):573A.
85. Furie R, Looney J, Rovin B, Latinis K, Appel G, Sanchez-Guerrero J, et al. Efficacy and safety of rituximab (RTX) in patients (Pts) with proliferative lupus nephritis (LN): results from randomized, double-blind phase III LUNAR study at week 52 [abstract no: CS6.6]. Lupus 2010;19(1 Suppl):15.
86. Furie R, Looney J, Rovin B, Latinis K, Appel G, Sanchez-Guerrero J, et al. Efficacy and safety of rituximab in patients with proliferative lupus nephritis: results from the randomized, double-blind phase III LUNAR study [abstract no: SAT0185]. Annals of the Rheumatic Diseases 2010;69(Suppl 3):549.
87. Furie R, Looney RJ, Rovin B, Latinis KM, Appel G, Sanchez-Guerrero J, et al. Efficacy and safety of rituximab in subjects with active proliferative lupus nephritis (LN): results from the randomized, double-blind phase III LUNAR study [abstract no: 1149]. ACR/ARHP Annual Scientific Meeting; 2009 Oct 17-21; Philadelphia (PA). 2009.
88. Furie R, Rovin B, Appel G, Kamen D, Fervenza F, Spindler A, et al. Effect of rituximab (RTX) on anti-dsDNA and C3 levels and relationship to response: results from the LUNAR trial [abstract no: PO2.E.22]. Lupus 2010;19(1 Suppl):157.
89. Furie R, Rovin B, Appel G, Kamen D, Fervenza FC, Spindler A, et al. Effect of rituximab (RTX) on anti -double-stranded DNA antibody and c3 levels and relationship to response: results from the LUNAR trial [abstract no: SAT0186]. Annals of the Rheumatic Diseases 2010;69(Suppl 3):550.
90. Furie R, Rovin B, Appel G, Kamen DL, Fervenza FC, Spindler A, et al. Effect of rituximab (RTX) on anti-dsDNA and C3 levels and relationship to response: results from the LUNAR trial [abstract no: 271]. ACR/ARHP Annual Scientific Meeting; 2009 Oct 17-21; Philadelphia (PA). 2009.
91. Furie R, Rovin BH, Kamen DL, Latinis KL, Appel GB, Sanchez-Guerrero J, et al. Trial design and baseline characteristics of patients in the randomized double-blind, placebo-controlled phase III lupus nephritis assessment with rituximab study (LUNAR) [abstract no: THU-0242]. Annals of the Rheumatic Diseases 2009;68(Suppl 3):253.
92. Rovin B, Appel G, Furie R, Fervenza F, Guerrero JS, Lenz O, et al. Trial design and baseline characteristics of patients in the randomized double-blind, placebo-controlled phase III LUpus Nephritis Assessment with Rituximab study (LUNAR) [abstract no: M353]. World Congress of Nephrology; 2009 May 22-26; Milan, Italy. 2009.
93. Rovin B, Appel G, Furie R, Kamen DL, Fervenza FC, Spindler A, et al. Effect of Rituximab (RTX) on anti-dsDNA and C3 levels and relationship to response: Results from the LUNAR Trial [abstract no: F-PO1281]. Journal of the American Society of Nephrology 2009;20:406A.
94. Rovin BH, Appel G, Furie R, Looney J, Latinis K, Fervenza FC, et al. Efficacy and safety of rituximab (RTX) in subjects with proliferative lupus nephritis (LN): results from the randomized, double-blind phase III LUNAR study [abstract no: SA-FC332]. Journal of the American Society of Nephrology 2009;20:77A.
95. Liu Z, Zhang H, Liu Z, Xing C, Fu P, Ni Z, et al. Multitarget therapy for induction treatment of lupus nephritis: a randomized trial. Ann Intern Med. 2015; 162:18-26.
96. Sun J, Zhang H, Ji Y, Gui M, Yi B, Wang J, et al. Efficacy and safety of cyclophosphamide combined with mycophenolate mofetil for induction treatment of class IV lupus nephritis. Int J Clin Exp Med. 2015;8:21572-8.
97. Rovin BH, Solomons N, Pendergraft WF 3rd, Dooley MA, Tumlin J, Romero-Diaz J, et al. A randomized, controlled double-blind study comparing the efficacy and safety of dose-ranging voclosporin with placebo in achieving remission in patients with active lupus nephritis. Kidney Int. 2019;95:219-231.
98. Zhang X, Liu P, Zhang Z. Analysis of the Clinical Effects of the Combination of Mycophenolate Mofetil with Either Tacrolimus or Cyclophosphamide. Clinics (Sao Paulo). 2020;75:e1820.
99. Rovin BH, Teng YKO, Ginzler EM, Arriens C, Caster DJ, Romero-Diaz J, et al. Efficacy and safety of voclosporin versus placebo for lupus nephritis (AURORA 1): a double-blind, randomised, multicentre, placebo-controlled, phase 3 trial. Lancet. 2021;397:2070-2080.
100. Furie RA, Aroca G, Cascino MD, Garg JP, Rovin BH, Alvarez A, et al. B-cell depletion with obinutuzumab for the treatment of proliferative lupus nephritis: a randomised, double-blind, placebo-controlled trial. Ann Rheum Dis. 2022;81:100-107.
101. Jayne D, Rovin B, Mysler EF, Furie RA, Houssiau FA, Trasieva T, et al. Phase II randomised trial of type I interferon inhibitor anifrolumab in patients with active lupus nephritis. Ann Rheum Dis. 2022;81:496-506.
102. ACCESS Trial Group. Treatment of lupus nephritis with abatacept: the Abatacept and Cyclophosphamide Combination Efficacy and Safety Study. Arthritis Rheumatol. 2014;66: 3096-104.
103. Shidham GB, Birmingham DJ, Rovin B, Hebert LA. 24 hour protein: creatinine ration (24 PCR), not spot PCR (Spot PCR), should be used to monitor the treatment of severe lupus nephritis (LN): the experience of ACCESS [abstract no: FR-PO604]. Journal of the American Society of Nephrology 2016;27(Abstract Suppl):501A.
104. Shidham GB, Birmingham DJ, Rovin B, Hebert LA. Ability of spot urine protein/ creatinine ratio (spot PCR) to correctly identify the proteinuria endpoints of complete remission (CR), partial remission (PR), and treatment failure (TF) as determined by 24 hour urine PCR (24 PCR): experience of abatacept and cyclophosphamide combination eicacy and safety study (ACCESS) [abstract no: TH-PO764]. Journal of the American Society of Nephrology 2015;26(Abstract Suppl):265A.
105. Wofsy D, Askanase A, Cagnoli PC, Chatham WW, Contreras G, Dall'Era M, et al. Treatment of lupus nephritis with abatacept plus low-dose pulse cyclophosphamide followed by azathioprine (the Euro-Lupus regimen): twenty-four week data from a double-blind controlled trial [abstract no: 884]. Arthritis & Rheumatology 2013;66(10 Suppl):S379-80.
106. Wofsy D, Hillson JL, Diamond B. Abatacept for lupus nephritis: alternative definitions of complete response support conflicting conclusions. Arthritis Rheum. 2012;64(11):3660-5.
107. Wofsy D, Hillson JL, Diamond B. Comparison of alternative primary outcome measures for use in lupus nephritis clinical trials. Arthritis Rheum. 2013;65(6):1586-91.
108. Wofsy D, Shropshire SM, Hillson JL, Diamond B. Abatacept for lupus nephritis: alternative outcome measures support opposing interpretations of data from a multicenter, randomized, double-blind, placebo-controlled phase II/III study [abstract]. Arthritis & Rheumatology 2011;63(10 Suppl 1):S964-5.
109. An Y, Zhou Y, Bi L, Liu B, Wang H, Lin J, et al. Combined immunosuppressive treatment (CIST) in lupus nephritis: a multicenter, randomized controlled study. Clin Rheumatol. 2019;38:1047-1054.
110. Moroni G, Doria A, Mosca M, Alberighi OD, Ferraccioli G, Todesco S, et al. A randomized pilot trial comparing cyclosporine and azathioprine for maintenance therapy in diffuse lupus nephritis over four years. Clin J Am Soc Nephrol. 2006; 1:925-32.
111. Doria A, Ponticelli C, Mosca M, Ferraccioli GF, Moroni G, Todesco S, et al. A randomized trial comparing cyclosporine versus azathioprine for maintenance therapy in diffuse lupus nephritis [abstract]. Lupus 2004; 7th International Congress on SLE and Related Conditions; 2004 May 9-13; New York (NY). 2004.
112. Moroni G, Doria A, Mosca M, Ferraccioli G, Todesco S, Manno C, et al. A randomized trial comparing cyclosporine versus azathioprine for maintenance therapy in diffuse lupus nephritis [abstract no: F-PO255]. Journal of the American Society of Nephrology 2004; 15(Oct):121A.
113. Moroni G, Doria A, Mosca M, Ferraccioli G, Todesco S, Schena P, et al. A randomized trial comparing cyclosporine versus azathioprine for maintenance therapy in diffuse lupus nephritis [abstract no: MO30]. 41st Congress. European Renal Association. European Dialysis and Transplantation Association; 2004 May 15-18; Lisbon, Portugal. 2004:224.
114. Mosca M, Doria A, Moroni G, Ferrara R, Todesco S, Ponticelli C, et al. Induction therapy with oral cyclophosphamide in lupus nephritis [abstract no: THU0166]. Annual European Congress of Rheumatology EULAR; 2002 June 12-15; Stockholm, Sweden. 2002.
115. Dooley MA, Jayne D, Ginzler EM, Isenberg D, Olsen NJ, Wofsy D, et al. Mycophenolate versus azathioprine as maintenance therapy for lupus nephritis. N Engl J Med. 2011;365: 1886-95.
116. Appel GB, Contreras G, Dooley MA, Ginzler EM, Isenberg D, Jayne D, et al. Mycophenolate mofetil versus cyclophosphamide for induction treatment of lupus nephritis. J Am Soc Nephrol. 2009;20(5):1103-12.
117. Appel GB, Dooley MA, Ginzler EM, Isenberg D, Jayne D, Solomons N, et al. Mycophenolate mofetil compared with intravenous cyclophosphamide as induction therapy for lupus nephritis: Aspreva Lupus Management Study (ALMS) results [abstract no: SA-FC057]. Journal of the American Society of Nephrology 2007;18(Abstracts):47A.
118. Clancy RM, Ginzler EM, Kim M. Association of adiponectin and soluble endothelial protein C receptor (sEPCR) with longitudinal assessments in the induction phase of a randomized multicenter trial comparing mycophenolate mofetil and intravenous cyclophosphamide [abstract no: 263]. ACR/ARHP Annual Scientific Meeting; 2009 Oct 17-21; Philadelphia (PA). 2009.
119. Dall'Era M, Stone D, Levesque V, Cisternas M, Wofsy D. Identification of biomarkers that predict response to treatment of lupus nephritis with mycophenolate mofetil or pulse cyclophosphamide. Arthritis Care Res (Hoboken). 2011;63(3):351-7.
120. Dooley MA, Appel GB, Ginzler EM, Isenberg D, Jayne D, Wofsy D, et al. Aspreva lupus management study (ALMS): maintenance results analysis by racial subgroup [abstract no: OP0168]. Annals of the Rheumatic Diseases 2011;70(Suppl 3):125.
121. Ginzler E. Contribution of vascular well being to the therapeutic response in the induction phase of a randomized multicenter trial comparing MMF to IVC [abstract no: 2063]. ACR/ARHP Annual Scientific Meeting; 2008 Oct 24-29; San Francisco (CA). 2008.
122. Ginzler EM, Appel GB, Dooley MA, Isenberg D, Jayne D, Solomons N, et al. Mycophenolate mofetil and intravenous cyclophosphamide in the Aspreva Lupus Management Study (ALMS): efficacy by racial group [abstract no: L13]. ACR/ARHP Annual Scientific Meeting; 2007 Nov 6-11; Boston (MA). 2007.
123. Ginzler EM, Wofsy D, Isenberg D, Gordon C, Lisk L, Dooley MA; ALMS Group. Nonrenal disease activity following mycophenolate mofetil or intravenous cyclophosphamide as induction treatment for lupus nephritis: findings in a multicenter, prospective, randomized, open-label, parallel-group clinical trial. Arthritis Rheum. 2010;62(1):211-21.
124. Isenberg D. Lessons from the Aspreva Lupus Management Study [abstract no: SP0073]. Annals of the Rheumatic Diseases 2009;68(Suppl 3):23.
125. Isenberg D, Appel GB, Contreras G, Dooley MA, Ginzler EM, Jayne D, et al. Influence of race/ethnicity on response to lupus nephritis treatment: the ALMS study. Rheumatology (Oxford). 2010;49(1):128-40.
126. Isenberg D, Appel GB, Dooley MA, Ginzler EM, Jayne D, Lisk L, et al. Mycophenolate mofetil compared with intravenous cyclophosphamide as induction for lupus nephritis: ALMS results and BILAG responses [abstract no: OP-0018]. Annals of the Rheumatic Diseases 2008;67(Supp II):53.
127. Isenberg D, Appel GB, Dooley MA, Ginzler EM, Jayne D, Solomons N, et al. Mycophenolate mofetil (MMF, Cellcept) for induction and maintenance treatment of lupus nephritis (LN): baseline demographics of patients from the phase III randomized, controlled ASPREVA Lupus Management Study (ALMS) [abstract no: AB0424]. Annals of the Rheumatic Diseases 2007;66(Suppl II):606.
128. Jayne DR, Appel GB, Dooley MA, Ginzler E, Isenberg D, Wofsy D, et al. Results of the Aspreva Lupus Management Study (ALMS) maintenance phase [abstract no: TH-FC111]. Journal of the American Society of Nephrology 2010;21:25A.
129. Nadig RS. Efficacy, toxicity and tolerability of mycophenolate mofetil in patients with lupus nephritis, based on dose/weight ratio [abstract no: 431]. ACR/ARHP Annual Scientific Meeting; 2007 Nov 6-11; Boston (MA). 2007.
130. Schwartz N, Patel T, Ginzler EM, Solomons N, Buyon JP, Clancy RM. Response to MMF therapy for lupus nephritis is independent of genetic variation of inosine monophosphate dehydrogenase [abstract no: 837]. Arthritis & Rheumatology 2012;64(10 Suppl):S365.
131. Schwartz N, Patel T, Ginzler EM, Solomons N, Buyon JP, Clancy RM. Response to MMF therapy for lupus nephritis is independent of genetic variation of inosine monophosphate dehydrogenase [abstract no: 837]. Arthritis & Rheumatology 2012;64(10 Suppl):S365
132. Silva-Fernandez L, Nadig RS, von Gizycki H, Ginzler EM. Efficacy, toxicity and tolerability of mycophenolate mofetil in patients with lupus nephritis, based on dose/weight ratio [abstract no: 431]. ACR/ARHP Annual Scientific Meeting; 2007 Nov 6-11; Boston (MA). 2007.
133. Sinclair A, Appel G, Dooley MA, Ginzler E, Isenberg D, Jayne D, et al. Mycophenolate mofetil as induction and maintenance therapy for lupus nephritis: rationale and protocol for the randomized, controlled Aspreva Lupus Management Study (ALMS). Lupus. 2007;16(12): 972-80.
134. Sinclair A, Appel G, Dooley MA, Ginzler E, Isenberg D, Jayne D, et al. Protocol for the Aspreva Lupus Management Study (ALMS) [abstract no: F-PO882]. Journal of the American Society of Nephrology 2005;16:528A.
135. Stone DH, Dall'Era M, Levesque V, Cisternas MG, Wofsy D. Identification of biomarkers that predict response to treatment of lupus nephritis with mycophenolate mofetil (MMF) or pulse cyclophosphamide (IV) [abstract no: 2073]. ACR/ARHP Annual Scientific Meeting; 2009 Oct 17-21; Philadelphia (PA). 2009.
136. Sundel R, Solomons N, Lisk L; Aspreva Lupus Management Study (ALMS) Group. Efficacy of mycophenolate mofetil in adolescent patients with lupus nephritis: evidence from a two-phase, prospective randomized trial. Lupus. 2012;21(13):1433-43.
137. Sundel RP, Lisk L. Mycophenolate mofetil compared with intravenous cyclophosphamide as induction treatment for pediatric lupus nephritis: a randomized trial [abstract no: 1274]. ACR/ARHP Annual Scientific Meeting; 2008 Oct 24-29; San Francisco, CL. 2008.
138. Walsh M, Solomons N, Lisk L, Jayne DR. Mycophenolate mofetil or intravenous cyclophosphamide for lupus nephritis with poor kidney function: a subgroup analysis of the Aspreva Lupus Management Study. Am J Kidney Dis. 2013;61(5):710-5.
139. Wofsy D, Appel GB, Dooley MA, Ginzler EM, Isenberg D, Jayne D, et al. Aspreva lupus management study maintenance results [abstract no: CS12.6]. Lupus 2010;19(1 Suppl):27.
140. Wofsy D, Appel GB, Dooley MA, Ginzler EM, Isenberg D, Jayne D, et al. Mycophenolate mofetil compared with intravenous cyclophosphamide in the treatment of lupus nephritis: predictors of response [abstract no: SAT0188]. Annals of the Rheumatic Diseases 2008;67(Suppl II):493.
141. Chen W, Liu Q, Chen W, Tang X, Fu P, Liu F, et al. Outcomes of maintenance therapy with tacrolimus versus azathioprine for active lupus nephritis: a multicenter randomized clinical trial. Lupus. 2012;21(9):944-52.
142. Kaballo BG, Ahmed AE, Nur MM, Khalid IO, Abu-Aisha H. Mycophenolate mofetil versus azathioprine for maintenance treatment of lupus nephritis. Saudi J Kidney Dis Transpl. 2016; 27:717-25.
143. Fu Q, Wu C, Dai M, Wang S, Xu J, Dai L, et al. Leflunomide versus azathioprine for maintenance therapy of lupus nephritis: a prospective, multicentre, randomised trial and long-term follow-up. Ann Rheum Dis. 2022;81:1549-1555.
144. Contreras G, Pardo V, Leclercq B, Lenz O, Tozman E, O’Nan P, et al. Sequential therapies for proliferative lupus nephritis. N Engl J Med. 2004;350:971-80.
145. Contreras G, Pardo V, Leclercq B, Gomez E, Reich J, O'Nan P, et al. Maintenance therapy for proliferative forms of lupus nephritis: a randomized clinical trial comparing quarterly intravenous cyclophosphamide (IVCY) versus oral mycophenolate mofetil (MMF) or azathioprine (AZA) [abstract no: F-FC069]. Journal of the American Society of Nephrology 2002;13(Sept Program & Abstracts):15A.
146. Contreras G, Pardo V, Leclercq B, Lenz O, ONan P, Tozman E, et al. Lupus nephritis: sequential therapy with short-term intravenous cyclophosphamide followed by maintenance oral mycophenolate mofetil or oral azathioprine is more efficacious and safer than long-term intravenous cyclophosphamide [abstract no: SA-FC173]. Journal of the American Society of Nephrology 2003;14(Nov):38A.
147. Contreras G, Roth D, Berho M, Perez G, Gomez E, Acosta M, et al. Immunosupressive therapy for proliferative lupus nephritis: preliminary report of a prospective, randomized clinical trial with mycophenolate mofetil (MMF) [abstract]. Journal of the American Society of Nephrology 1999;10(Program &Abstracts):99A.
148. Contreras G, Tozman E, Nahar N, Metz D. Maintenance therapies for proliferative lupus nephritis: mycophenolate mofetil, azathioprine and intravenous cyclophosphamide. Lupus. 2005;14 Suppl 1:s33-8.
149. Houssiau FA, D’Cruz D, Sangle S, Remy P, Vasconcelos C, Petrovic R, et al; MAINTAIN Nephritis Trial Group. Azathioprine versus mycophenolate mofetil for long-term immunosuppression in lupus nephritis: results from the MAINTAIN Nephritis Trial. Ann Rheum Dis. 2010;69:2083-9.
150. Carette S, Klippel JH, Decker JL, Austin HA, Plotz PH, Steinberg AD, et al. Controlled studies of oral immunosuppressive drugs in lupus nephritis. A long-term follow-up. Ann Intern Med. 1983;99:1-8.
151. Chan TM, Li FK, Tang CS, Wong RW, Fang GX, Ji YL, et al. Efficacy of mycophenolate mofetil in patients with diffuse proliferative lupus nephritis. Hong Kong-Guangzhou Nephrology Study Group. N Engl J Med. 2000;343: 1156-62.
152. Chan TM. Mycophenolate mofetil in the treatment of lupus nephritis--7 years on. Lupus. 2008;17(7):617-21.
153. Chan TM, Tse KC, Tang CS, Mok MY, Li FK; Hong Kong Nephrology Study Group. Long-term study of mycophenolate mofetil as continuous induction and maintenance treatment for diffuse proliferative lupus nephritis. J Am Soc Nephrol. 2005;16(4):1076-84.
154. Karassa FB, Isenberg DA. Efficacy of mycophenolate mofetil in patients with diffuse proliferative lupus nephritis. N Engl J Med. 2001;344(5):382-3.
155. Grootscholten C, Ligtenberg G, Hagen EC, van den Wall Bake AW, de Glas-Vos JW, Bijl M, et al; Dutch Working Party on Systemic Lupus Erythematosus. Azathioprine/ methylprednisolone versus cyclophosphamide in proliferative lupus nephritis. A randomized controlled trial. Kidney Int. 2006;70:732-42.
156. Arends S, Grootscholten C, Derksen RH, Berger SP, de Sévaux RG, Voskuyl AE, et al. Long-term follow-up of a randomised controlled trial of azathioprine/methylprednisolone versus cyclophosphamide in patients with proliferative lupus nephritis. Ann Rheum Dis. 2012;71(6):966-73.
157. Grootscholten C, Bajema IM, Florquin S, Steenbergen EJ, Peutz-Kootstra CJ, Goldschmeding R, et al. Interobserver agreement of scoring of histopathological characteristics and classification of lupus nephritis. Nephrol Dial Transplant. 2008;23(1): 223-30.
158. Grootscholten C, Bajema IM, Florquin S, Steenbergen EJ, Peutz-Kootstra CJ, Goldschmeding R, et al. Treatment with cyclophosphamide delays the progression of chronic lesions more effectively than does treatment with azathioprine plus methylprednisolone in patients with proliferative lupus nephritis. Arthritis Rheum. 2007;56(3):924-37.
159. Grootscholten C, Dieker JW, McGrath FD, Roos A, Derksen RH, van der Vlag J, et al. A prospective study of anti-chromatin and anti-C1q autoantibodies in patients with proliferative lupus nephritis treated with cyclophosphamide pulses or azathioprine/ methylprednisolone. Ann Rheum Dis. 2007;66(5):693-6.
160. Grootscholten C, Snoek FJ, Bijl M, van Houwelingen HC, Derksen RH, Berden JH; Dutch Working Party of SLE. Health-related quality of life and treatment burden in patients with proliferative lupus nephritis treated with cyclophosphamide or azathioprine/ methylprednisolone in a randomized controlled trial. J Rheumatol. 2007;34(8):1699-707.
161. Appel GB, Contreras G, Dooley MA, Ginzler EM, Isenberg D, Jayne D, et al. Mycophenolate mofetil versus cyclophosphamide for induction treatment of lupus nephritis. J Am Soc Nephrol. 2009;20:1103-12.
162. Dooley MA, Appel GB, Ginzler EM, Isenberg D, Jayne D, Wofsy D, et al. Aspreva lupus management study (ALMS): maintenance results analysis by racial subgroup [abstract no: OP0168]. Annals of the Rheumatic Diseases 2011;70(Suppl 3):125.
163. Zavada J, Pesickova S, Rysava R, Olejarova M, Horák P, Hrncir Z, et al. Cyclosporine A or intravenous cyclophosphamide for lupus nephritis: the Cyclofa-Lune study. Lupus. 2010; 19: 1281-9.
164. Závada J, Sinikka Pesicková S, Rysavá R, Horák P, Hrncír Z, et al. Extended follow-up of the CYCLOFA-LUNE trial comparing two sequential induction and maintenance treatment regimens for proliferative lupus nephritis based either on cyclophosphamide or on cyclosporine A. Lupus. 2014;23(1):69-74.
165. Mok CC, Ho CT, Siu YP, Chan KW, Kwan TH, Lau CS, et al. Treatment of diffuse proliferative lupus glomerulonephritis: a comparison of two cyclophosphamide- containing regimens. Am J Kidney Dis. 2001;38:256-64.
166. Yee CS, Gordon C, Dostal C, Petera P, Dadoniene J, Griffiths B, et al. EULAR randomised controlled trial of pulse cyclophosphamide and methylprednisolone versus continuous cyclophosphamide and prednisolone followed by azathioprine and prednisolone in lupus nephritis. Ann Rheum Dis. 2004;63:525-9.
167. Furie R, Nicholls K, Cheng TT, Houssiau F, Burgos-Vargas R, Chen SL, et al. Efficacy and safety of abatacept in lupus nephritis: a twelve-month, randomized, double-blind study. Arthritis Rheumatol. 2014;66:379-89.
168. Wofsy D, Hillson JL, Diamond B. Abatacept for lupus nephritis: alternative definitions of complete response support conflicting conclusions. Arthritis Rheum. 2012;64(11):3660-5.
169. Wofsy D, Hillson JL, Diamond B. Comparison of alternative primary outcome measures for use in lupus nephritis clinical trials. Arthritis Rheum. 2013;65(6):1586-91.
170. Furie R, Rovin BH, Houssiau F, Malvar A, Teng YKO, Contreras G, et al. Two-Year, Randomized, Controlled Trial of Belimumab in Lupus Nephritis. N Engl J Med. 2020;383: 1117-1128.
171. Atisha-Fregoso Y, Malkiel S, Harris KM, Byron M, Ding L, Kanaparthi S, et al. Phase II Randomized Trial of Rituximab Plus Cyclophosphamide Followed by Belimumab for the Treatment of Lupus Nephritis. Arthritis Rheumatol. 2021;73: 121-131.
172. Ye F, Wang S, Wang M, Wang H, Guo F, Li G, et al. Clinical analysis of multi-target treatment for complex lupus nephritis. Am J Transl Res. 2022;14:687-692.
