# Supplementary material for: Efficacy and safety of immunosuppressive agents for adults with lupus nephritis: a systematic review and network meta-analysis
Source: Front Immunol. 2023 Oct 13;14:1232244. doi: 10.3389/fimmu.2023.1232244 (PMC10611487; doi:10.3389/fimmu.2023.1232244)
Supplement: Supplementary file 1 [file DataSheet_1.zip › Supplement 4.docx]

Table S1. Quality assessment of included trials

| Study | Random sequence generation | Allocation concealment | Blinding of participants and personnel | Blinding of outcome assessment | Incomplete outcome data | Selective reporting | Other bias |
| --- | --- | --- | --- | --- | --- | --- | --- |
| Steinberg 1971 [1] | Low | Low | Low | Unclear | Low | Low | High |
| Cade 1973 [2] | High | High | High | Unclear | Low | Low | Low |
| Donadio 1974 [3,4] | Low | Unclear | High | Unclear | Unclear | High | Low |
| Hahn 1975 [5] | Low | Low | High | Unclear | Low | High | Low |
| Donadio 1978 [6-8] | Low | Unclear | High | Unclear | Unclear | Low | Low |
| Austin 1986 [9-16] | Low | Unclear | Unclear | Unclear | Low | Low | High |
| Boumpas 1992 [17-19] | Unclear | Low | High | Unclear | Low | Low | Low |
| Sesso 1994 [20,21] | Unclear | Unclear | Unclear | Unclear | Low | Low | High |
| Gourley 1996 [22,23] | Low | Unclear | High | Low | Low | Low | Low |
| Miyasaka 2009 [24] | Unclear | Unclear | Low | Low | Unclear | Low | Low |
| Austin 2009 [25-28] | Unclear | Unclear | High | Unclear | Unclear | Unclear | Unclear |
| Mysler 2013 [29-31] | Unclear | Unclear | Low | Unclear | High | Low | High |
| Hu 2002 [32] | Unclear | Unclear | High | Unclear | Unclear | Unclear | Unclear |
| Ginzler 2005 [33-40] | Low | Low | High | Unclear | Low | High | High |
| Ong 2005 [41,42] | Low | Low | High | Unclear | Low | Low | Low |
| Wang 2007 [43] | Unclear | Unclear | High | Unclear | Unclear | Unclear | Unclear |
| Chen 2007 [44] | Unclear | Unclear | High | Unclear | Unclear | High | Unclear |
| Dyadyk 2007 [45,46] | Unclear | Unclear | High | Unclear | Unclear | High | Low |
| Mulic-Bacic 2008 [47] | Unclear | Unclear | High | Unclear | Low | High | Unclear |
| El-Shafey 2010 [48] | Unclear | Unclear | High | Unclear | Unclear | Low | Low |
| Chen 2011 [49,50] | Low | Low | High | Unclear | Low | Low | Low |
| Yap 2012 [51] | Unclear | Unclear | High | Unclear | Unclear | Unclear | Unclear |
| Rathi 2016 [52-55] | Unclear | Unclear | High | Unclear | Unclear | Low | High |
| Mok 2016 [56-66] | Low | Unclear | High | Unclear | Low | Low | Low |
| Mendonca 2017 [67] | Unclear | Unclear | High | Unclear | Low | High | Low |
| Sedhain 2018 [68,69] | Unclear | Unclear | High | Unclear | Unclear | Unclear | High |
| Kamanamool 2018 [70-72] | Low | Low | High | Unclear | Low | Low | Low |
| Zhang 2019 [73] | Unclear | Unclear | High | Unclear | Low | Low | Unclear |
| Zheng 2022 [74] | Low | Unclear | High | Unclear | Low | Low | Low |
| Li 2022 [75] | Unclear | Unclear | High | Unclear | Low | Unclear | Low |
| Li 2012 [76,77] | Unclear | Unclear | High | Unclear | Low | Low | Low |
| Feng 2014 [78] | Low | Unclear | High | Unclear | Unclear | High | Unclear |
| Bao 2008 [79,80] | Low | Unclear | High | Low | Low | Low | Low |
| Li 2009 [81,82] | Low | Low | High | Unclear | Low | High | High |
| Rovin 2012 [83-94] | Unclear | Unclear | Low | Unclear | Low | Low | High |
| Liu 2015 [95] | Low | Unclear | Low | Unclear | High | High | Low |
| Sun 2015 [96] | Unclear | Unclear | High | Unclear | Low | Unclear | High |
| Rovin 2019 [97] | Low | Unclear | Low | Low | Low | Low | Low |
| Zhang 2020 [98] | Low | Unclear | High | Unclear | Low | Unclear | Unclear |
| Rovin 2021 [99] | Low | Low | Low | Low | Low | Low | Low |
| Furie 2022 [100] | Low | Unclear | Unclear | Low | Low | Low | Low |
| Jayne 2022 [101] | Low | Unclear | Unclear | Low | Low | Low | Low |
| Askanase 2014 [102-108] | Unclear | Unclear | Low | Unclear | Low | Low | Low |
| An 2019 [109] | Unclear | Unclear | High | Unclear | Low | Low | Unclear |
| Moroni 2006 [110-114] | Low | Low | High | Low | Low | Low | High |
| Dooley 2011 [115-140] | Unclear | Low | High | Low | Low | Low | High |
| Chen 2012 [141] | Low | Low | High | Unclear | Low | Low | Low |
| Kaballo 2016 [142] | Low | Unclear | Unclear | Unclear | Low | Low | Low |
| Fu 2020 [143] | Low | Low | Unclear | Unclear | Low | Low | Unclear |
| Contreras 2004 [144-148] | High | Low | High | Unclear | Low | Low | High |
| Houssiau 2010 [149] | Unclear | Unclear | High | Unclear | Low | Low | Unclear |
| Carette 1983 [150] | Low | Unclear | Unclear | Unclear | Low | Low | High |
| Chan 2000 [151-154] | Low | Unclear | Unclear | Low | Low | Low | Low |
| Grootscholten 2006 [155-160] | Low | Unclear | High | Unclear | Low | Low | Low |
| Appel 2009 [161,162] | Unclear | Low | High | Low | Low | Low | High |
| Zavada 2010 [163,164] | Unclear | Low | High | Unclear | Low | Low | Low |
| Mok 2001 [165] | Unclear | Unclear | High | Unclear | Unclear | Unclear | Unclear |
| Yee 2004 [166] | Low | Unclear | High | Unclear | Low | High | High |
| Furie 2014 [167-169] | Unclear | Unclear | Low | Unclear | High | Low | High |
| Furie 2020 [170] | Low | Low | Low | Low | Low | Low | Low |
| Atisha-Fregoso 2021 [171] | Low | Unclear | High | Unclear | Low | Unclear | Unclear |
| Ye 2022 [172] | Unclear | Unclear | High | Unclear | Low | Unclear | Unclear |

1. **Steinberg 1971**

| Bias | Authors’ judgement | Support of judgement |
| --- | --- | --- |
| Random sequence generation (selection bias) | Low risk | Used consecutively numbered envelopes, each containing a randomly assigned prescription for placebo or CPA |
| Allocation concealment (selection bias) | Low risk | As each patient entered the study, the next sequential envelope was opened in the pharmacy |
| Blinding of participants and personnel (performance bias) | Low risk | Double-blind study |
| Blinding of outcome assessment (detection bias) | Unclear risk | Insufficient information to permit judgement |
| Incomplete outcome data (attrition bias) | Low risk | No missing outcome data |
| Selective reporting (reporting bias) | Low risk | Study protocol available and pre-specified outcomes were reported |
| Other bias | High risk | Cross-over of two participants from the placebo to CPA arm were included in the analysis |

1. **Cade 1973**

| Bias | Authors’ judgement | Support of judgement |
| --- | --- | --- |
| Random sequence generation (selection bias) | High risk | Chronological appearance |
| Allocation concealment (selection bias) | High risk | Assigned in alternate fashion by division secretary |
| Blinding of participants and personnel (performance bias) | High risk | Open-label study |
| Blinding of outcome assessment (detection bias) | Unclear risk | Insufficient information to permit judgement |
| Incomplete outcome data (attrition bias) | Low risk | Insufficient information to permit judgement |
| Selective reporting (reporting bias) | Low risk | All pre-specified outcomes are reported |
| Other bias | Low risk | The study appears to be free of other sources of bias |

1. **Donadio 1974**

| Bias | Authors’ judgement | Support of judgement |
| --- | --- | --- |
| Random sequence generation (selection bias) | Low risk | Participants allocated within each category to treatment group A or B according to random selection. Table of random numbers used. Each incoming set of 4 participants assigned to 2 As and 2 Bs in random order |
| Allocation concealment (selection bias) | Unclear risk | Insufficient information to permit judgement |
| Blinding of participants and personnel (performance bias) | High risk | Open-label study |
| Blinding of outcome assessment (detection bias) | Unclear risk | Insufficient information to permit judgement |
| Incomplete outcome data (attrition bias) | Unclear risk | Insufficient information to permit judgement |
| Selective reporting (reporting bias) | High risk | One or more reported primary outcomes were not pre-specified |
| Other bias | Low risk | The study appears to be free of other sources of bias |

1. **Hahn 1975**

| Bias | Authors’ judgement | Support of judgement |
| --- | --- | --- |
| Random sequence generation (selection bias) | Low risk | Slips of paper bearing letters A or B sealed in envelopes then placed in a drawer. On randomising patient, envelopes drawn randomly from drawer |
| Allocation concealment (selection bias) | Low risk | Sealed envelopes used in randomisation |
| Blinding of participants and personnel (performance bias) | High risk | Open-label study |
| Blinding of outcome assessment (detection bias) | Unclear risk | Insufficient information to permit judgement |
| Incomplete outcome data (attrition bias) | Low risk | No missing outcome data |
| Selective reporting (reporting bias) | High risk | Not all expected clinical outcomes were reported |
| Other bias | Low risk | The study appears to be free of other sources of bias |

1. **Donadio 1978**

| Bias | Authors’ judgement | Support of judgement |
| --- | --- | --- |
| Random sequence generation (selection bias) | Low risk | Random number tables used |
| Allocation concealment (selection bias) | Unclear risk | Insufficient information to permit judgement |
| Blinding of participants and personnel (performance bias) | High risk | Open-label study |
| Blinding of outcome assessment (detection bias) | Unclear risk | Insufficient information to permit judgement |
| Incomplete outcome data (attrition bias) | Unclear risk | Insufficient information to permit judgement |
| Selective reporting (reporting bias) | Low risk | All expected outcomes are reported |
| Other bias | Low risk | The study appears to be free of other sources of bias |

1. **Boumpas 1992**

| Bias | Authors’ judgement | Support of judgement |
| --- | --- | --- |
| Random sequence generation (selection bias) | Unclear risk | Patients were assigned randomly to one of three treatment groups. No further details on randomisation |
| Allocation concealment (selection bias) | Low risk | Allocation drawn from a set of masked cards |
| Blinding of participants and personnel (performance bias) | High risk | Open-label study |
| Blinding of outcome assessment (detection bias) | Unclear risk | Insufficient information to permit judgement |
| Incomplete outcome data (attrition bias) | Low risk | No missing outcome data |
| Selective reporting (reporting bias) | Low risk | Study protocol available and pre-specified outcomes were reported |
| Other bias | Low risk | The study appears to be free of other sources of bias |

1. **Sesso 1994**

| Bias | Authors’ judgement | Support of judgement |
| --- | --- | --- |
| Random sequence generation (selection bias) | Unclear risk | Study was described as randomised, method of randomisation was not reported |
| Allocation concealment (selection bias) | Unclear risk | Insufficient information to permit judgement |
| Blinding of participants and personnel (performance bias) | Unclear risk | Insufficient information to permit judgement |
| Blinding of outcome assessment (detection bias) | Unclear risk | Insufficient information to permit judgement |
| Incomplete outcome data (attrition bias) | Low risk | No missing outcome data |
| Selective reporting (reporting bias) | Low risk | All expected outcomes were reported |
| Other bias | High risk | Proteinuria between groups at baseline was different |

1. **Gourley 1996**

| Bias | Authors’ judgement | Support of judgement |
| --- | --- | --- |
| Random sequence generation (selection bias) | Low risk | Masked cards from table of random numbers |
| Allocation concealment (selection bias) | Unclear risk | Using masked card but no description methods of allocation concealment |
| Blinding of participants and personnel (performance bias) | High risk | Open-label study |
| Blinding of outcome assessment (detection bias) | Low risk | Outcome data with the exception of adverse events, were collected in a blinded manner |
| Incomplete outcome data (attrition bias) | Low risk | No missing outcome data; participants at endpoints censored but considered in final analysis |
| Selective reporting (reporting bias) | Low risk | Study protocol available and prespecified outcomes were reported |
| Other bias | Low risk | The study appears to be free of other sources of bias |

1. **Miyasaka 2009**

| Bias | Authors’ judgement | Support of judgement |
| --- | --- | --- |
| Random sequence generation (selection bias) | Unclear risk | Study was described as randomised, method of randomisation was not reported |
| Allocation concealment (selection bias) | Unclear risk | Insufficient information to permit judgement |
| Blinding of participants and personnel (performance bias) | Low risk | Double-blind study |
| Blinding of outcome assessment (detection bias) | Low risk | Outcome data with the exception of adverse events, were collected in a blinded manner |
| Incomplete outcome data (attrition bias) | Unclear risk | Insufficient information to permit judgement |
| Selective reporting (reporting bias) | Low risk | Study protocol available and prespecified outcomes were reported |
| Other bias | Low risk | The study appears to be free of other sources of bias |

1. **Austin 2009**

| Bias | Authors’ judgement | Support of judgement |
| --- | --- | --- |
| Random sequence generation (selection bias) | Unclear risk | Study was described as randomised, method of randomisation was not reported |
| Allocation concealment (selection bias) | Unclear risk | Insufficient information to permit judgement |
| Blinding of participants and personnel (performance bias) | High risk | Open-label study |
| Blinding of outcome assessment (detection bias) | Unclear risk | Insufficient information to permit judgement |
| Incomplete outcome data (attrition bias) | Unclear risk | Insufficient information to permit judgement |
| Selective reporting (reporting bias) | Unclear risk | Insufficient information to permit judgement |
| Other bias | Unclear risk | Insufficient information to permit judgement |

1. **Mysler 2013**

| Bias | Authors’ judgement | Support of judgement |
| --- | --- | --- |
| Random sequence generation (selection bias) | Unclear risk | Study was described as randomised, method of randomisation was not reported |
| Allocation concealment (selection bias) | Unclear risk | Insufficient information to permit judgement |
| Blinding of participants and personnel (performance bias) | Low risk | Double-blind, placebo-controlled study |
| Blinding of outcome assessment (detection bias) | Unclear risk | Insufficient information to permit judgement |
| Incomplete outcome data (attrition bias) | High risk | Study was terminated before completion. Only 36.8% of patients completed the 48- week treatment period and were included in the analysis |
| Selective reporting (reporting bias) | Low risk | Study protocol available and pre-specified outcomes were reported |
| Other bias | High risk | Genentech and Hoffman-La Roche funded the study and were involved in study design; Conflict of interest of authors relating to the pharmaceutical companies that funded the study; High drop-out rates (around 52%) with the early termination of the study; The 1000 mg ocrelizumabtreated group had slightly higher proportion of Caucasian patients and a lower proportionof Asianpatients thanthe other two groups |

1. **Hu 2002**

| Bias | Authors’ judgement | Support of judgement |
| --- | --- | --- |
| Random sequence generation (selection bias) | Unclear risk | Insufficient information to permit judgement |
| Allocation concealment (selection bias) | Unclear risk | Insufficient information to permit judgement |
| Blinding of participants and personnel (performance bias) | High risk | Open-label study |
| Blinding of outcome assessment (detection bias) | Unclear risk | Insufficient information to permit judgement |
| Incomplete outcome data (attrition bias) | Unclear risk | Insufficient information to permit judgement |
| Selective reporting (reporting bias) | Unclear risk | Insufficient information to permit judgement |
| Other bias | Unclear risk | Insufficient information to permit judgement |

1. **Ginzler 2005**

| Bias | Authors’ judgement | Support of judgement |
| --- | --- | --- |
| Random sequence generation (selection bias) | Low risk | Treatment assigned at central site with the use of sealed envelopes |
| Allocation concealment (selection bias) | Low risk | Sealed envelopes used |
| Blinding of participants and personnel (performance bias) | High risk | Open-label study |
| Blinding of outcome assessment (detection bias) | Unclear risk | Insufficient information to permit judgement |
| Incomplete outcome data (attrition bias) | Low risk | No missing outcome data |
| Selective reporting (reporting bias) | High risk | Due to early termination, primary outcome as per protocol not reported; Not all expected outcomes reported |
| Other bias | High risk | The study was terminated early and there was heavy cross-over between study arms. Funding provided by a supplemental grant from Roche laboratories |

1. **Ong 2005**

| Bias | Authors’ judgement | Support of judgement |
| --- | --- | --- |
| Random sequence generation (selection bias) | Low risk | Randomisation code generated separately for each centre using random permutated block method with randomly varyingblock size (1:1) |
| Allocation concealment (selection bias) | Low risk | Randomisation performed centrally |
| Blinding of participants and personnel (performance bias) | High risk | Open-label study |
| Blinding of outcome assessment (detection bias) | Unclear risk | Insufficient information to permit judgement |
| Incomplete outcome data (attrition bias) | Low risk | No missing outcome data |
| Selective reporting (reporting bias) | Low risk | Study protocol available and pre-specified outcomes were reported |
| Other bias | Low risk | The study appears to be free of other sources of bias |

1. **Wang 2007**

| Bias | Authors’ judgement | Support of judgement |
| --- | --- | --- |
| Random sequence generation (selection bias) | Unclear risk | Insufficient information to permit judgement |
| Allocation concealment (selection bias) | Unclear risk | Insufficient information to permit judgement |
| Blinding of participants and personnel (performance bias) | High risk | Open-label study |
| Blinding of outcome assessment (detection bias) | Unclear risk | Insufficient information to permit judgement |
| Incomplete outcome data (attrition bias) | Unclear risk | Insufficient information to permit judgement |
| Selective reporting (reporting bias) | Unclear risk | Insufficient information to permit judgement |
| Other bias | Unclear risk | Insufficient information to permit judgement |

1. **Chen 2007**

| Bias | Authors’ judgement | Support of judgement |
| --- | --- | --- |
| Random sequence generation (selection bias) | Unclear risk | Insufficient information to permit judgement |
| Allocation concealment (selection bias) | Unclear risk | Insufficient information to permit judgement |
| Blinding of participants and personnel (performance bias) | High risk | Open-label study |
| Blinding of outcome assessment (detection bias) | Unclear risk | Insufficient information to permit judgement |
| Incomplete outcome data (attrition bias) | Unclear risk | Insufficient information to permit judgement |
| Selective reporting (reporting bias) | High risk | Not all expected outcomes reported |
| Other bias | Unclear risk | Insufficient information to permit judgement |

1. **Dyadyk 2007**

| Bias | Authors’ judgement | Support of judgement |
| --- | --- | --- |
| Random sequence generation (selection bias) | Unclear risk | Study was described as randomised, method of randomisation was not reported |
| Allocation concealment (selection bias) | Unclear risk | Insufficient information to permit judgement |
| Blinding of participants and personnel (performance bias) | High risk | Open-label study |
| Blinding of outcome assessment (detection bias) | Unclear risk | Insufficient information to permit judgement |
| Incomplete outcome data (attrition bias) | Unclear risk | Insufficient information to permit judgement |
| Selective reporting (reporting bias) | High risk | Not all expected outcomes reported |
| Other bias | Low risk | The study appears to be free of other sources of bias |

1. **Mulic-Bacic 2008**

| Bias | Authors’ judgement | Support of judgement |
| --- | --- | --- |
| Random sequence generation (selection bias) | Unclear risk | Study was described as randomised, method of randomisation was not reported |
| Allocation concealment (selection bias) | Unclear risk | Insufficient information to permit judgement |
| Blinding of participants and personnel (performance bias) | High risk | Open-label study |
| Blinding of outcome assessment (detection bias) | Unclear risk | Insufficient information to permit judgement |
| Incomplete outcome data (attrition bias) | Low risk | No missing outcome data |
| Selective reporting (reporting bias) | High risk | Not all expected clinical outcomes reported and no protocol available; abstract-only publication |
| Other bias | Unclear risk | Abstract-only publication; insufficient information to permit judgement |

1. **El-Shafey 2010**

| Bias | Authors’ judgement | Support of judgement |
| --- | --- | --- |
| Random sequence generation (selection bias) | Unclear risk | Study was described as randomised, method of randomisation was not reported |
| Allocation concealment (selection bias) | Unclear risk | Insufficient information to permit judgement |
| Blinding of participants and personnel (performance bias) | High risk | Open-label study |
| Blinding of outcome assessment (detection bias) | Unclear risk | Insufficient information to permit judgement |
| Incomplete outcome data (attrition bias) | Unclear risk | Insufficient information to permit judgement |
| Selective reporting (reporting bias) | Low risk | Study protocol available and pre-specified outcomes were reported |
| Other bias | Low risk | The study appears to be free of other sources of bias |

1. **Chen 2011**

| Bias | Authors’ judgement | Support of judgement |
| --- | --- | --- |
| Random sequence generation (selection bias) | Low risk | Randomisation was conducted at a central office using a computer-based random allocation sequence table; randomisation not stratified by centre or baseline characteristic |
| Allocation concealment (selection bias) | Low risk | Allocation concealment performed by enclosing assignments in sequentially numbered, opaque, closed envelopes |
| Blinding of participants and personnel (performance bias) | High risk | Open-label study |
| Blinding of outcome assessment (detection bias) | Unclear risk | Insufficient information to permit judgement |
| Incomplete outcome data (attrition bias) | Low risk | The primary outcome (complete remission) and secondary outcomes partial remission and treatment failure were reported on an intention to treat bases. |
| Selective reporting (reporting bias) | Low risk | Study protocol available and pre-specified outcomes were reported |
| Other bias | Low risk | Astellas Pharmaceutics supplied TAC but had no role in the design or conduct of the study or analysis or interpretation of results |

1. **Yap 2012**

| Bias | Authors’ judgement | Support of judgement |
| --- | --- | --- |
| Random sequence generation (selection bias) | Unclear risk | Study was described as randomised, method of randomisation was not reported |
| Allocation concealment (selection bias) | Unclear risk | Insufficient information to permit judgement |
| Blinding of participants and personnel (performance bias) | High risk | Open-label study |
| Blinding of outcome assessment (detection bias) | Unclear risk | Insufficient information to permit judgement |
| Incomplete outcome data (attrition bias) | Unclear risk | Insufficient information to permit judgement |
| Selective reporting (reporting bias) | Unclear risk | Insufficient information to permit judgement |
| Other bias | Unclear risk | Insufficient information to permit judgement |

1. **Rathi 2016**

| Bias | Authors’ judgement | Support of judgement |
| --- | --- | --- |
| Random sequence generation (selection bias) | Unclear risk | Study was described as randomised, method of randomisation was not reported |
| Allocation concealment (selection bias) | Unclear risk | Insufficient information to permit judgement |
| Blinding of participants and personnel (performance bias) | High risk | Open-label study |
| Blinding of outcome assessment (detection bias) | Unclear risk | Insufficient information to permit judgement |
| Incomplete outcome data (attrition bias) | Unclear risk | Insufficient information to permit judgement |
| Selective reporting (reporting bias) | Low risk | Study protocol available from Indian clinical trials registry and pre-specified outcomes were reported |
| Other bias | High risk | High dropout rate; baseline characteristics different between the two groups with UPCR significantly higher in the CPA group |

1. **Mok 2016**

| Bias | Authors’ judgement | Support of judgement |
| --- | --- | --- |
| Random sequence generation (selection bias) | Low risk | Participants were randomised by computer-generated blocks of four in a 1:1 ratio |
| Allocation concealment (selection bias) | Unclear risk | Central research assistant was responsible for treatment allocation |
| Blinding of participants and personnel (performance bias) | High risk | Open-label study |
| Blinding of outcome assessment (detection bias) | Unclear risk | Insufficient information to permit judgement |
| Incomplete outcome data (attrition bias) | Low risk | No missing outcome data |
| Selective reporting (reporting bias) | Low risk | Study protocol available and pre-specified outcomes were reported |
| Other bias | Low risk | The study appears to be free of other sources of bias |

1. **Mendonca 2017**

| Bias | Authors’ judgement | Support of judgement |
| --- | --- | --- |
| Random sequence generation (selection bias) | Unclear risk | Study was described as randomised, method of randomisation was not reported |
| Allocation concealment (selection bias) | Unclear risk | Insufficient information to permit judgement |
| Blinding of participants and personnel (performance bias) | High risk | Open-label study |
| Blinding of outcome assessment (detection bias) | Unclear risk | Insufficient information to permit judgement |
| Incomplete outcome data (attrition bias) | Low risk | No missing outcome data |
| Selective reporting (reporting bias) | High risk | No protocol available, some expected outcomes not reported |
| Other bias | Low risk | The study appears to be free of other sources of bias |

1. **Sedhain 2018**

| Bias | Authors’ judgement | Support of judgement |
| --- | --- | --- |
| Random sequence generation (selection bias) | Unclear risk | Study was described as randomised, method of randomisation was not reported |
| Allocation concealment (selection bias) | Unclear risk | Insufficient information to permit judgement |
| Blinding of participants and personnel (performance bias) | High risk | Open-label study |
| Blinding of outcome assessment (detection bias) | Unclear risk | Insufficient information to permit judgement |
| Incomplete outcome data (attrition bias) | Unclear risk | Insufficient information to permit judgement |
| Selective reporting (reporting bias) | Unclear risk | Insufficient information to permit judgement |
| Other bias | High risk | Characteristics of the six patients unable to complete the study period are not provided and these patients were not included in the analysis; abstract-only publication |

1. **Kamanamool 2018**

| Bias | Authors’ judgement | Support of judgement |
| --- | --- | --- |
| Random sequence generation (selection bias) | Low risk | We stratified patients into two strata according to the classification of renal pathology (Class III-IV LN or Class V III/IV LN). Patients were randomly assigned 1:1 to a TAC group or an MMF group. |
| Allocation concealment (selection bias) | Low risk | To preserve the allocation concealment, the generation of blocks of four to six randomisation lists was electronically produced at Ramathibodi Hospital and web-based randomizations was used |
| Blinding of participants and personnel (performance bias) | High risk | Open-label study |
| Blinding of outcome assessment (detection bias) | Unclear risk | Insufficient information to permit judgement |
| Incomplete outcome data (attrition bias) | Low risk | No missing outcome data |
| Selective reporting (reporting bias) | Low risk | Study protocol available and pre-specified outcomes were reported |
| Other bias | Low risk | Astellas Pharma (Thailand) Co., Ltd. provided study drug and funded the study but had no role in study design, data collection, data analysis, data interpretation or conclusions. The study appears to be free of other sources of bias |

1. **Zhang 2019**

| Bias | Authors’ judgement | Support of judgement |
| --- | --- | --- |
| Random sequence generation (selection bias) | Unclear risk | Study was described as randomised, method of randomisation was not reported |
| Allocation concealment (selection bias) | Unclear risk | Insufficient information to permit judgement |
| Blinding of participants and personnel (performance bias) | High risk | Open-label study |
| Blinding of outcome assessment (detection bias) | Unclear risk | Insufficient information to permit judgement |
| Incomplete outcome data (attrition bias) | Low risk | No missing outcome data |
| Selective reporting (reporting bias) | Low risk | Study protocol available and pre-specified outcomes were reported |
| Other bias | Unclear risk | Insufficient information to permit judgement |

1. **Zheng 2022**

| Bias | Authors’ judgement | Support of judgement |
| --- | --- | --- |
| Random sequence generation (selection bias) | Low risk | Randomized (1:1) via a centralized randomization system using interactive response technology |
| Allocation concealment (selection bias) | Unclear risk | Insufficient information to permit judgement |
| Blinding of participants and personnel (performance bias) | High risk | Open-label study |
| Blinding of outcome assessment (detection bias) | Unclear risk | Insufficient information to permit judgement |
| Incomplete outcome data (attrition bias) | Low risk | No missing outcome data |
| Selective reporting (reporting bias) | Low risk | Study protocol available and pre-specified outcomes were reported |
| Other bias | Low risk | The study appears to be free of other sources of bias |

1. **Li 2022**

| Bias | Authors’ judgement | Support of judgement |
| --- | --- | --- |
| Random sequence generation (selection bias) | Unclear risk | Study was described as randomised, method of randomisation was not reported |
| Allocation concealment (selection bias) | Unclear risk | Insufficient information to permit judgement |
| Blinding of participants and personnel (performance bias) | High risk | Open-label study |
| Blinding of outcome assessment (detection bias) | Unclear risk | Insufficient information to permit judgement |
| Incomplete outcome data (attrition bias) | Low risk | No missing outcome data |
| Selective reporting (reporting bias) | Unclear risk | Insufficient information to permit judgement |
| Other bias | Low risk | The study appears to be free of other sources of bias |

1. **Li 2012**

| Bias | Authors’ judgement | Support of judgement |
| --- | --- | --- |
| Random sequence generation (selection bias) | Unclear risk | Study was described as randomised, method of randomisation was not reported |
| Allocation concealment (selection bias) | Unclear risk | Insufficient information to permit judgement |
| Blinding of participants and personnel (performance bias) | High risk | Open-label study |
| Blinding of outcome assessment (detection bias) | Unclear risk | Insufficient information to permit judgement |
| Incomplete outcome data (attrition bias) | Low risk | No missing outcome data |
| Selective reporting (reporting bias) | Low risk | Study protocol available and pre-specified outcomes were reported |
| Other bias | Low risk | The study appears to be free of other sources of bias |

1. **Feng 2014**

| Bias | Authors’ judgement | Support of judgement |
| --- | --- | --- |
| Random sequence generation (selection bias) | Low risk | At 1:1:1 ratio by computer block randomization |
| Allocation concealment (selection bias) | Unclear risk | Insufficient information to permit judgement |
| Blinding of participants and personnel (performance bias) | High risk | Open-label study |
| Blinding of outcome assessment (detection bias) | Unclear risk | Insufficient information to permit judgement |
| Incomplete outcome data (attrition bias) | Unclear risk | Insufficient information to permit judgement |
| Selective reporting (reporting bias) | High risk | One or more reported primary outcomes were not pre-specified |
| Other bias | Unclear risk | Insufficient information to permit judgement |

1. **Austin 1986**

| Bias | Authors’ judgement | Support of judgement |
| --- | --- | --- |
| Random sequence generation (selection bias) | Low risk | Drawing marked card sequence from a table of random numbers |
| Allocation concealment (selection bias) | Unclear risk | Insufficient information to permit judgement |
| Blinding of participants and personnel (performance bias) | Unclear risk | Insufficient information to permit judgement |
| Blinding of outcome assessment (detection bias) | Unclear risk | Insufficient information to permit judgement |
| Incomplete outcome data (attrition bias) | Low risk | 3.6% (4/111) of participants excluded as they did not complete 3 months of treatment |
| Selective reporting (reporting bias) | Low risk | All expected outcomes were reported |
| Other bias | High risk | Patients were assigned to treatment groups 1, 2 and 3 from the beginning of the study (1969). Treatment groups 4 and 5 were introduced in January 1973. Pooling of multiple studies |

1. **Bao 2008**

| Bias | Authors’ judgement | Support of judgement |
| --- | --- | --- |
| Random sequence generation (selection bias) | Low risk | A computer-generated randomisation list was drawn up by a statistician with a block of every four participants. They enrolled participants were allocated the next available number upon entry into the study |
| Allocation concealment (selection bias) | Unclear risk | A computer-generated randomisation list was given to the pharmacy department. Each patient collected medication directly from the pharmacy department. Unclear whether participants and or investigators might have an opportunity to influence assignment |
| Blinding of participants and personnel (performance bias) | High risk | Open-label study |
| Blinding of outcome assessment (detection bias) | Low risk | Adjudication of primary and key secondary outcome judged at coordinating centre by personnel who had no knowledge of the treatment assignment and ratings were confirmed by repeat testing after a 1 month interval |
| Incomplete outcome data (attrition bias) | Low risk | No missing outcome data |
| Selective reporting (reporting bias) | Low risk | All expected outcomes were reported |
| Other bias | Low risk | Supported by Roche China and Astellas Ireland. Co. Ltd. Partially supported but no role in design, study or analysis |

1. **Li 2009**

| Bias | Authors’ judgement | Support of judgement |
| --- | --- | --- |
| Random sequence generation (selection bias) | Low risk | Randomisation according to a randomisation table kept by a third party |
| Allocation concealment (selection bias) | Low risk | Randomisation table kept by a third party |
| Blinding of participants and personnel (performance bias) | High risk | Open-label study |
| Blinding of outcome assessment (detection bias) | Unclear risk | Insufficient information to permit judgement |
| Incomplete outcome data (attrition bias) | Low risk | No missing outcome data |
| Selective reporting (reporting bias) | High risk | Not all expected outcomes were reported |
| Other bias | High risk | Roche provided study drug but had no role in study design, data collection, data analysis, data interpretation or writing of the report. The study appears to be free of other sources of bias |

1. **Rovin 2012**

| Bias | Authors’ judgement | Support of judgement |
| --- | --- | --- |
| Random sequence generation (selection bias) | Unclear risk | Study was described as randomised, method of randomisation was not reported |
| Allocation concealment (selection bias) | Unclear risk | Insufficient information to permit judgement |
| Blinding of participants and personnel (performance bias) | Low risk | Double-blind, double-dummy placebo study |
| Blinding of outcome assessment (detection bias) | Unclear risk | Insufficient information to permit judgement |
| Incomplete outcome data (attrition bias) | Low risk | No missing outcome data |
| Selective reporting (reporting bias) | Low risk | Study protocol available and pre-specified outcomes were reported |
| Other bias | High risk | Some authors declared grants/research support from Genentech and Aspreva, and sponsor included in data analysis and authorship |

1. **Liu 2015**

| Bias | Authors’ judgement | Support of judgement |
| --- | --- | --- |
| Random sequence generation (selection bias) | Low risk | Study was described as randomised, method of randomisation was not reported |
| Allocation concealment (selection bias) | Unclear risk | Insufficient information to permit judgement |
| Blinding of participants and personnel (performance bias) | Low risk | Double-blind, double-dummy placebo study |
| Blinding of outcome assessment (detection bias) | Unclear risk | Insufficient information to permit judgement |
| Incomplete outcome data (attrition bias) | High risk | No missing outcome data |
| Selective reporting (reporting bias) | High risk | Study protocol available and pre-specified outcomes were reported |
| Other bias | Low risk | Some authors declared grants/research support from Genentech and Aspreva, and sponsor included in data analysis and authorship |

1. **Rovin 2019**

| Bias | Authors’ judgement | Support of judgement |
| --- | --- | --- |
| Random sequence generation (selection bias) | Low risk | There was a randomization block size of 6, and kit numbers were a 5-digit number. Randomized patients were assigned the next available sequential kit number globally via a centralized Interactive Web Response System. |
| Allocation concealment (selection bias) | Unclear risk | Insufficient information to permit judgement |
| Blinding of participants and personnel (performance bias) | Low risk | Double-blind, double-dummy placebo study |
| Blinding of outcome assessment (detection bias) | Low risk | All study personnel and subjects were blind to the study drug administered using a double-blind method |
| Incomplete outcome data (attrition bias) | Low risk | No missing outcome data |
| Selective reporting (reporting bias) | Low risk | Study protocol available and pre-specified outcomes were reported |
| Other bias | Low risk | The study appears to be free of other sources of bias |

1. **Rovin 2021**

| Bias | Authors’ judgement | Support of judgement |
| --- | --- | --- |
| Random sequence generation (selection bias) | Low risk | An interactive web response system was used to randomly assign patients (1:1) |
| Allocation concealment (selection bias) | Low risk | The randomisation schema (stratification) was generated by a masked statistician not involved in the study |
| Blinding of participants and personnel (performance bias) | Low risk | Double-blind study |
| Blinding of outcome assessment (detection bias) | Low risk | Patients, investigators, and the sponsor remained masked to the randomisation assignment for the duration of the study |
| Incomplete outcome data (attrition bias) | Low risk | No missing outcome data |
| Selective reporting (reporting bias) | Low risk | Study protocol available and pre-specified outcomes were reported |
| Other bias | Low risk | The study appears to be free of other sources of bias |

1. **Askanase 2014**

| Bias | Authors’ judgement | Support of judgement |
| --- | --- | --- |
| Random sequence generation (selection bias) | Unclear risk | Study was described as randomised, method of randomisation was not reported |
| Allocation concealment (selection bias) | Unclear risk | Insufficient information to permit judgement |
| Blinding of participants and personnel (performance bias) | Low risk | Double-blind study |
| Blinding of outcome assessment (detection bias) | Unclear risk | Insufficient information to permit judgement |
| Incomplete outcome data (attrition bias) | Low risk | No missing outcome data |
| Selective reporting (reporting bias) | Low risk | Study protocol available and pre-specified outcomes were reported |
| Other bias | Low risk | The study appears to be free of other sources of bias |

1. **An 2019**

| Bias | Authors’ judgement | Support of judgement |
| --- | --- | --- |
| Random sequence generation (selection bias) | Unclear risk | Study was described as randomised, method of randomisation was not reported |
| Allocation concealment (selection bias) | Unclear risk | Insufficient information to permit judgement |
| Blinding of participants and personnel (performance bias) | High risk | Open-label study |
| Blinding of outcome assessment (detection bias) | Unclear risk | Insufficient information to permit judgement |
| Incomplete outcome data (attrition bias) | Low risk | No missing outcome data |
| Selective reporting (reporting bias) | Low risk | Study protocol available and pre-specified outcomes were reported |
| Other bias | Unclear risk | Insufficient information to permit judgement |

1. **Moroni 2006**

| Bias | Authors’ judgement | Support of judgement |
| --- | --- | --- |
| Random sequence generation (selection bias) | Low risk | Randomisation according to a coin-based design |
| Allocation concealment (selection bias) | Low risk | Stratified by centre and performed centrally. Phone calls to randomisation centrecomputer program assigned participants |
| Blinding of participants and personnel (performance bias) | High risk | Open-label study |
| Blinding of outcome assessment (detection bias) | Low risk | Blinded endpoint study |
| Incomplete outcome data (attrition bias) | Low risk | No missing outcome data |
| Selective reporting (reporting bias) | Low risk | Study protocol available and pre-specified outcomes were reported |
| Other bias | High risk | Sponsor included in data management and analysis: Novartis Pharma and authorship |

1. **Dooley 2011**

| Bias | Authors’ judgement | Support of judgement |
| --- | --- | --- |
| Random sequence generation (selection bias) | Unclear risk | Participants randomly assigned (1:1, stratified by race and biopsy class, non-blocked) but sequence of generation is not reported |
| Allocation concealment (selection bias) | Low risk | Central, computerised, interactive voice response system. Method would not allow investigator/participant to know or influence intervention group |
| Blinding of participants and personnel (performance bias) | High risk | Open-label study |
| Blinding of outcome assessment (detection bias) | Low risk | Primary outcome assessed by blinded Clinical EndPoints Committee |
| Incomplete outcome data (attrition bias) | Low risk | No missing outcome data |
| Selective reporting (reporting bias) | Low risk | Study protocol available and pre-specified outcomes were reported |
| Other bias | High risk | Sponsored by Aspreva Pharmaceuticals Corporation included in the data analysis & authorship |

1. **Chen 2012**

| Bias | Authors’ judgement | Support of judgement |
| --- | --- | --- |
| Random sequence generation (selection bias) | Low risk | Randomisation was conducted at a central office using a computer-based random allocation sequence table; randomisation not stratified by centre or baseline characteristic |
| Allocation concealment (selection bias) | Low risk | Allocation concealment performed by enclosing assignments in sequentially numbered, opaque, closed envelopes |
| Blinding of participants and personnel (performance bias) | High risk | Open-label study |
| Blinding of outcome assessment (detection bias) | Unclear risk | Insufficient information to permit judgement |
| Incomplete outcome data (attrition bias) | Low risk | The primary outcome (complete remission) and secondary outcomes partial remission and treatment failure were reported on an intention to treat bases. |
| Selective reporting (reporting bias) | Low risk | Study protocol available and pre-specified outcomes were reported |
| Other bias | Low risk | Astellas Pharmaceutics supplied TAC but had no role in the design or conduct of the study or analysis or interpretation of results |

1. **Kaballo 2016**

| Bias | Authors’ judgement | Support of judgement |
| --- | --- | --- |
| Random sequence generation (selection bias) | Low risk | Patients stratified by block randomisation (stratification factors were gender, age and weight) |
| Allocation concealment (selection bias) | Unclear risk | Insufficient information to permit judgement |
| Blinding of participants and personnel (performance bias) | Unclear risk | Insufficient information to permit judgement |
| Blinding of outcome assessment (detection bias) | Unclear risk | Insufficient information to permit judgement |
| Incomplete outcome data (attrition bias) | Low risk | No missing outcome data |
| Selective reporting (reporting bias) | Low risk | Trial registration was not reported, all expected outcomes were reported |
| Other bias | Low risk | The study appears to be free of other sources of bias |

1. **Contreras 2004**

| Bias | Authors’ judgement | Support of judgement |
| --- | --- | --- |
| Random sequence generation (selection bias) | High risk | After induction, participants were randomly assigned, in order of enrolment by means of sealed envelopes (stratified in two groups: blacks and other participants). -consecutive sequence generation |
| Allocation concealment (selection bias) | Low risk | Sealed envelopes used |
| Blinding of participants and personnel (performance bias) | High risk | Open-label study |
| Blinding of outcome assessment (detection bias) | Unclear risk | Insufficient information to permit judgement |
| Incomplete outcome data (attrition bias) | Low risk | No missing outcome data |
| Selective reporting (reporting bias) | Low risk | Study protocol available and pre-specified outcomes were reported |
| Other bias | High risk | Roche pharmaceutical providing research nurse support and MMF 1999 to 2003. Authors received fees for lectures and a grant from Roche Pharmaceuticals |

1. **Houssiau 2010**

| Bias | Authors’ judgement | Support of judgement |
| --- | --- | --- |
| Random sequence generation (selection bias) | Unclear risk | Study was described as randomised, method of randomisation was not reported |
| Allocation concealment (selection bias) | Unclear risk | Insufficient information to permit judgement |
| Blinding of participants and personnel (performance bias) | High risk | Open-label study |
| Blinding of outcome assessment (detection bias) | Unclear risk | Insufficient information to permit judgement |
| Incomplete outcome data (attrition bias) | Low risk | No missing outcome data |
| Selective reporting (reporting bias) | Low risk | Study protocol available and pre-specified outcomes were reported |
| Other bias | Unclear risk | Insufficient information to permit judgement |

1. **Carette 1983**

| Bias | Authors’ judgement | Support of judgement |
| --- | --- | --- |
| Random sequence generation (selection bias) | Low risk | drawing marked card sequence from a table of random numbers |
| Allocation concealment (selection bias) | Unclear risk | Insufficient information to permit judgement |
| Blinding of participants and personnel (performance bias) | Unclear risk | Insufficient information to permit judgement |
| Blinding of outcome assessment (detection bias) | Unclear risk | Insufficient information to permit judgement |
| Incomplete outcome data (attrition bias) | Low risk | 3.6% (4/111) of participants excluded as they did not complete 3 months of treatment |
| Selective reporting (reporting bias) | Low risk | All expected outcomes were reported |
| Other bias | High risk | Patients were assigned to treatment groups 1, 2 and 3 from the beginning of the study (1969). Treatment groups 4 and 5 were introduced in January 1973. |

1. **Chan 2000**

| Bias | Authors’ judgement | Support of judgement |
| --- | --- | --- |
| Random sequence generation (selection bias) | Low risk | Participants randomly assigned by drawing envelopes to one of two treatment groups in an open-label manner |
| Allocation concealment (selection bias) | Unclear risk | Insufficient information to permit judgement |
| Blinding of participants and personnel (performance bias) | Unclear risk | Insufficient information to permit judgement |
| Blinding of outcome assessment (detection bias) | Low risk | Clinical status was reviewed and categorised at the coordinating centre by personnel who had no knowledge of the treatment assignment |
| Incomplete outcome data (attrition bias) | Low risk | No missing outcome data |
| Selective reporting (reporting bias) | Low risk | Study protocol available and pre-specified outcomes were reported |
| Other bias | Low risk | The study appears to be free of other sources of bias |

1. **Grootscholten 2006**

| Bias | Authors’ judgement | Support of judgement |
| --- | --- | --- |
| Random sequence generation (selection bias) | Low risk | Randomisation performed at a central office with a computer program, using the minimisation determinants: centre, SCr (<150 or > 150 µmol/L), WHO class III or IV, previous treatment with immunosuppressive medication for lupus nephritis |
| Allocation concealment (selection bias) | Unclear risk | Central office with computer program. Not sufficiently clear to determine risk |
| Blinding of participants and personnel (performance bias) | High risk | Open-label study |
| Blinding of outcome assessment (detection bias) | Unclear risk | Insufficient information to permit judgement |
| Incomplete outcome data (attrition bias) | Low risk | No missing outcome data |
| Selective reporting (reporting bias) | Low risk | Study protocol available and pre-specified outcomes were reported |
| Other bias | Low risk | Funding from Dutch Kidney Foundation and Dutch League against Rheumatism. One author disclosed speaking fees from Novartis. The study appears to be free of other sources of bias |

1. **Appel 2009**

| Bias | Authors’ judgement | Support of judgement |
| --- | --- | --- |
| Random sequence generation (selection bias) | Unclear risk | Participants randomly assigned (1:1, stratified by race and biopsy class, non-blocked) but sequence of generation is not reported |
| Allocation concealment (selection bias) | Low risk | Central, computerised, interactive voice response system. Method would not allow investigator/participant to know or influence intervention group |
| Blinding of participants and personnel (performance bias) | High risk | Induction therapy - Open-label study; maintenance therapy - double-blind |
| Blinding of outcome assessment (detection bias) | Low risk | Primary outcome assessed by blinded Clinical EndPoints Committee |
| Incomplete outcome data (attrition bias) | Low risk | No missing outcome data; Induction therapy (group 1: 1 lost to follow-up; group 2: 2 lost to follow-up) |
| Selective reporting (reporting bias) | Low risk | Study protocol available and pre-specified outcomes were reported |
| Other bias | High risk | Sponsored by Aspreva Pharmaceuticals Corporation included in the data analysis & authorship |

1. **Zavada 2010**

| Bias | Authors’ judgement | Support of judgement |
| --- | --- | --- |
| Random sequence generation (selection bias) | Unclear risk | Randomisation 1:1, non-blocked methods for sequence generation not reported |
| Allocation concealment (selection bias) | Low risk | Central computerised system |
| Blinding of participants and personnel (performance bias) | High risk | Open-label study |
| Blinding of outcome assessment (detection bias) | Unclear risk | Insufficient information to permit judgement |
| Incomplete outcome data (attrition bias) | Low risk | No missing outcome data |
| Selective reporting (reporting bias) | Low risk | Study protocol available and pre-specified outcomes were reported |
| Other bias | Low risk | Research grants from the IGA Ministry of Health, Czech Republic. The study appears to be free of other sources of bias |

1. **Mok 2001**

| Bias | Authors’ judgement | Support of judgement |
| --- | --- | --- |
| Random sequence generation (selection bias) | Unclear risk | Study was described as randomised, method of randomisation was not reported |
| Allocation concealment (selection bias) | Unclear risk | Insufficient information to permit judgement |
| Blinding of participants and personnel (performance bias) | High risk | Open-label study |
| Blinding of outcome assessment (detection bias) | Unclear risk | Insufficient information to permit judgement |
| Incomplete outcome data (attrition bias) | Unclear risk | Insufficient information to permit judgement |
| Selective reporting (reporting bias) | Unclear risk | Insufficient information to permit judgement |
| Other bias | Unclear risk | Insufficient information to permit judgement |

1. **Yee 2004**

| Bias | Authors’ judgement | Support of judgement |
| --- | --- | --- |
| Random sequence generation (selection bias) | Low risk | Participants were stratified according to the presence of kidney failure and underwent block randomisation to either therapy |
| Allocation concealment (selection bias) | Unclear risk | Insufficient information to permit judgement |
| Blinding of participants and personnel (performance bias) | High risk | Open-label study |
| Blinding of outcome assessment (detection bias) | Unclear risk | Insufficient information to permit judgement |
| Incomplete outcome data (attrition bias) | Low risk | No missing outcome data |
| Selective reporting (reporting bias) | High risk | Not all pre-specified outcomes reported: alopecia |
| Other bias | High risk | Study was terminated after four years as patient recruitment was disappointing and many patients had been withdrawn; Many physicians became reluctant to enter patients because of concerns that the oral regimen was slower to work and more toxic than the pulse regimen, following development of severe neutropenia in the continuous group; This led to the premature termination of the study |

1. **Furie 2014**

| Bias | Authors’ judgement | Support of judgement |
| --- | --- | --- |
| Random sequence generation (selection bias) | Unclear risk | Study was described as randomised, method of randomisationwas not reported, however patients were stratified according to prior treatment |
| Allocation concealment (selection bias) | Unclear risk | Insufficient information to permit judgement |
| Blinding of participants and personnel (performance bias) | Low risk | Double-blind, double dummy placebo study |
| Blinding of outcome assessment (detection bias) | Unclear risk | Insufficient information to permit judgement |
| Incomplete outcome data (attrition bias) | High risk | Not all relevant reported outcomes are reported |
| Selective reporting (reporting bias) | Low risk | Study protocol available and pre-specified outcomes were reported |
| Other bias | High risk | Sponsor included in data analysis/authorship |

1. **Furie 2020**

| Bias | Authors’ judgement | Support of judgement |
| --- | --- | --- |
| Random sequence generation (selection bias) | Low risk | Randomly assigned in a 1:1 ratio with the use of an interactive Web­response system |
| Allocation concealment (selection bias) | Low risk | The trial agents were prepared by pharmacists who were aware of the trial­group assignments. Patients and staff were unaware of the trial­group assignments, although independent monitors were aware of these assignments |
| Blinding of participants and personnel (performance bias) | Low risk | Double-blind, placebo-controlled study |
| Blinding of outcome assessment (detection bias) | Low risk | An independent data and safety monitoring committee provided an ongoing review of safety data |
| Incomplete outcome data (attrition bias) | Low risk | All relevant reported outcomes are reported |
| Selective reporting (reporting bias) | Low risk | Study protocol available and pre-specified outcomes were reported |
| Other bias | Low risk | The study appears to be free of other sources of bias |

1. **Atisha-Fregoso 2021**

| Bias | Authors’ judgement | Support of judgement |
| --- | --- | --- |
| Random sequence generation (selection bias) | Low risk | Randomization at Week 4 was 1:1 using a permuted block design |
| Allocation concealment (selection bias) | Unclear risk | Insufficient information to permit judgement |
| Blinding of participants and personnel (performance bias) | High risk | Open-label study |
| Blinding of outcome assessment (detection bias) | Unclear risk | Insufficient information to permit judgement |
| Incomplete outcome data (attrition bias) | Low risk | No missing outcome data |
| Selective reporting (reporting bias) | Unclear risk | Insufficient information to permit judgement |
| Other bias | Unclear risk | Insufficient information to permit judgement |

1. **Ye 2022**

| Bias | Authors’ judgement | Support of judgement |
| --- | --- | --- |
| Random sequence generation (selection bias) | Unclear risk | Study was described as randomised, method of randomisation was not reported |
| Allocation concealment (selection bias) | Unclear risk | Insufficient information to permit judgement |
| Blinding of participants and personnel (performance bias) | High risk | Open-label study |
| Blinding of outcome assessment (detection bias) | Unclear risk | Insufficient information to permit judgement |
| Incomplete outcome data (attrition bias) | Low risk | All relevant reported outcomes are reported |
| Selective reporting (reporting bias) | Unclear risk | Insufficient information to permit judgement |
| Other bias | Unclear risk | Insufficient information to permit judgement |
