# Supplementary material for: Efficacy and safety of immunosuppressive agents for adults with lupus nephritis: a systematic review and network meta-analysis
Source: Front Immunol. 2023 Oct 13;14:1232244. doi: 10.3389/fimmu.2023.1232244 (PMC10611487; doi:10.3389/fimmu.2023.1232244)
Supplement: Supplementary file 1 [file DataSheet_1.zip › Supplement 5.docx]

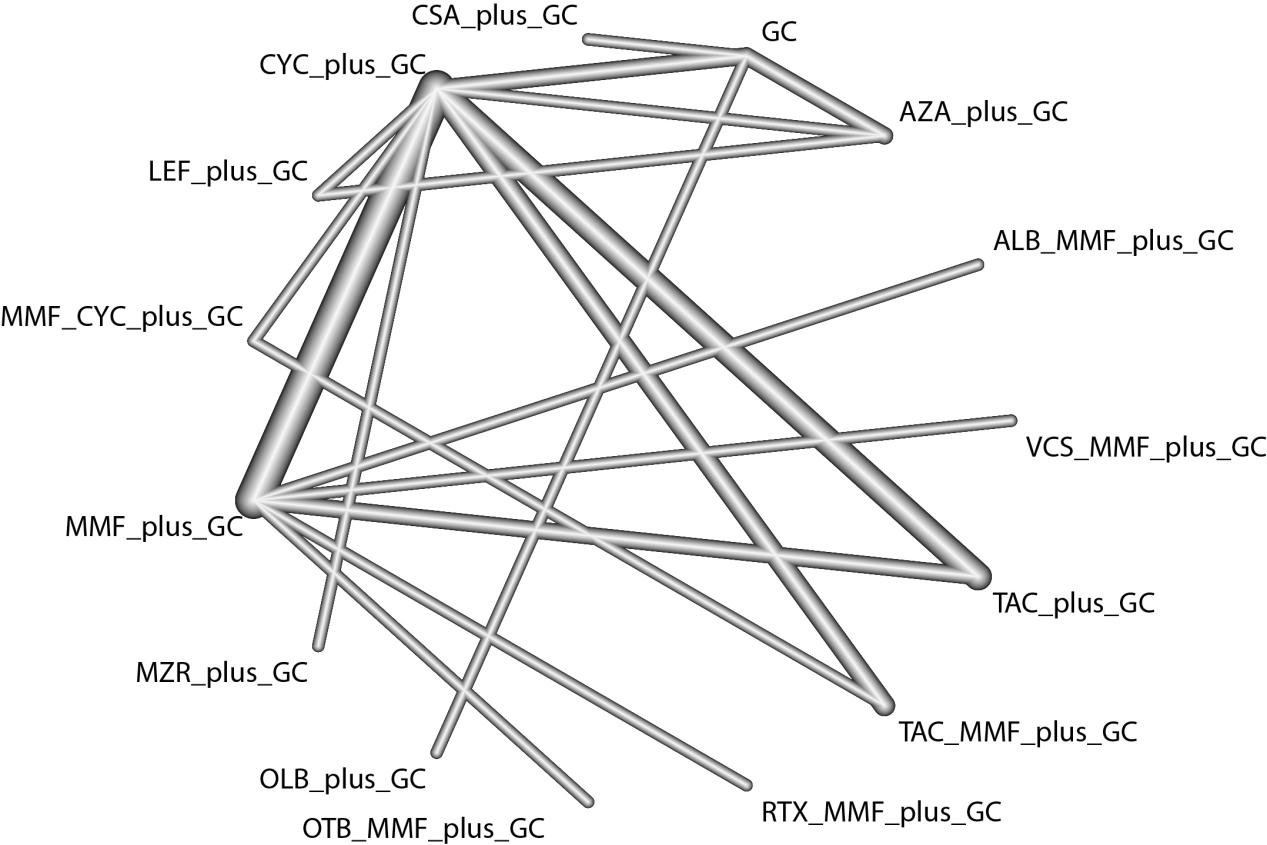


Figure S1. Network comparisons for complete remission rate included in the analysis.


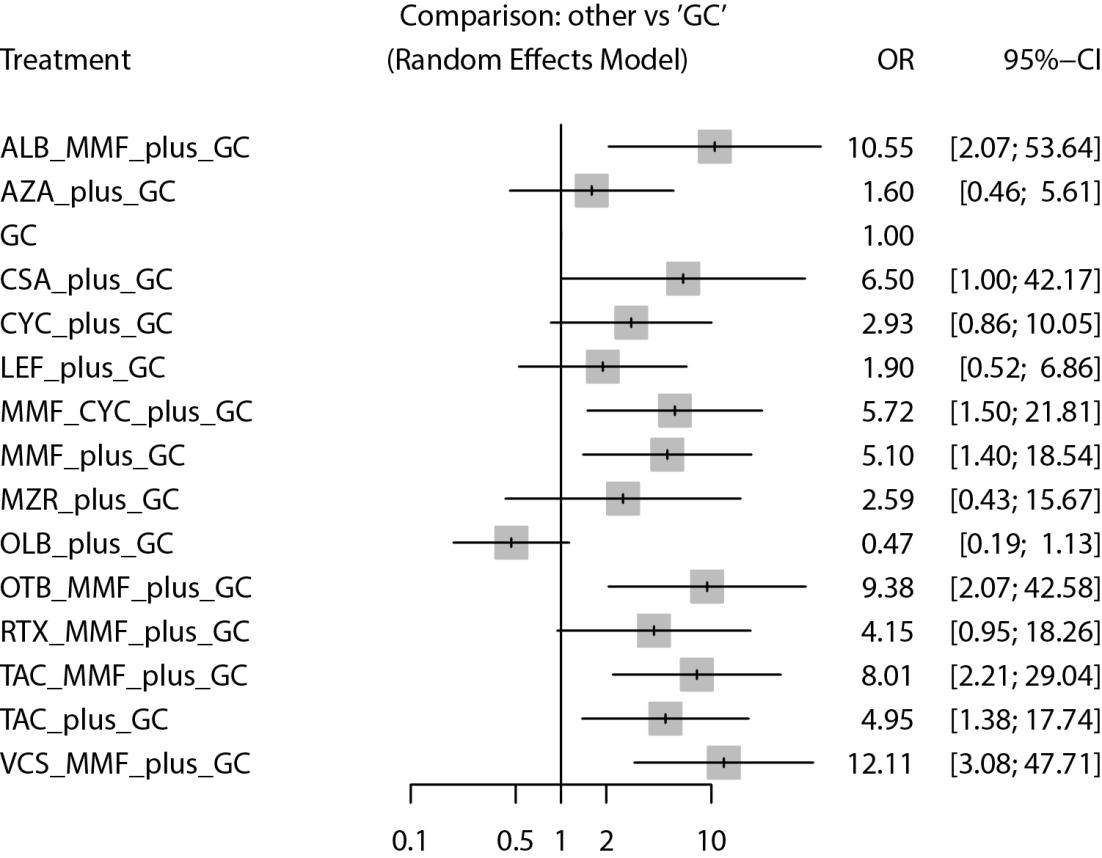


Figure S2. Treatment regimens versus GC on complete remission rate.


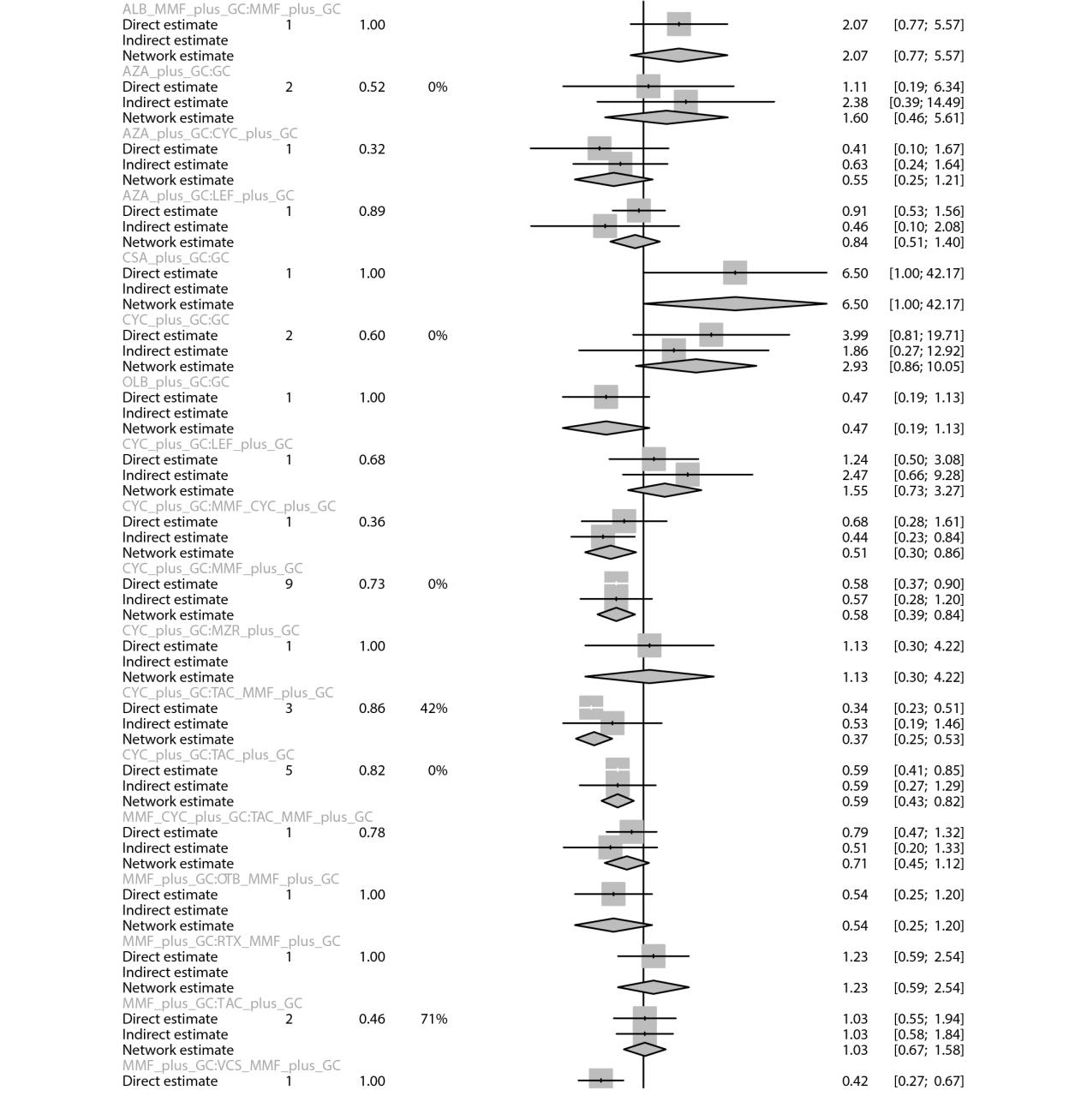


Figure S3. Pairwise comparison of treatment regimens for complete remission rate


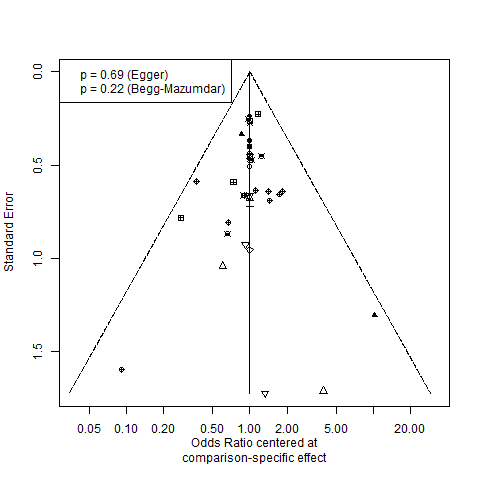


Figure S4. Funnel plot for complete remission rate
