# Supplementary material for: Efficacy and safety of immunosuppressive agents for adults with lupus nephritis: a systematic review and network meta-analysis
Source: Front Immunol. 2023 Oct 13;14:1232244. doi: 10.3389/fimmu.2023.1232244 (PMC10611487; doi:10.3389/fimmu.2023.1232244)
Supplement: Supplementary file 1 [file DataSheet_1.zip › Supplement 7.docx]

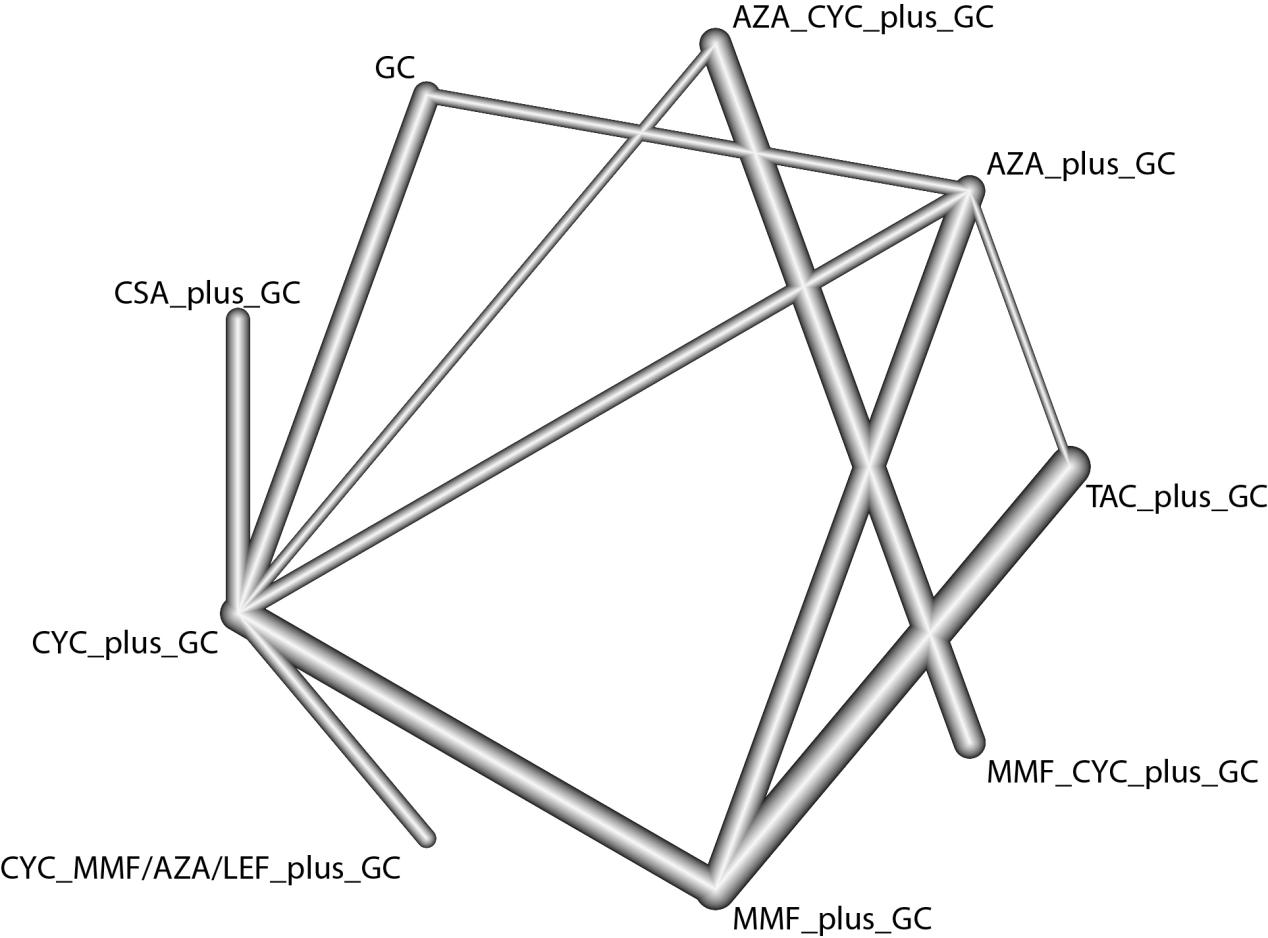


Figure S1. Network comparisons for relapse included in the analysis


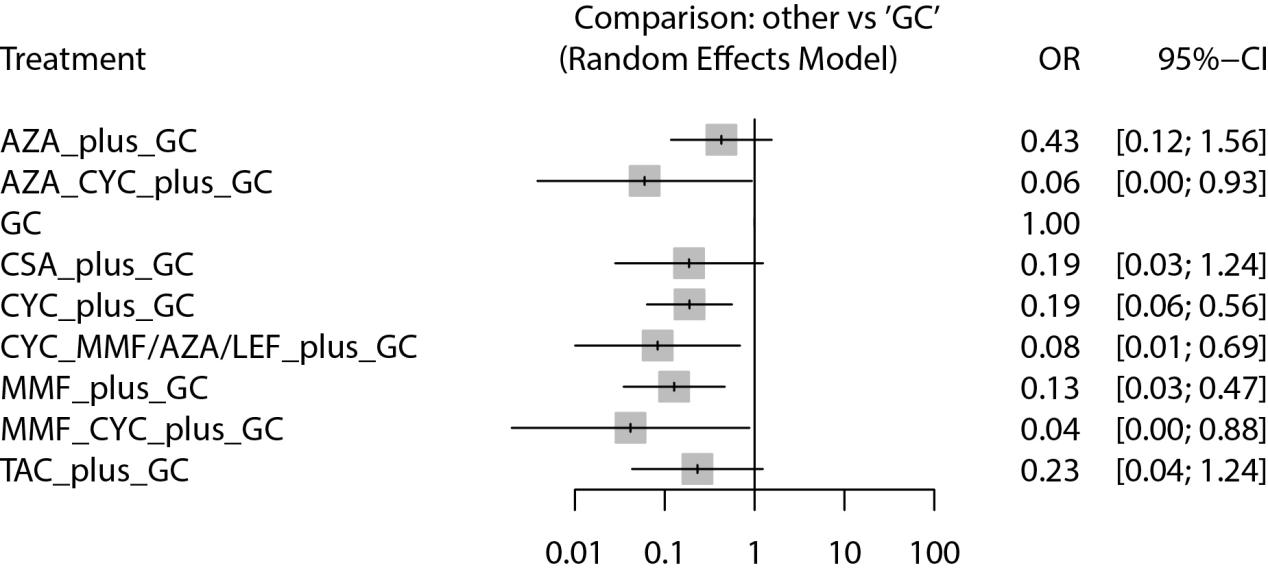


Figure S2. Treatment regimens versus GC on relapse.


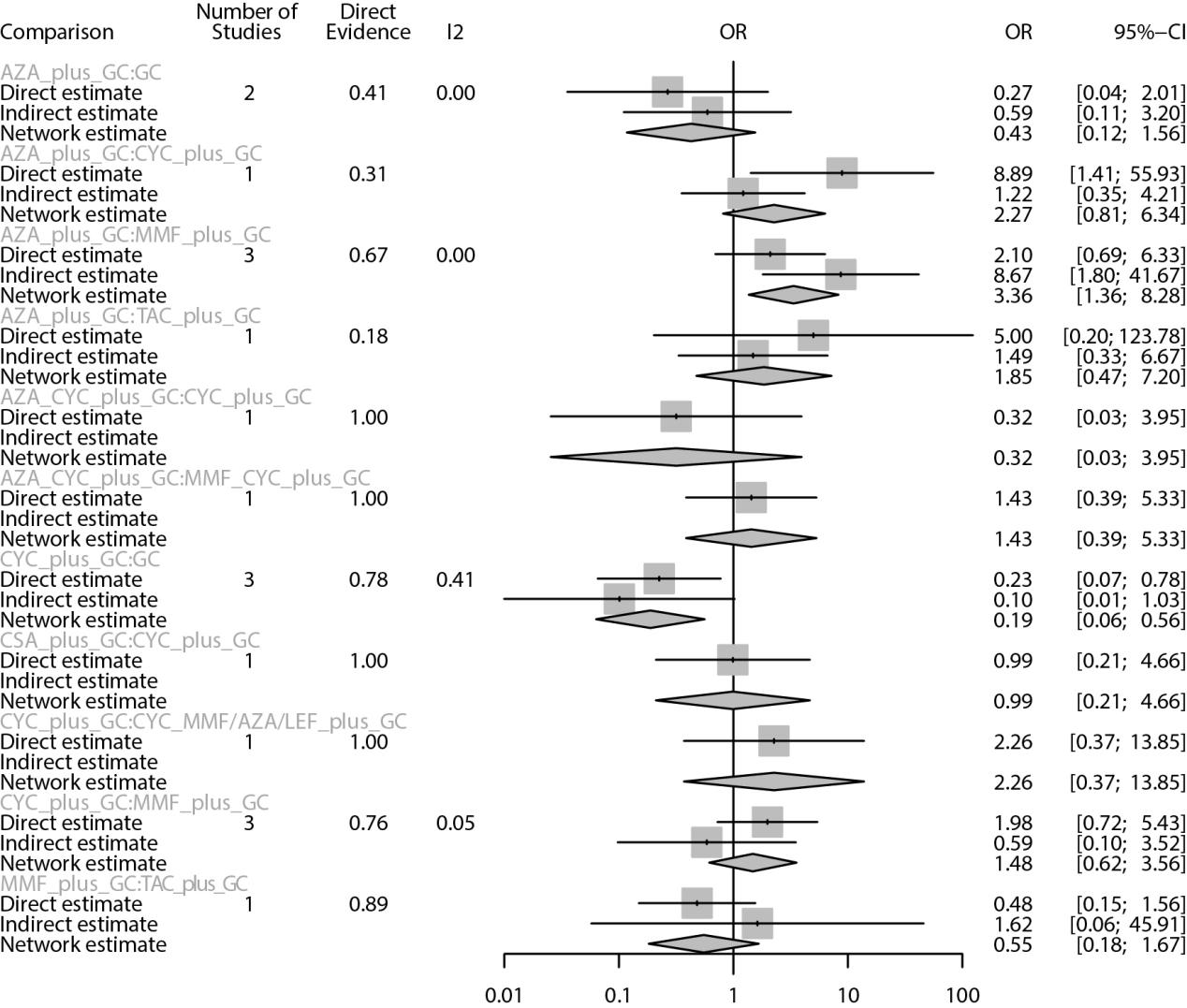


Figure S3. Pairwise comparison of treatment regimens for relapse


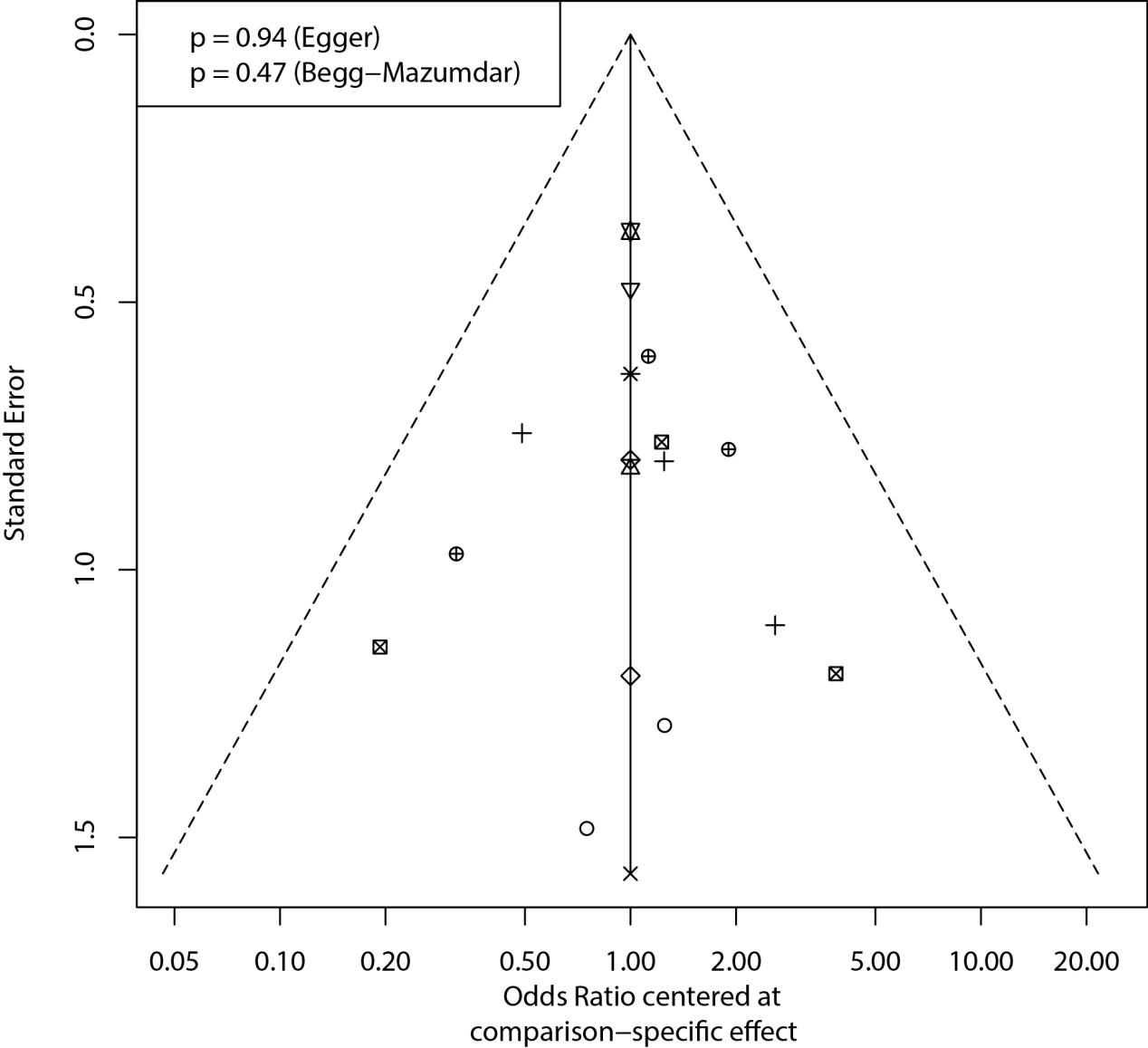


Figure S4. Funnel plot for relapse
