# Supplementary material for: Efficacy and safety of immunosuppressive agents for adults with lupus nephritis: a systematic review and network meta-analysis
Source: Front Immunol. 2023 Oct 13;14:1232244. doi: 10.3389/fimmu.2023.1232244 (PMC10611487; doi:10.3389/fimmu.2023.1232244)
Supplement: Supplementary file 1 [file DataSheet_1.zip › Supplement 8.docx]

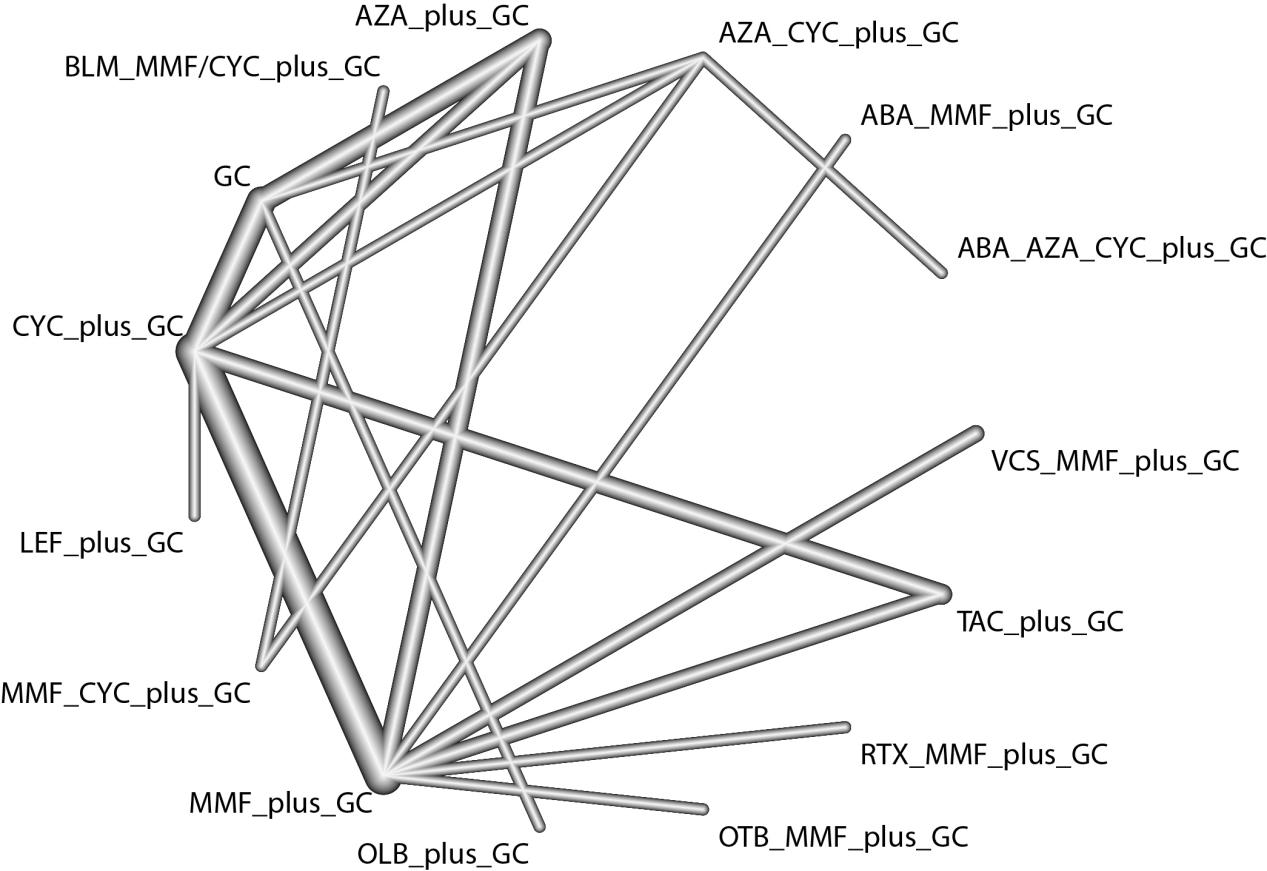


Figure S1. Network comparisons for all-cause mortality included in the analysis


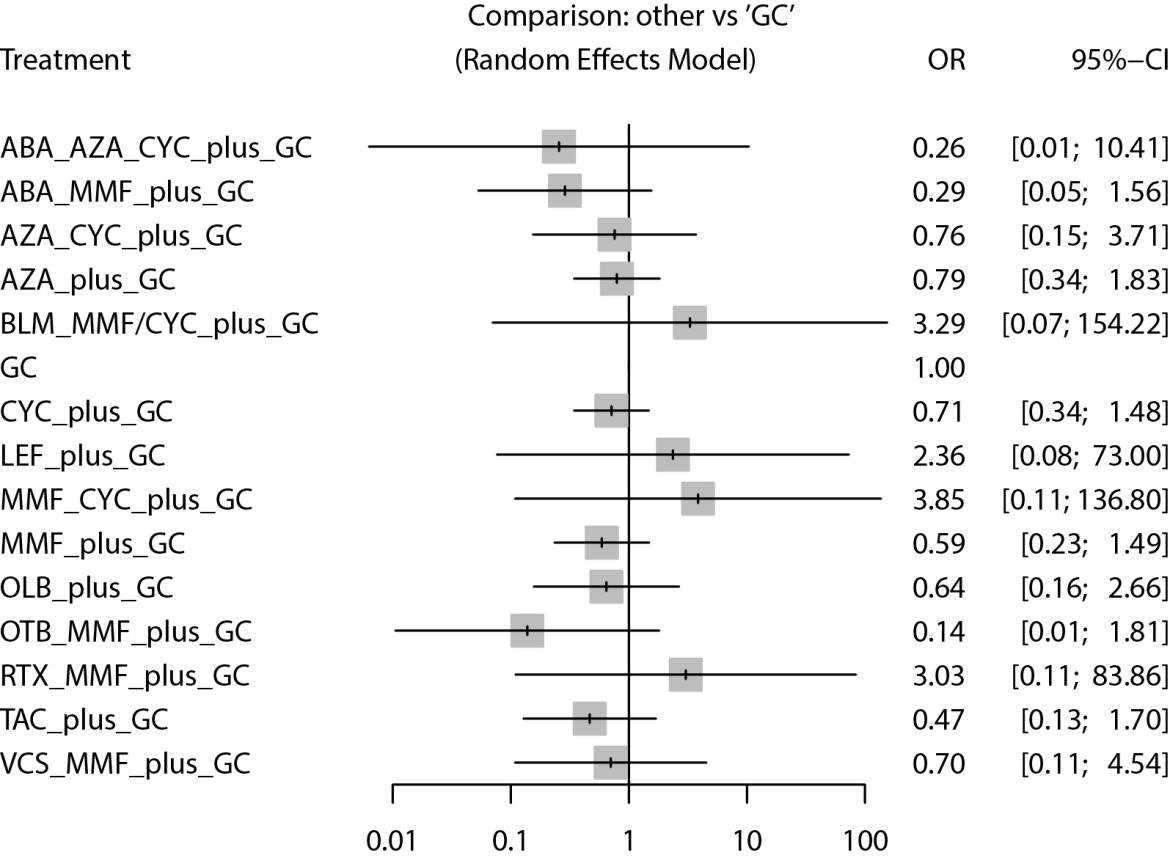


Figure S2. Treatment regimens versus GC on all-cause mortality.


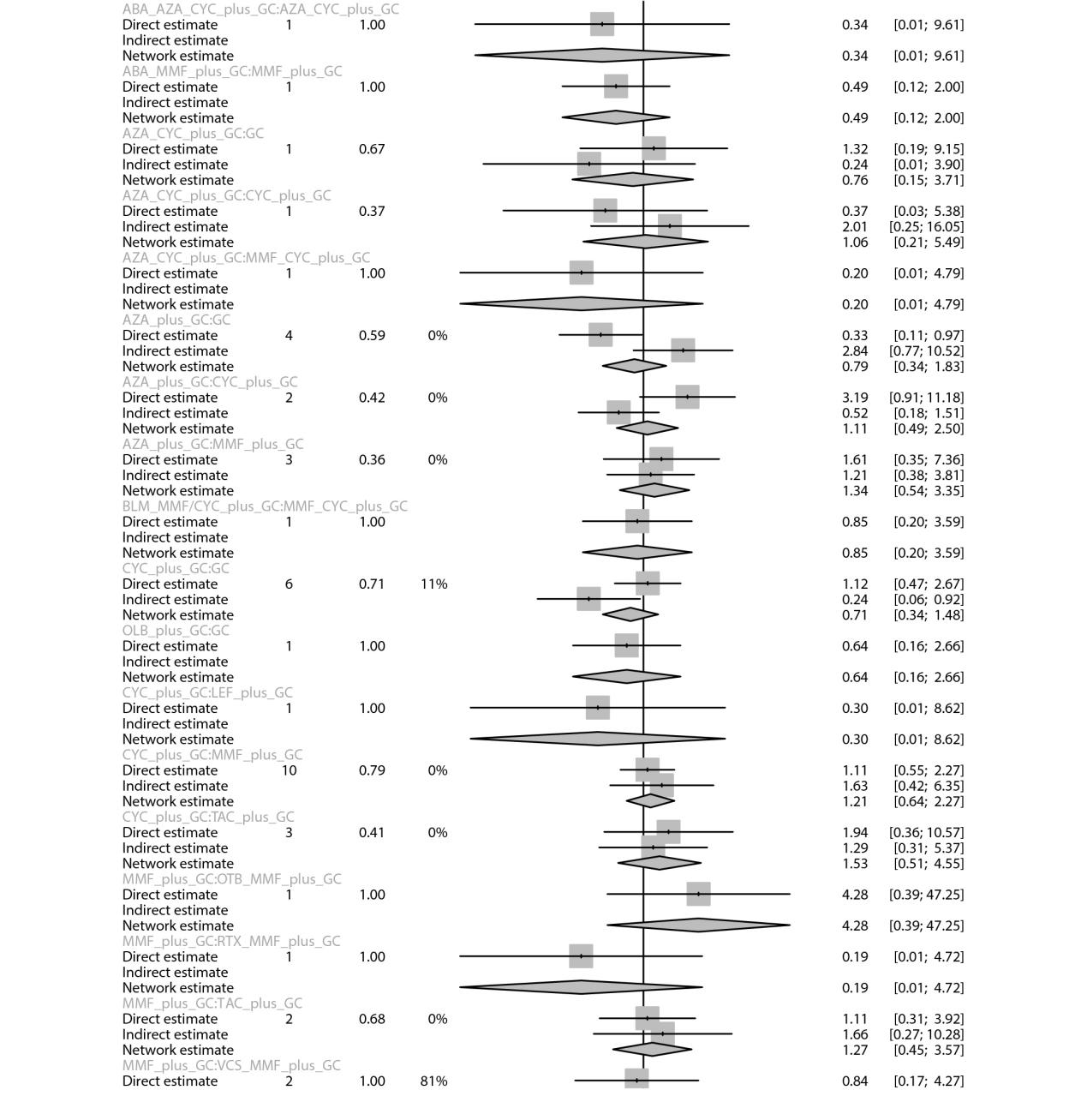


Figure S3. Pairwise comparison of treatment regimens for all-cause mortality


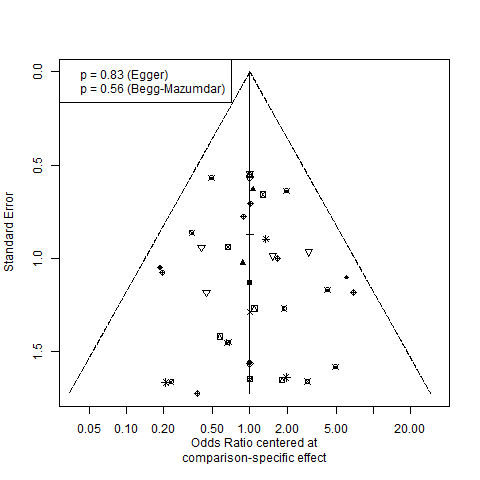


Figure S4. Funnel plot for all-cause mortality
