# Supplementary material for: Efficacy and safety of immunosuppressive agents for adults with lupus nephritis: a systematic review and network meta-analysis
Source: Front Immunol. 2023 Oct 13;14:1232244. doi: 10.3389/fimmu.2023.1232244 (PMC10611487; doi:10.3389/fimmu.2023.1232244)
Supplement: Supplementary file 1 [file DataSheet_1.zip › Supplement 10.docx]

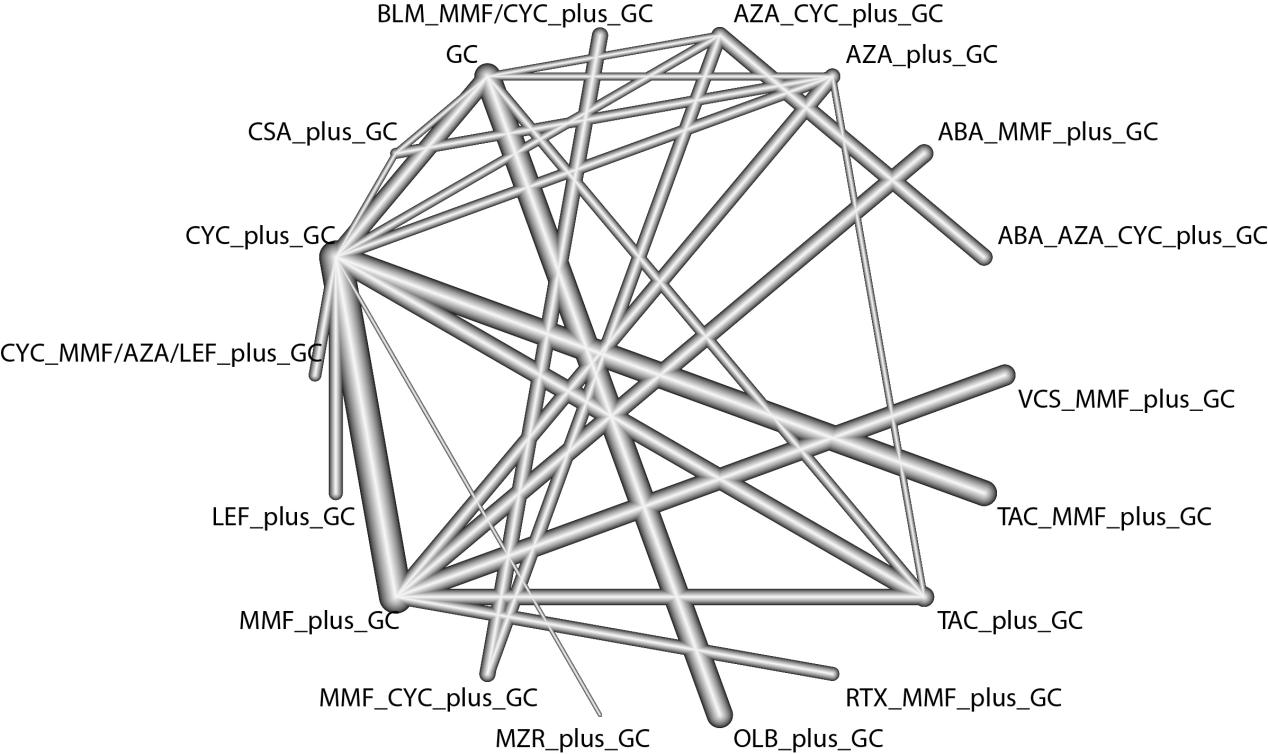


Figure S1. Network comparisons for infection risk included in the analysis.


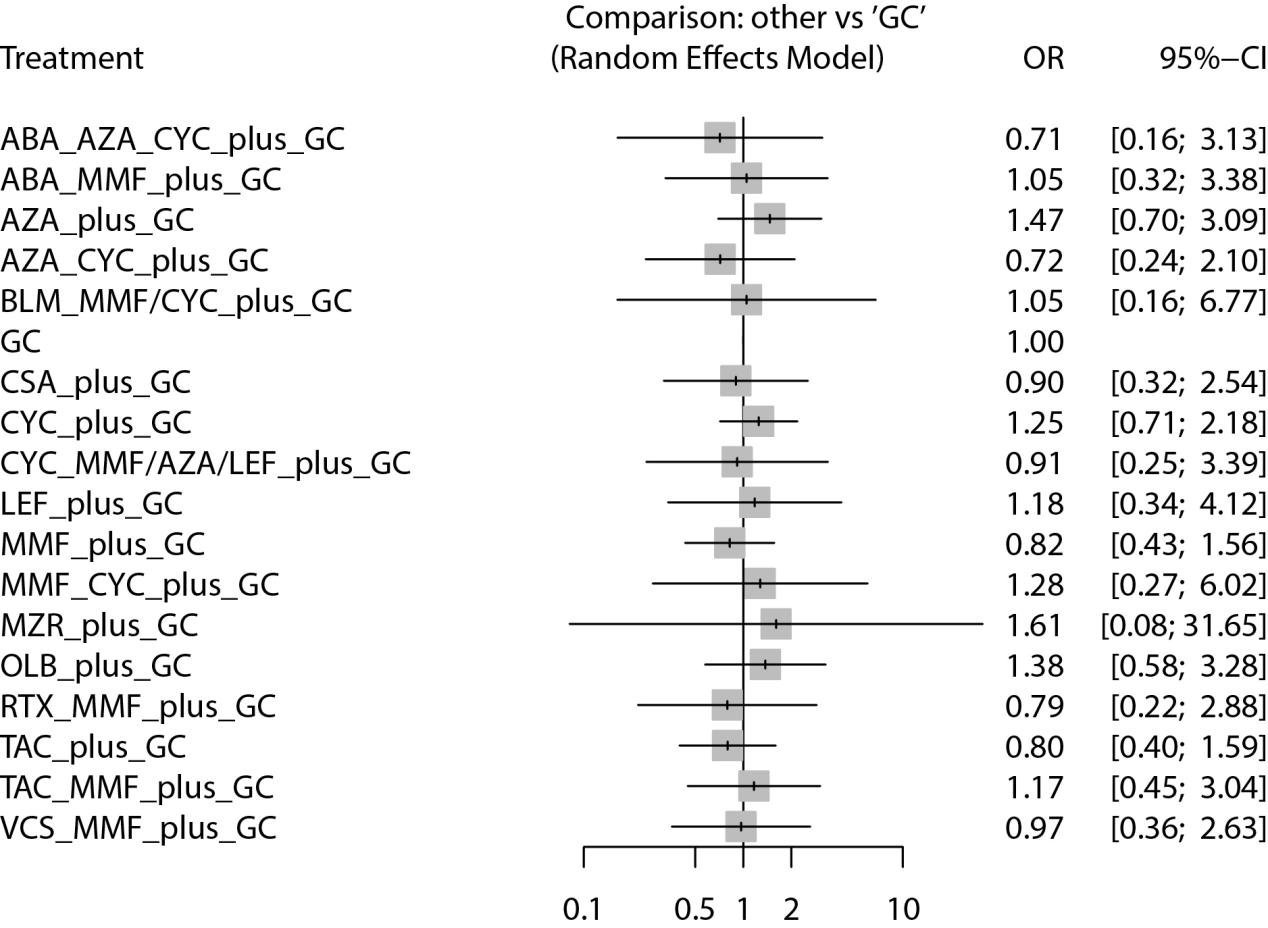


Figure S2. Treatment regimens versus GC on infection risk.


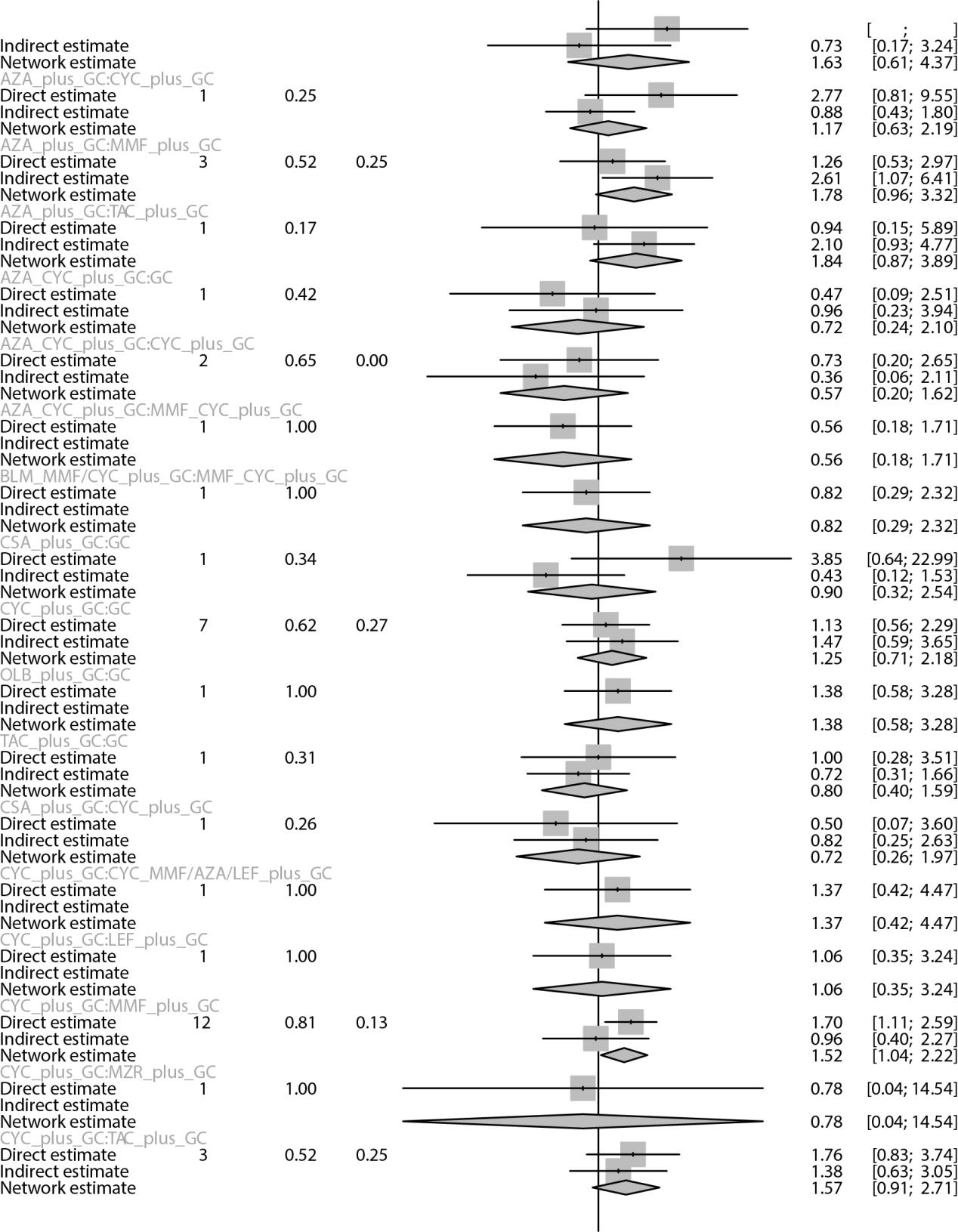


Figure S3. Pairwise comparison of treatment regimens for infection risk


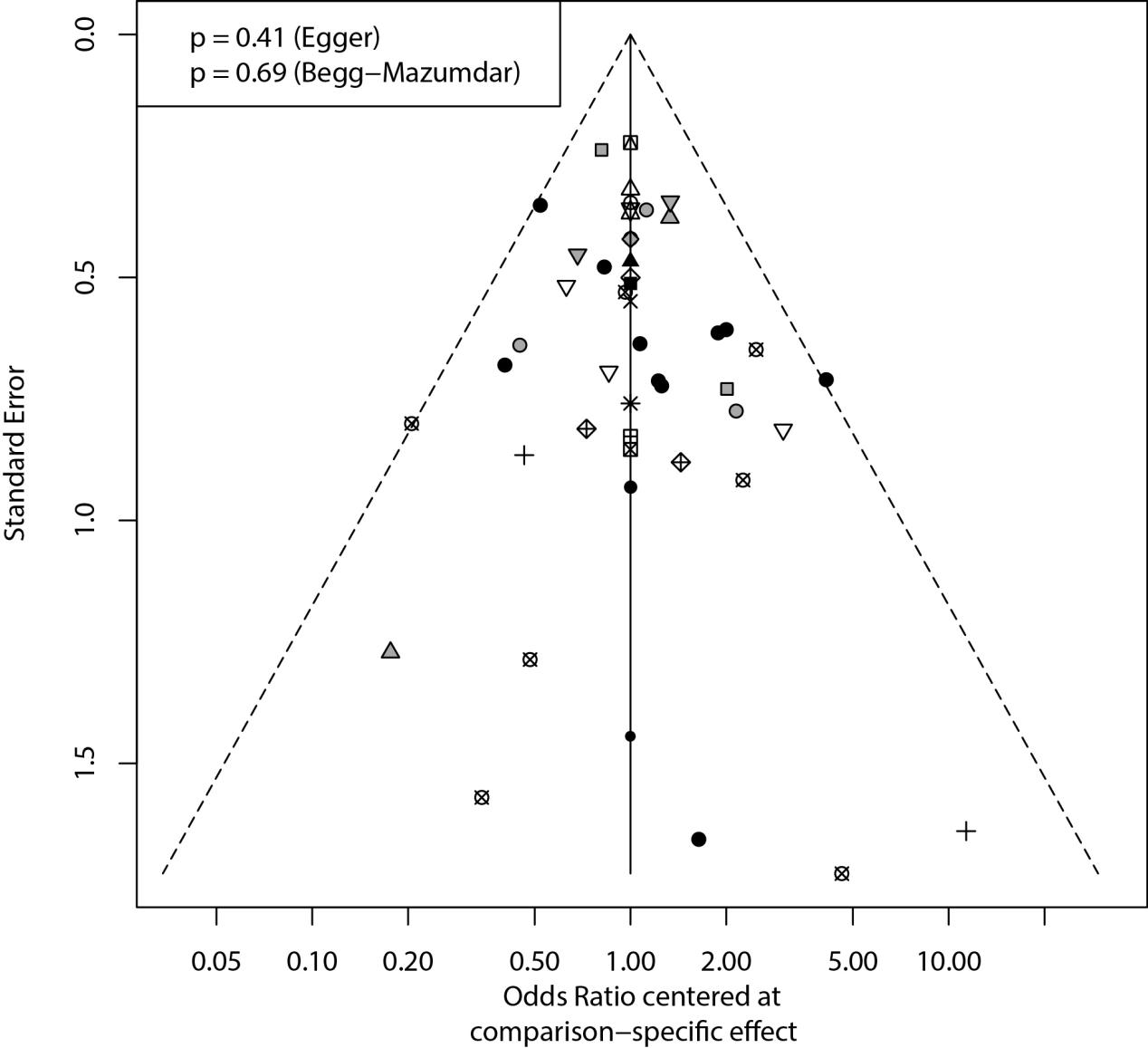


Figure S4. Funnel plot for infection risk
