# Supplementary material for: Efficacy and safety of immunosuppressive agents for adults with lupus nephritis: a systematic review and network meta-analysis
Source: Front Immunol. 2023 Oct 13;14:1232244. doi: 10.3389/fimmu.2023.1232244 (PMC10611487; doi:10.3389/fimmu.2023.1232244)
Supplement: Supplementary file 1 [file DataSheet_1.zip › Supplement 13.docx]

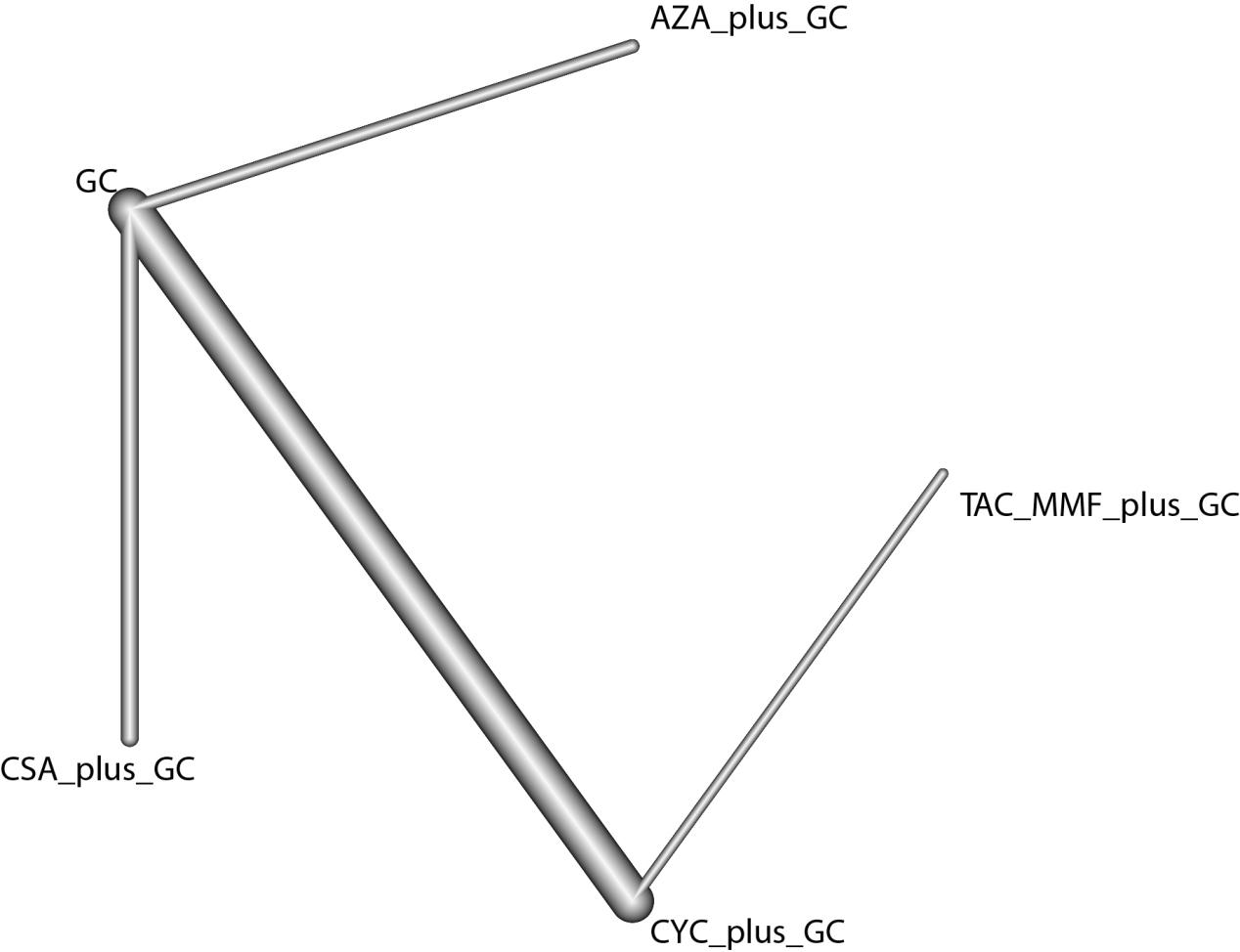


Figure S1. Network comparisons for myelosuppression included in the analysis


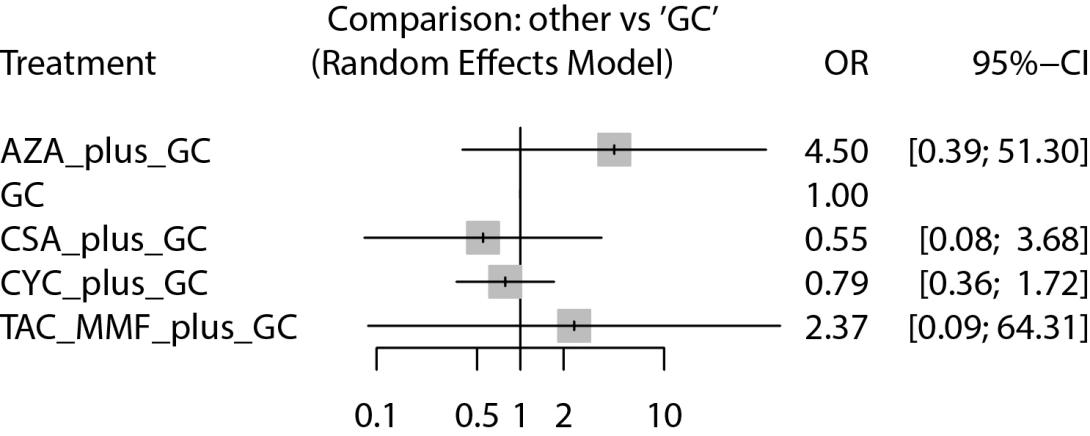


Figure S2. Treatment regimens versus GC on myelosuppression


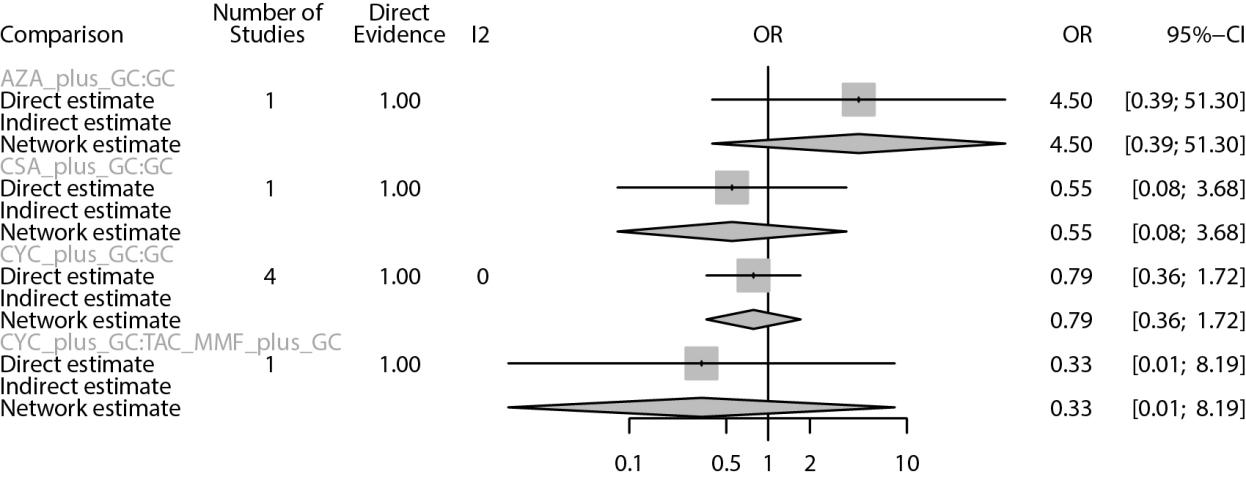


Figure S3. Pairwise comparison of treatment regimens for myelosuppression


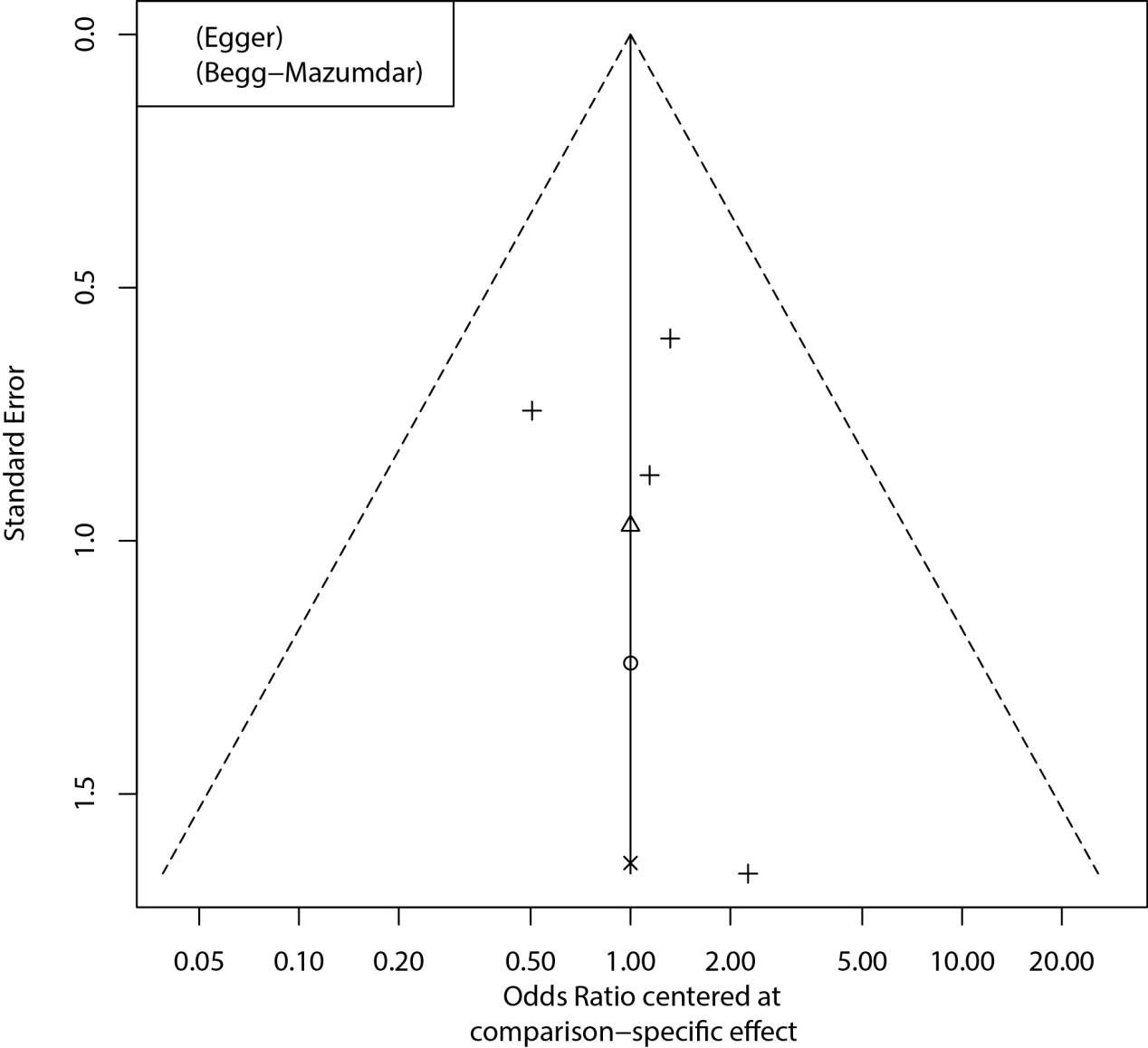


Figure S4. Funnel plot for myelosuppression
